# Diene hydroaminomethylation *via* ruthenium-catalyzed C–C bond forming transfer hydrogenation: beyond carbonylation†
†Electronic supplementary information (ESI) available: Experimental procedures and spectral data for new compounds, including scanned images of ^1^H and ^13^C NMR spectra. Single crystal X-ray diffraction data for a derivative of **5a**. CCDC 1430833. For ESI and crystallographic data in CIF or other electronic format see DOI: 10.1039/c5sc03854e


**DOI:** 10.1039/c5sc03854e

**Published:** 2015-11-17

**Authors:** Susumu Oda, Jana Franke, Michael J. Krische

**Affiliations:** a Department of Chemistry , University of Texas at Austin , Austin , Texas , 78712 USA . Email: mkrische@mail.utexas.edu

## Abstract

Under the conditions of ruthenium catalyzed transfer hydrogenation using 2-propanol as terminal reductant, 1,3-dienes engage in reductive C–C coupling with formaldimines obtained *in situ* from 1,3,5-tris(aryl)-hexahydro-1,3,5-triazines to form homoallylic amines. Deuterium labelling studies corroborate a mechanism involving reversible diene hydroruthenation to form an allylruthenium complex that engages in turn-over limiting imine addition. Protonolysis of the resulting amidoruthenium species releases product and delivers a ruthenium alkoxide, which upon β-hydride elimination closes the catalytic cycle. These transformations, which include enantioselective variants, represent the first examples of diene hydroaminomethylation.

## Abstract 2

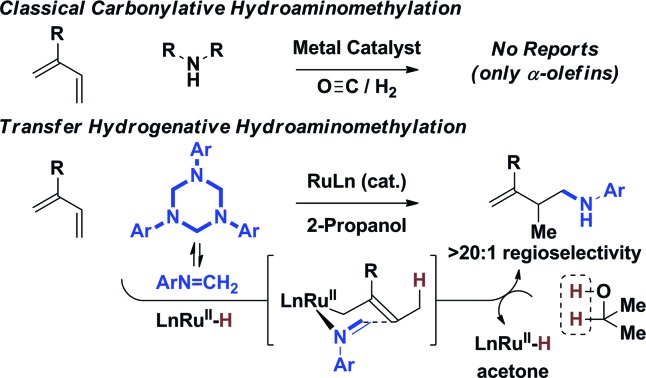
Ruthenium catalyzed transfer hydrogenation of dienes in the presence of formaldimines delivers products of hydroaminomethylation as single regioisomers.

## Introduction

**Scheme 1:**
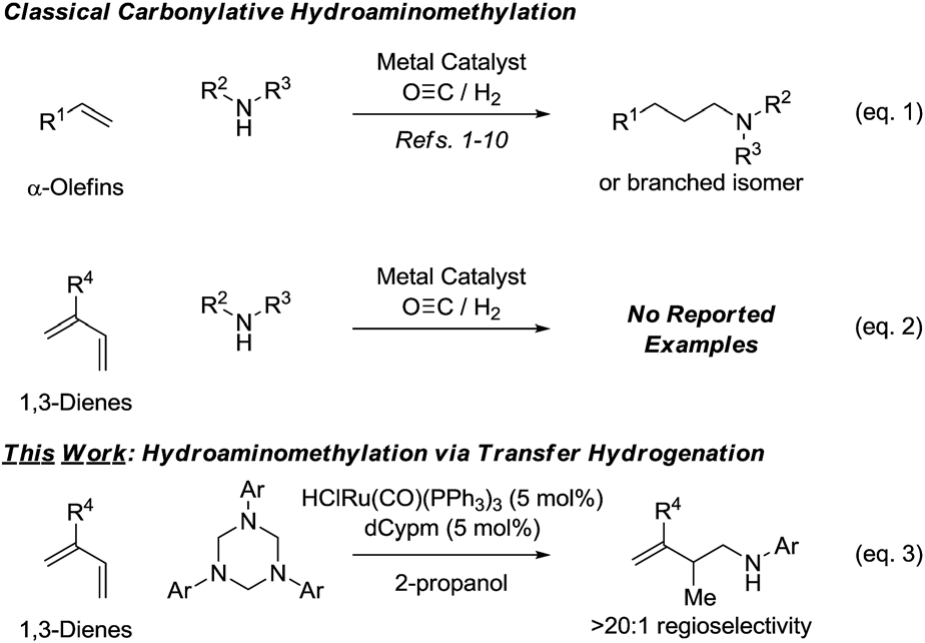
Hydroaminomethylation *via* carbonylation or 2-propanol mediated reductive coupling.

Rhodium catalyzed hydroformylation-reductive amination or “hydroaminomethylation”^1^ of α-olefins (Scheme 1, eqn (1)) has emerged as an important method for the synthesis of *N*-containing compounds, including pharmaceutical ingredients (*e.g.* cinacalcet,^2^,3a ibutilide,^2^,3b and fexofenadine^2^,3c). Following its discovery at BASF in 1949 by Reppe,^4^ hydroaminomethylation initially received only a modest level of attention from academic and industrial researchers.^5^ The systematic studies of Eilbracht in the late 1990's^6^ brought rhodium catalyzed hydroaminomethylation to the forefront of research, and in the last 15 years significant progress in this area was made. Notable achievements include the design of catalytic systems enabling direct use of ammonia,6c,^7^ the ability to control regioselectivity in reactions of terminal8*a* as well as internal8*b* alkenes *via* ligand control^8^ or use of directing groups,^9^ and the development of the first *ruthenium* catalyzed carbonylative hydroaminomethylations.^10^

Despite these advances, existing catalysts for hydroaminomethylation *via* hydroformylation-reductive amination are restricted to the use of nonconjugated alkenes, typically α-olefins. The carbonylative hydroaminomethylation of other π-unsaturated reactants, such as 1,3-dienes, has not been reported, as regioselectivity and “over-hydroformylation” to form dialdehydes are difficult to control (Scheme 1, eqn (2)).^11^ In connection with our exploration of hydrogenation and transfer hydrogenation in the context of reductive C–C coupling, we have found that paraformaldehyde serves as a convenient and inexpensive C1-building block for the hydrohydroxymethylation of 1,3-dienes,^12^ allenes^13^,^14^ and alkynes.^15^ Most importantly, reductive couplings of paraformaldehyde provide access to products of hydrohydroxymethylation that cannot be formed selectively under hydroformylation conditions.^16^

These results supported the feasibly of corresponding hydroaminomethylations wherein π-unsaturated reactants are reductively coupled with formaldimines. In proof-of-concept studies, it was found that 1,1-disubstituted allenes engage in regioselective reductive coupling with formaldimines derived *in situ* through cracking of 1,3,5-tris(aryl)-hexahydro-1,3,5-triazines under the conditions of ruthenium catalyzed transfer hydrogenation employing 2-propanol as terminal reductant.^17^ Corresponding hydroaminomethylations of 1,3-dienes such as butadiene, isoprene and myrcene, which are important feedstock chemicals, would be even more desirable, however, competing aza-Diels–Alder cycloaddition^18^ and alkene isomerization^19^ of the homoallylic amines products rendered the outcome of such processes uncertain. Here, we report that ruthenium complexes modified by dCypm (bis(dicyclohexylphosphino)methane) catalyze the 2-propanol mediated reductive coupling of 2-substituted 1,3-dienes with 1,3,5-tris(aryl)-hexahydro-1,3,5-triazines to form products of hydroaminomethylation as single regioisomers with complete suppression of olefin isomerization in all but one case (Scheme 1, eqn (3)). These transformations represent the first examples of diene hydroaminomethylation.^20^,^21^

## Results and discussion

Hexahydro-1,3,5-triazine **2a** is a white, crystalline solid conveniently prepared through the condensation of paraformaldehyde and *para*-anisidine.^22^ In an initial experiment, butadiene **1a** was exposed to hexahydro-1,3,5-triazine **2a** in the presence of 2-propanol (400 mol%) and commercial HClRu(CO)(PPh_3_)_3_ (5 mol%) in toluene solvent (0.5 M) at 120 °C. The targeted product of hydroaminomethylation **3a** was obtained as a single isomer in 11% yield (Table 1, entry 1). A series of phosphine ligands were evaluated for their ability to enhance conversion. The isolated yield of **3a** was not improved upon use of the monodentate phosphine ligands, for example PCy_3_ (Table 1, entry 2). A series of chelating bis(diphenylphosphino)-substituted ligands were screened, including dppf, dppm and dppe (Table 1, entries 3–5). The isolated yield of **3a** was increased to 81% using dppe, however, substantial quantities of olefin isomerization product, allylic amine *iso*-**3a**, was formed (Table 1, entry 5).^19^ The chelating ligands dCypm and dCype, which incorporate bis(dicyclohexylphosphino) moieties, provided superior results, delivering the homoallylic amine **3a** in 86% (10 : 1, **3a** : *iso*-**3a**) and 71% (20 : 1, **3a** : *iso*-**3a**) isolated yields, respectively (Table 1, entries 6 and 7). Attempts to enhance the performance of the dCypm-modified catalyst through variation of reaction temperature (Table 1, entries 8 and 9) or reaction time (Table 1, entry 10) did not avail additional improvement (Table 2).

**Table 1:** Selected optimization experiments in the ruthenium catalyzed hydroaminomethylation of butadiene **1a***via* 2-propanol mediated transfer hydrogenation^*a*^

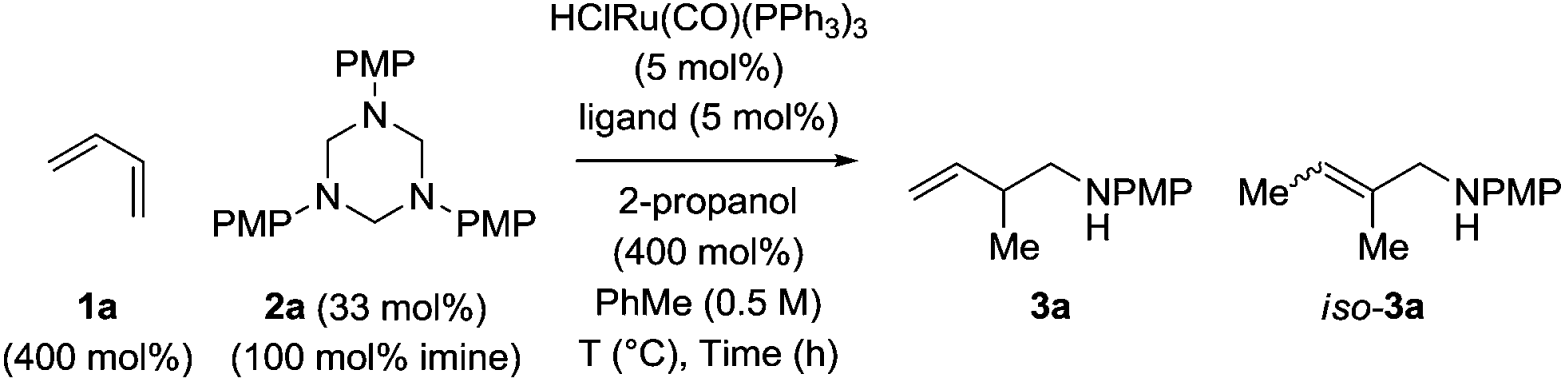					
Entry	Ligand	*T* (°C)	Time (h)	Yield **3a**	**3a** : *iso*-**3a**
1	—	120	24	11%	>20 : 1
2^*b*^	PCy_3_	120	24	10%	>20 : 1
3	dppf	120	24	19%	>20 : 1
4	dppm	120	24	34%	4 : 1
5	dppe	120	24	81%	4 : 1
**6**	**dCypm**	**120**	**24**	**86%**	**10 : 1**
7	dCype	120	24	71%	20 : 1
8	dCypm	110	24	65%	10 : 1
9	dCypm	140	24	82%	8 : 1
10	dCypm	120	12	74%	10 : 1

^*a*^Yields are of material isolated by silica gel chromatography. Isomeric ratios were determined *via*^1^H NMR analysis. PMP = *para*-methoxyphenyl, dppf (1,1′-bis(diphenylphosphino)ferrocene), dppm (bis(diphenylphosphino)methane), dppe (1,2-bis(diphenylphosphino)ethane), dCypm (bis(dicyclohexylphosphino)methane), dCype (1,2-bis(dicyclohexylphosphino)ethane).

^*b*^PCy_3_ (10 mol%). See ESI† for further details.

**Table 2:** Ruthenium catalyzed hydroaminomethylation of 1,3-dienes **1a–1i** to form homoallylic amines **3a–3i***via* 2-propanol mediated transfer hydrogenation^*a*^

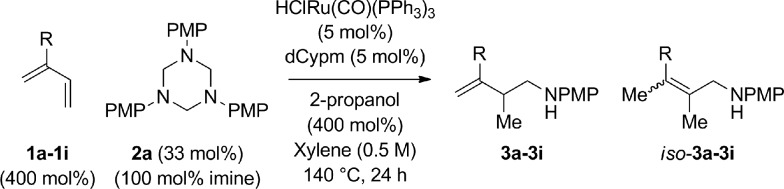			
Entry	1,3-Diene	Product	Yield (**3** : *iso*-**3**)
1^*b*^	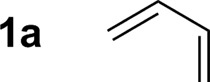	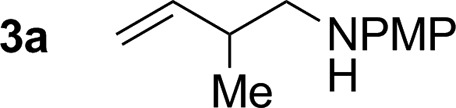	86% yield 10 : 1 (**3a** : *iso*-**3a**)
2	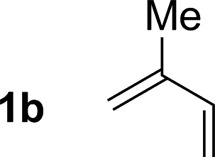	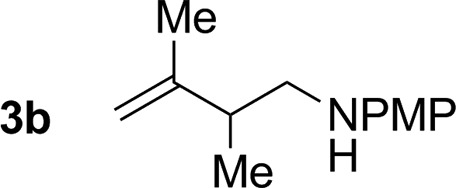	79% yield >20 : 1 (**3b** : *iso*-**3b**)
3	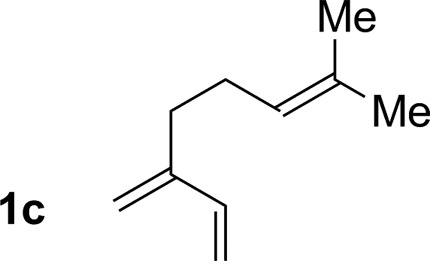	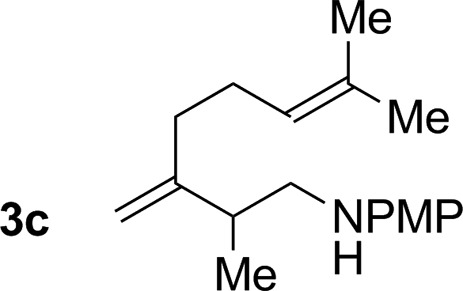	76% yield >20 : 1 (**3c** : *iso*-**3c**)
4	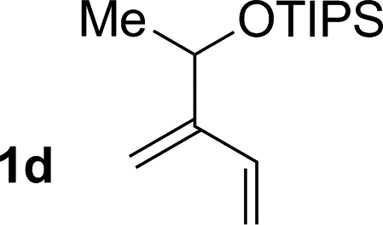	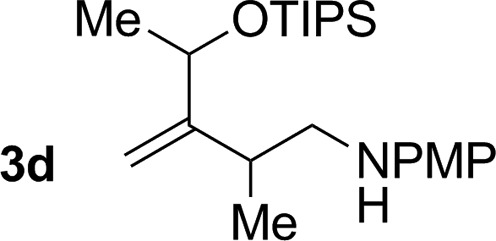	71% yield, 2 : 1 dr >20 : 1 (**3d** : *iso*-**3d**)
5	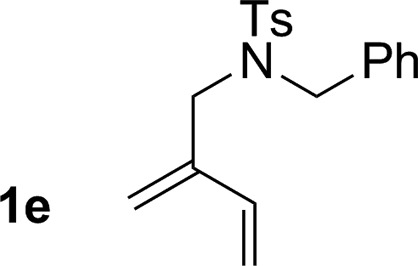	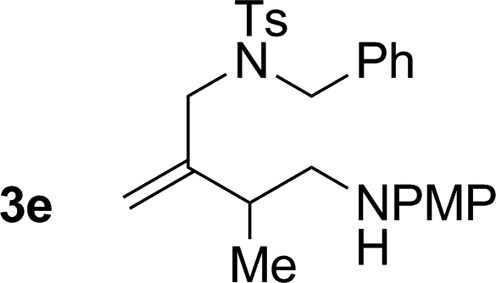	74% yield >20 : 1 (**3e** : *iso*-**3e**)
6	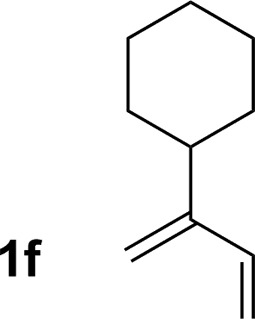	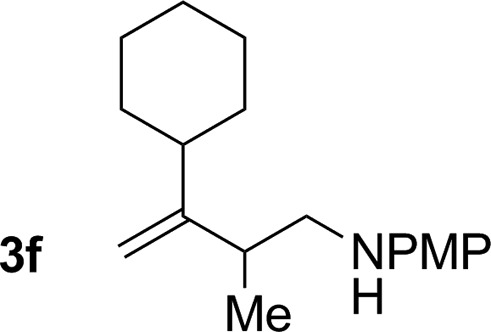	70% yield >20 : 1 (**3f** : *iso*-**3f**)
7	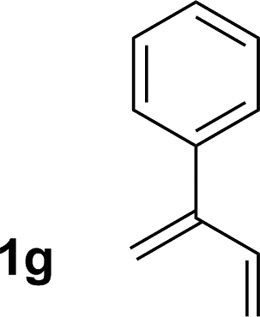	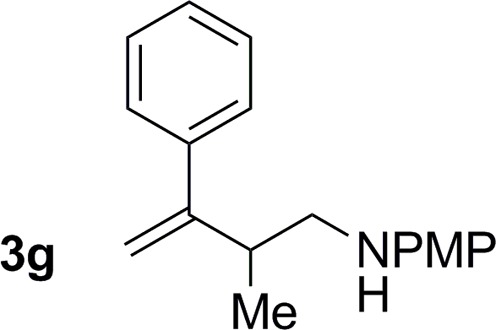	77% yield >20 : 1 (**3g** : *iso*-**3g**)
8	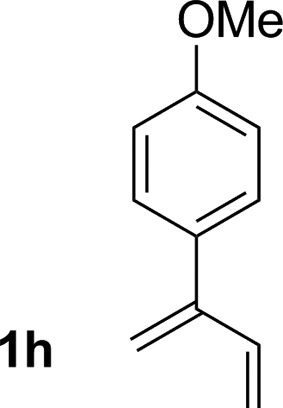	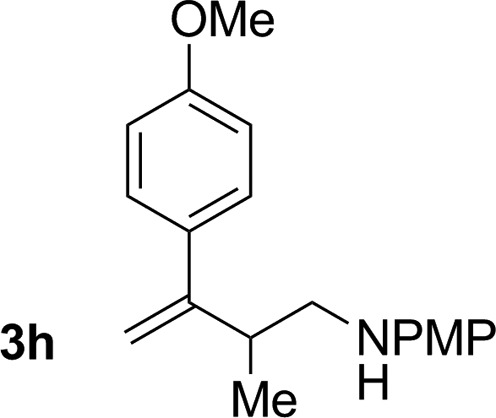	72% yield >20 : 1 (**3h** : *iso*-**3h**)
9	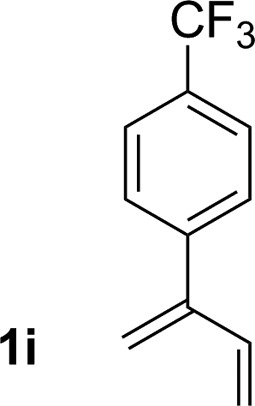	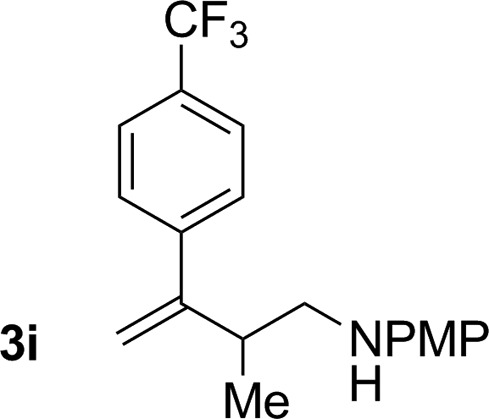	81% yield >20 : 1 (**3i** : *iso*-**3i**)

^*a*^Yields are of material isolated by silica gel chromatography. Isomeric ratios were determined *via*^1^H NMR analysis.

^*b*^PhMe (0.5 M), 120 °C. See ESI† for further experimental details.

**Scheme 2:**
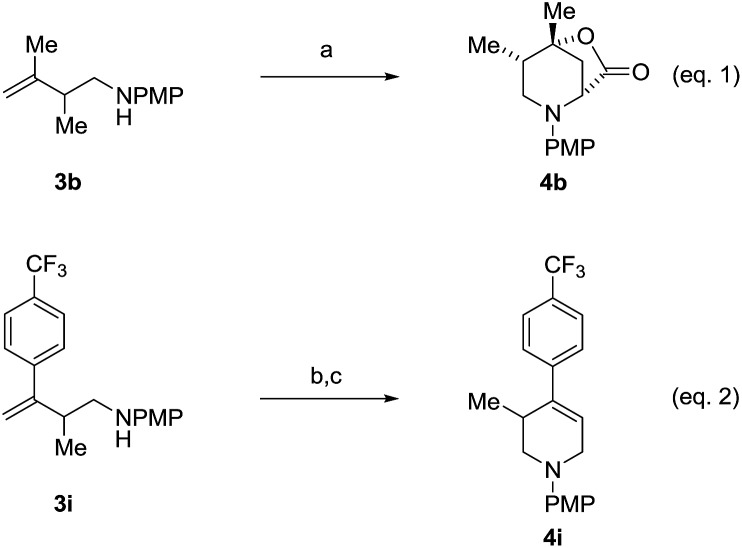
Conversion of hydroaminomethylation products **3b** and **3i** to compounds **4b** and **4i**, respectively. ^a^Yields are of material isolated by silica gel chromatography. (a) (HO)_2_CCO_2_H, MeCN–H_2_O, 25 °C, 80% yield, 10 : 1 dr (b) BrCH_2_CH=CH_2_, K_2_CO_3_, DMF, 25 °C, 75% yield. (c) Grubbs-II, DCM, 40 °C, 72% yield. See ESI† for further experimental details.

An attempt was made to apply these optimal conditions (Table, entry 6) to a series of 2-substituted 1,3-dienes **1b–1i**, however, at 120 °C the desired products **3b–3i** were accompanied by significant quantities of the corresponding aza-Diels–Alder [4 + 2] cycloadducts.^18^ 2-Substituted dienes display an enhanced conformational preference for the s-*cis* conformer, which increases their rate of Diels–Alder cycloaddition relative to butadiene. At slightly higher temperatures (140 °C in xylene solvent), competing Diels–Alder reaction decelerates with respect to hydroaminomethylation and could be completely suppressed. Using these slightly modified conditions, 2-substituted 1,3-dienes **1b–1i** were reacted with hexahydro-1,3,5-triazine **2a** to furnish the products of hydroaminomethylation **3b–3i** in good yield as single regioisomers, and isomeric allylic amines **iso-3b–3i** were not observed. Notably, a range of substituents are tolerated at the 2-position of the diene, including branched aliphatic moieties (**1d**, **1f**), groups with allylic heteroatoms (**1d**, **1e**) and aryl groups (**1g–1i**). Under the present conditions, 1-substituted dienes engage in reductive coupling, however, lower conversions and selectivities were observed. To illustrate the utility of homoallylic amines **3a–3i**, the hydroaminomethylation product **3b** was transformed into the trisubstituted piperidine **4b***via* Prins reaction with glyoxylic acid mono-hydrate (Scheme 2, eqn (1)).^23^ Additionally, adduct **3i** was subjected to *N*-allylation and ring closing metathesis to form the disubstituted piperidine **4i** (Scheme 2, eqn (2)).^24^

Variation of the 1,3,5-tris(aryl)-hexahydro-1,3,5-triazine was subsequently investigated in the hydroaminomethylation of butadiene **1a** (Table 3). *N*-Aryl substituted triazines **2a–2f** were subjected to optimal conditions identified for the hydroaminomethylation of butadiene using triazine **2a** (Table 1, entry 6). Electron rich *N*-aryl triazines **2a–2c**, including *ortho*-substituted triazine **2c**, undergo hydroaminomethylation efficiently to afford the branched homoallylic amines **3a–5a** with complete regiocontrol. In each case, small quantities of the allylic amines *iso-***3a–5a** were observed as side-products. Electron neutral triazine **2d** and electron deficient triazines **2e** and **2f** were converted to the respective homoallylic amines **6a**, **7a** and **8a** in good yield, although increased quantities of the allylic amine side-products were observed. As illustrated in the formation of **8a**, nitrogen bearing heterocycles are tolerated. Attempted use of *N*-alkyl, *N*-acyl and *N*-sulfonyl triazines failed in the coupling with dienes under these initially developed conditions. It should be noted that the **3a–8a** : *iso-***3a–8a** ratio does not change as a function of conversion or reaction time, suggesting olefin isomerization is kinetically controlled, perhaps occurring by way of the homoallylic amidoruthenium intermediate (*vida supra*).

**Table 3:** Ruthenium catalyzed hydroaminomethylation of butadiene **1a** with 1,3,5-tris(aryl)-hexahydro-1,3,5-triazines **2a–2f** to form homoallylic amines **3a–8a**^*a*^

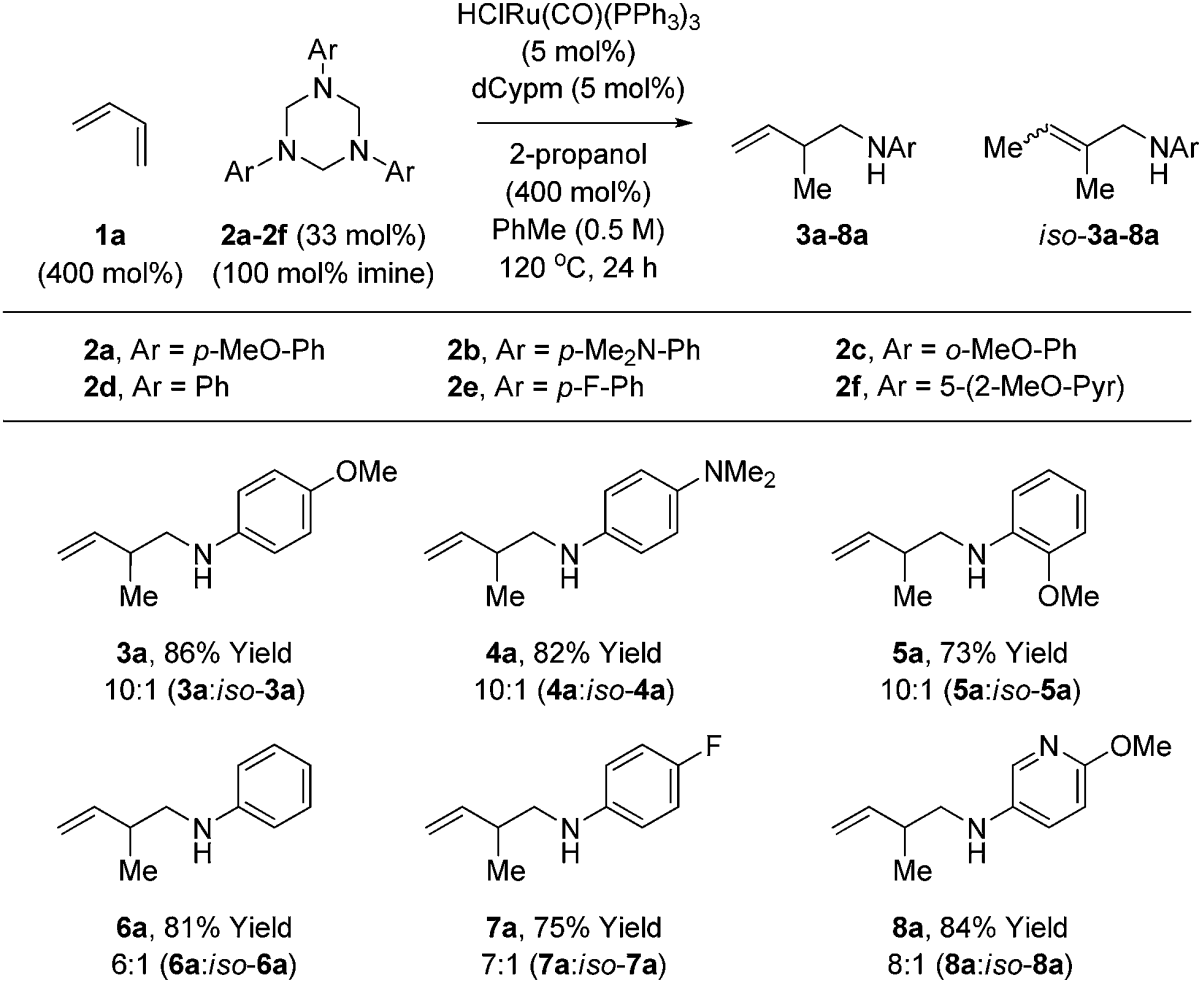

^*a*^Yields are of material isolated by silica gel chromatography. Isomeric ratios were determined *via*^1^H NMR analysis. See ESI† for further experimental details.

**Scheme 3:**
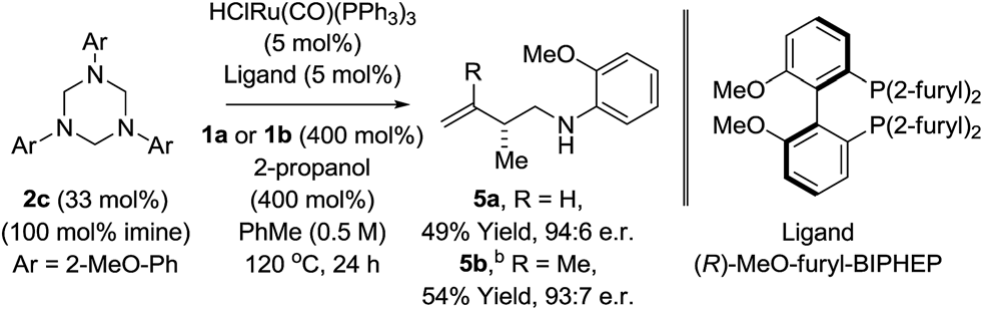
Enantioselective ruthenium catalyzed hydroaminomethylation of butadiene **1a** and isoprene **1b**. ^a^Yields are of material isolated by silica gel chromatography. Enantiomeric ratios were determined by chiral stationary phase HPLC analysis. ^b^Xylene (0.5 M), 140 °C. See ESI† for further experimental details.

Having established favourable conditions for diene hydroaminomethylation, enantioselective variants were investigated in reactions of butadiene **1a**. A survey of triazines **2a–2f** revealed that triazine **2c** derived from *ortho*-anisidine provided the highest levels of enantiomeric enrichment. Among various chiral phosphine ligands, (*R*)-MeO-furyl-BIPHEP provided the highest levels of enantiocontrol. In the presence of this chiral ligand, the reaction of 1,3-butadiene **1a** with *N-ortho*-methoxyphenyl triazine **2c** delivered the homoallylic amine **5a** in 49% yield as a 94 : 6 ratio of enantiomers in the absence of allylic amine side-product *iso-***5a** (Scheme 3). Application of these initially developed conditions for enantioselective hydroaminomethylation to isoprene resulted in the formation of homoallylic amine **5b** in 54% yield as a 93 : 7 ratio of enantiomers (Scheme 3). The absolute stereochemical assignment of **5a** was determined by single crystal X-ray diffraction analysis of the corresponding 4-nitrobenzenesulfonamide.

## Mechanistic studies

**Scheme 4:**
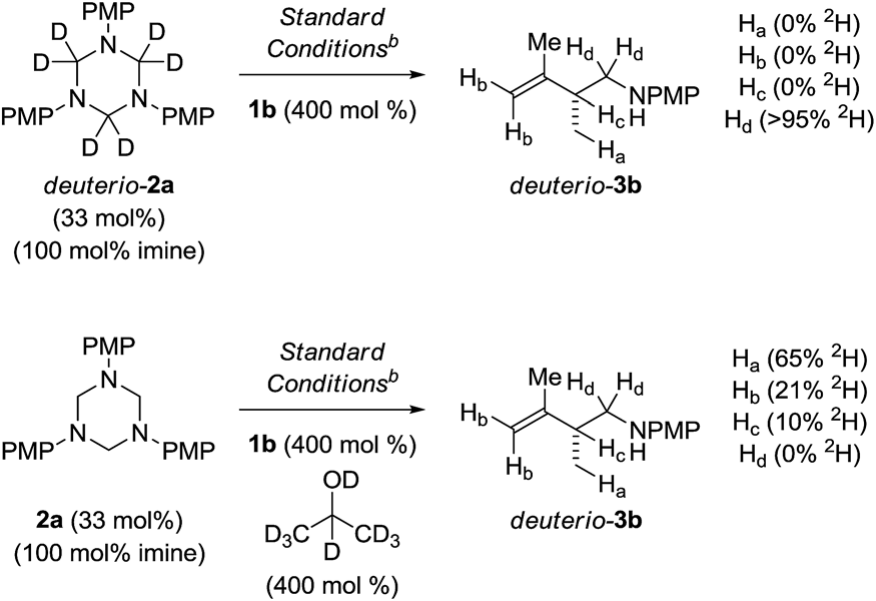
Deuterium labelling studies of the ruthenium catalyzed hydroaminomethylation of isoprene **1b**. ^a^Yields are of material isolated by silica gel chromatography. ^b^Xylene (0.5 M), 140 °C. See ESI† for further experimental details.

To illuminate key features of the catalytic mechanism, deuterium labelling studies of the ruthenium catalyzed hydroaminomethylation of isoprene **1b** were performed (Scheme 4). Hydroaminomethylation of isoprene **1b** using the deuterated triazine deuterio-**2a** provided deuterio-**3b** with complete retention of deuterium at the methylene carbon bearing nitrogen (>95% ^2^H). Deuterium was not detected at any other position. This experiment suggests deuterio-**3b** is inert with respect to amine dehydrogenation under these conditions. In a second experiment, isoprene **1b** was subjected to hydroaminomethylation using triazine **2a** in the presence of *d*_8_-2-propanol. As anticipated, the product deuterio-**3b** incorporates significant quantities of deuterium at the methyl group (65% ^2^H). However, deuterium also is incorporated at the terminal vinylic positions (21% ^2^H) and, to a lesser extent, the allylic position (10% ^2^H). These data corroborate a scenario wherein rapid, reversible and non-regioselective diene hydrometalation occurs in advance of turn-over limiting imine addition. Reversible hydrometalation accounts for incomplete deuterium incorporation. Adventitious water also may diminish the extent of deuterium incorporation.^25^

**Scheme 5:**
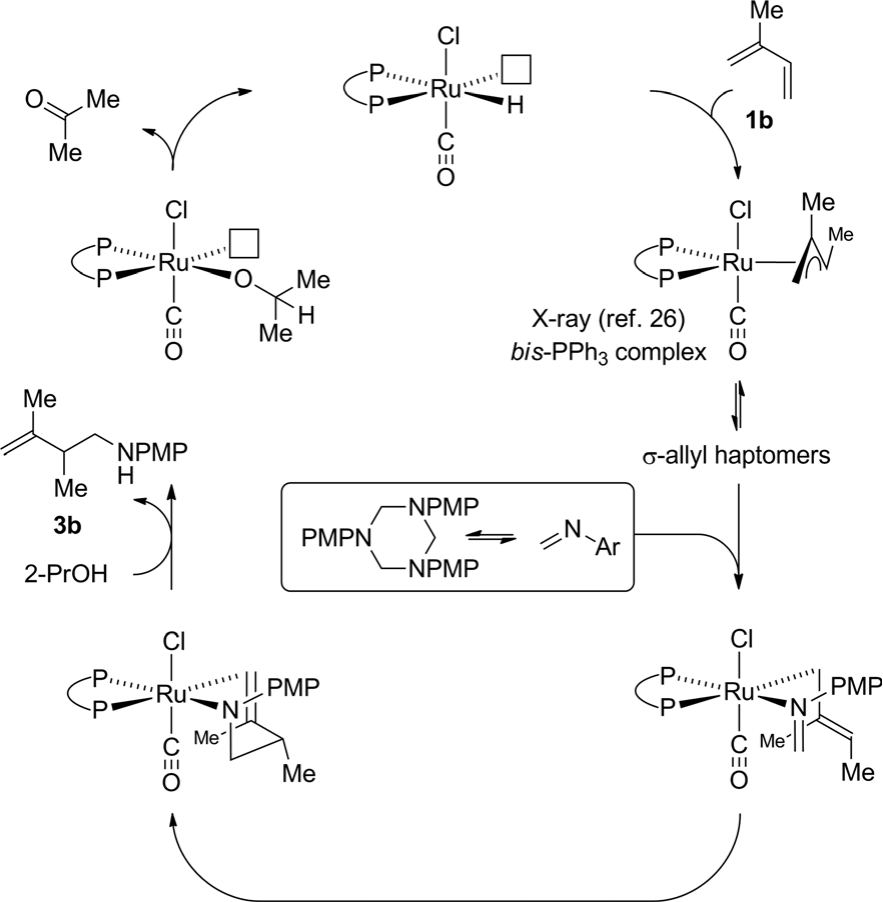
General mechanism for ruthenium catalyzed diene hydroaminomethylation *via* transfer hydrogenation.

Guided by these data, a mechanism for ruthenium catalyzed diene hydroaminomethylation *via* transfer hydrogenation was proposed (Scheme 5). Diene hydroruthenation delivers a nucleophilic allylruthenium complex. The stoichiometric reaction of HXRu(CO)(PPh_3_)_3_ (X = Cl, Br) with dienes (or allenes) to form π-allylruthenium complexes has been reported.^26^ In the case of isoprene **1b**,26a*cis*-stereochemistry between the methyl groups of the resulting π-allyl are observed. Notably, HClRu(CO)(PPh_3_)_3_ hydrometalates 1,1-dimethylallene to initially form a 1,1-dimethyl substituted π-allylruthenium complex that rearranges to the *cis*-1,2-dimethyl substituted π-allylruthenium complex,26*c* suggesting *cis*-stereochemistry represents a thermodynamic rather than kinetic preference. Intervention of a single geometrical isomer at the stage of the σ-allylruthenium intermediate and ensuing transition state for imine addition appears consistent with the relatively high levels of enantioselectivity observed in the asymmetric hydroaminomethylation of isoprene (Scheme 3). Protonolytic cleavage of the amidoruthenium complex derived upon imine addition mediated by isopropanol releases the product of hydroaminomethylation **3b** and regenerates the ruthenium hydride to close the catalytic cycle.

## Conclusion

In summary, using the concepts of redox-triggered C=X (X = O, N) addition,^27^ we report the first examples of diene hydroaminomethylation, including asymmetric variants. Specifically, ruthenium catalyzed transfer hydrogenation of 1,3-dienes in the presence of tris(aryl)-hexahydro-1,3,5-triazines results in diene–formaldimine reductive coupling to deliver homoallylic amines in good yield with complete levels of regioselectivity. While further optimization is required to enhance performance, these processes define an alternative to classical carbonylative hydroaminomethylation *via* hydroformylation-reductive amination, which is presently limited to reactions of non-conjugated olefins. More broadly, these studies contribute to an ever-growing body of catalytic C–C bond formations that merge the characteristics of carbonyl and imine addition with transfer hydrogenation.^27^

## Supplementary Material

Supplementary information

Crystal structure data
