# Supplementary material for: Diene hydroaminomethylation via ruthenium-catalyzed C–C bond forming transfer hydrogenation: beyond carbonylation
Source: Chem Sci. 2015 Nov 17;7(1):136–41. doi: 10.1039/c5sc03854e (PMC5950558; doi:10.1039/c5sc03854e)

Electronic Supplementary Material (ESI) for Chemical Science.
This journal is © The Royal Society of Chemistry 2015

Supplementary Information

Diene Hydroaminomethylation *via* Ruthenium-Catalyzed C-C Bond Forming Transfer Hydrogenation: Beyond Carbonylation

Susumu Oda, Jana Franke and Michael J. Krische*

University of Texas at Austin, Department of Chemistry, Austin, TX 78712,

Table of Contents

I.	General Experimental Details	S2
II.	Experimental Procedure and Spectral Data Adducts **3a-3i**	S3
III.	Experimental Procedure and Spectral Data Adducts **4a-8a**	S32
IV.	Experimental Procedure and Spectral Data for the Elaboration of Product **3i** and **3b**	S49
V.	Experimental Procedure and Spectral Data Adducts *deuterio*-**3b**	S60
VI.	Experimental Procedure and Spectral Data Adducts (*R*)-**5a** and (*R*)-**5b**	S66
VII.	Experimental Procedure and Crystallographic Material for **S1**	S72

I. General Experimental Details.

All reactions were run under an atmosphere of argon, unless otherwise indicated. Anhydrous solvents were transferred *via* oven-dried syringes. Reaction tubes and flasks were oven-dried and cooled under a stream of argon. Reaction tubes were purchased from Fischer Scientific (catalog number 14-959-35C). Toluene (PhMe) was distilled from sodium and benzophenone. Xylene was distilled from CaH_2. $RuHCl(CO)(PPh_3)_3$ was prepared according to literature procedure.[1] 2-Propanol (99.8%, extra dry) was obtained from Acros Organics. Dienes **1a**-**1c** were obtained from Sigma-Aldrich. Dienes **1d**-**1l** were prepared according to literature procedure.[2] Triazines **2a**-**2f** were prepared according to literature procedure.[3] Bis(dicyclohexylphosphino)methane (dCypm) was obtained from Sigma-Aldrich and used as received. Analytical thin-layer chromatography (TLC) was carried out using 0.25 mm commercial silica gel plates (Dynamic Adsorbents F_{254}) and products were visualized by UV, $KMnO_4$, *p*-anisaldehyde (PAA) and/or Magic Seebach stain. Preparative column chromatography employing Silicycle silica gel (40-63 μm) was performed according to the method of Still.[4] Infrared spectra were recorded on a Perkin-Elmer 1600 spectrometer. Low-resolution mass spectra (LRMS) were obtained on a Karatos MS9 and are reported as m/z (relative intensity). Accurate masses are reported for the molecular ion $[M+H]^+$ or a suitable fragment ion. Proton nuclear magnetic resonance (^{1}H NMR) spectra were recorded with a Varian Gemini (400 MHz) spectrometer. Chemical shifts are reported in delta (δ) units, parts per million (ppm) downfield from tetramethylsilane or ppm relative to the center of the singlet at 7.26 ppm for deuterochloroform. Coupling constants are reported in Hertz (Hz). Carbon-13 nuclear magnetic resonance (^{13}C NMR) spectra were recorded with a Varian Gemini 400 (100 MHz) spectrometer. Chemical shifts are reported in delta (δ) units, ppm relative to the center of the triplet at 77.0 ppm for deuterochloroform. ^{13}C NMR spectra were routinely run with broadband decoupling. Fluorine-19 nuclear magnetic resonance (^{19}F NMR) spectra were recorded with a Varian Gemini 400 (100 MHz) spectrometer. Deuterium nuclear magnetic resonance (^{2}H NMR) spectra were recorded in $CHCl_3$ solution with a Varian Gemini 500 (77 MHz) spectrometer (relaxation delay 2.00 s).

[1]T. Joseph, S. S. Deshpande, S. B. Halligudi, A. Vinu, S. Ernst, M. Hartmann, *J. Mol. Catal. A.* **2003**, *206*, 13–21.
[2]T. Smejkal, H. Han, B. Breit, M. J. Krische, *J. Am. Chem. Soc.* **2009**, *131*, 10366–10367.
[3]A. G. Giumanini, G. Verardo, E. Zangrando, L. Lassiani, *J. Prakt. Chem*. **1987**, *329*, 1087–1103.
[4]W. C. Still, M. Kahn, A. Mitra, *J. Org. Chem.* **1978**, *43*, 2923–2925.

II. Experimental Procedures and Spectral Data Adducts 3a-3i

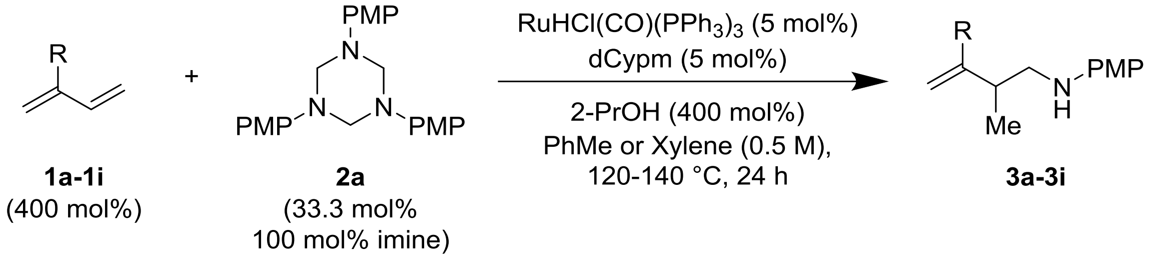

General Procedure A for the coupling of triazine 2a to diene 1a

To an oven-dried pressure tube equipped with magnetic stir bar was added $RuHCl(CO)(PPh_3)_3$ (9.5 mg, 0.010 mmol, 5 mol%), bis(dicyclohexylphosphino)methane (dCypm) (4.1 mg, 0.010 mmol, 5 mol%), and triazine **2a** (27.0 mg, 0.067 mmol, 33.3 mol% (0.200 mmol, 100 mol% of imine)). The tube was sealed with a rubber septum, purged with argon, and toluene (0.4 mL, 0.5 M with respect to formimine), diene **1a** (0.800 mmol, 400 mol%), and isopropanol (61 µL, 0.800 mmol, 400 mol%) were added. The rubber septum was quickly replaced with a screw cap and the reaction was heated to 120 °C for 24 hours. The reaction mixture was allowed to cool to room temperature, concentrated *in vacuo*, and purified by flash column chromatography (SiO_2) to furnish the title compounds.

General Procedure B for the coupling of triazine 2a to dienes 1b-1i

To an oven-dried pressure tube equipped with magnetic stir bar was added $RuHCl(CO)(PPh_3)_3$ (9.5 mg, 0.010 mmol, 5 mol%), bis(dicyclohexylphosphino)methane (dCypm) (4.1 mg, 0.010 mmol, 5 mol%), and triazine **2a** (27.0 mg, 0.067 mmol, 33.3 mol% (0.200 mmol, 100 mol% of imine)). The tube was sealed with a rubber septum, purged with argon, and xylene (0.4 mL, 0.5 M with respect to formimine), diene (0.800 mmol, 400 mol%), and isopropanol (61 µL, 0.800 mmol, 400 mol%) were added. The rubber septum was quickly replaced with a screw cap and the reaction was heated to 140 °C for 24 hours. The reaction mixture was allowed to cool to room temperature, concentrated *in vacuo*, and purified by flash column chromatography (SiO_2) to furnish the title compounds.

***N*-(2-methylbut-3-en-1-yl)-4-methoxyaniline (3a)**

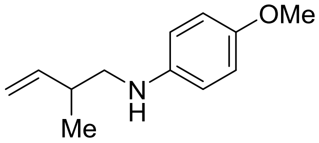

The reaction was conducted in accordance with **General Procedure A** (*via* diene **1a**). After heating the reaction for 24 hours, the mixture was concentrated *in vacuo* and purified by flash column chromatography (SiO_2, 6% EtOAc/hexanes) to furnish the title compound (32.8 mg, 86%) as a yellow oil.

^{1}H NMR (400 MHz, $CDCl_3$): δ 6.79 (d, *J* = 9.0 Hz, 2H), 6.59 (d, *J* = 9.0 Hz, 2H), 5.72 (ddd, *J* = 17.2, 10.3, 7.8 Hz, 1H), 5.10 (dddd, *J* = 10.2, 6.8, 1.8, 1.0 Hz, 2H), 3.75 (s, 3H), 3.45 (br, 1H), 3.06 (dd, *J* = 11.8, 5.5 Hz, 1H), 2.90 (dd, *J* = 11.8, 8.3 Hz, 1H), 2.54 – 2.40 (m, 1H), 1.10 (d, J = 6.8 Hz, 3H).

^{13}C NMR (100 MHz, $CDCl_3$): δ 152.15, 142.73, 142.18, 115.26, 115.01, 114.36, 55.95, 50.43, 37.95, 18.20.

LRMS (ESI): m/z 192 $[M+H]^+$

FTIR (neat): 2955, 2830, 1510, 1463, 1374, 1295, 1231, 1178, 1123, 1036, 914, 816, 754 cm^{-1}.

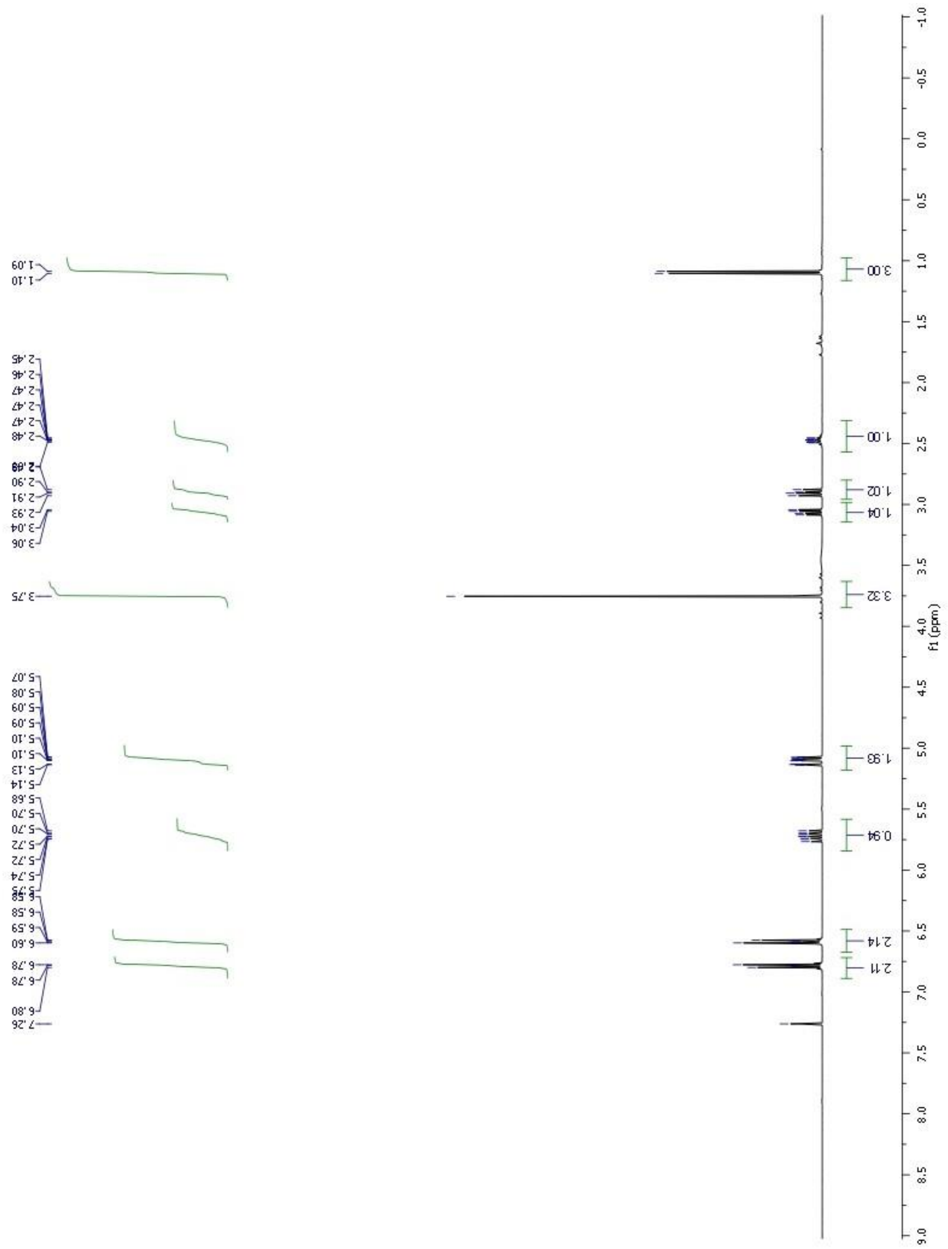

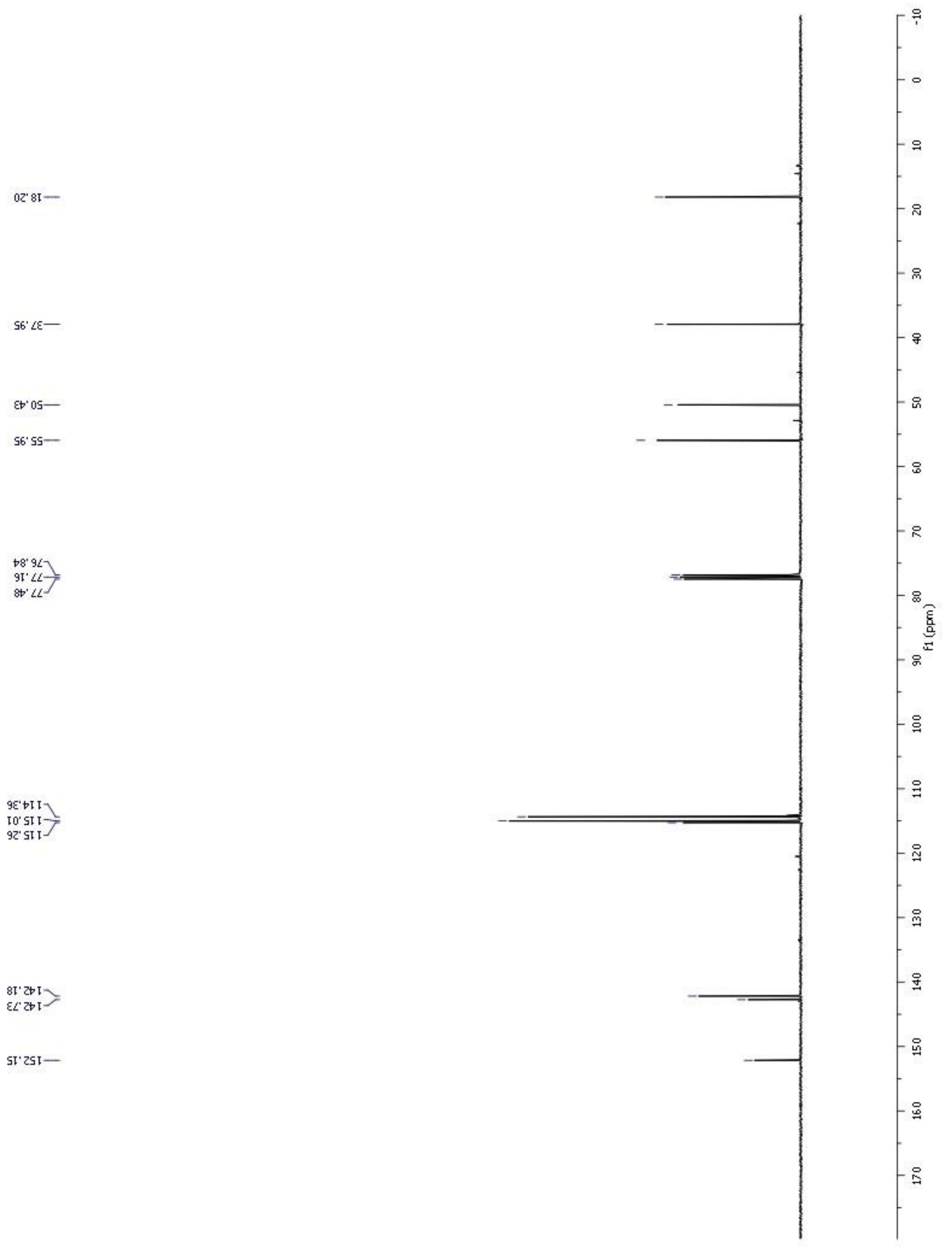
18.20
37.95
50.43
55.95
76.84
77.16
77.48
114.36
115.01
115.26
142.18
142.73
152.15
f1 (ppm)

***N*-(2,3-dimethylbut-3-en-1-yl)-4-methoxyaniline (3b)**

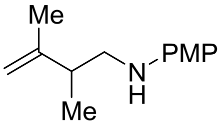

The reaction was conducted in accordance with **General Procedure B** (*via* diene **1b**). After heating the reaction for 24 hours, the mixture was concentrated *in vacuo* and purified by flash column chromatography (SiO_2, 5% EtOAc/hexanes) to furnish the title compound (32.4 mg, 79%) as a yellow oil.

^{1}H NMR (400 MHz, $CDCl_3$): δ 6.78 (d, *J* = 8.9 Hz, 2H), 6.58 (d, *J* = 8.9 Hz, 2H), 4.98 – 4.67 (m, 2H), 3.75 (s, 3H), 3.37 (br, 1H), 2.99 (ddd, *J* = 20.1, 11.6, 7.1 Hz, 2H), 2.50 (dq, *J* = 13.6, 7.0 Hz, 1H), 1.69 (s, 3H), 1.10 (d, *J* = 6.9 Hz, 3H).

^{13}C NMR (100 MHz, $CDCl_3$): δ 152.10, 147.63, 142.82, 115.02, 114.23, 111.87, 55.98, 48.60, 40.97, 18.74, 17.66.

LRMS (CI): m/z 206 $[M+H]^+$

FTIR (neat): 3396, 2961, 2830, 1643, 1510, 1479, 1463, 1407, 1375, 1294, 1232, 1179, 1119, 1036, 891, 816, 732 cm^{-1}.

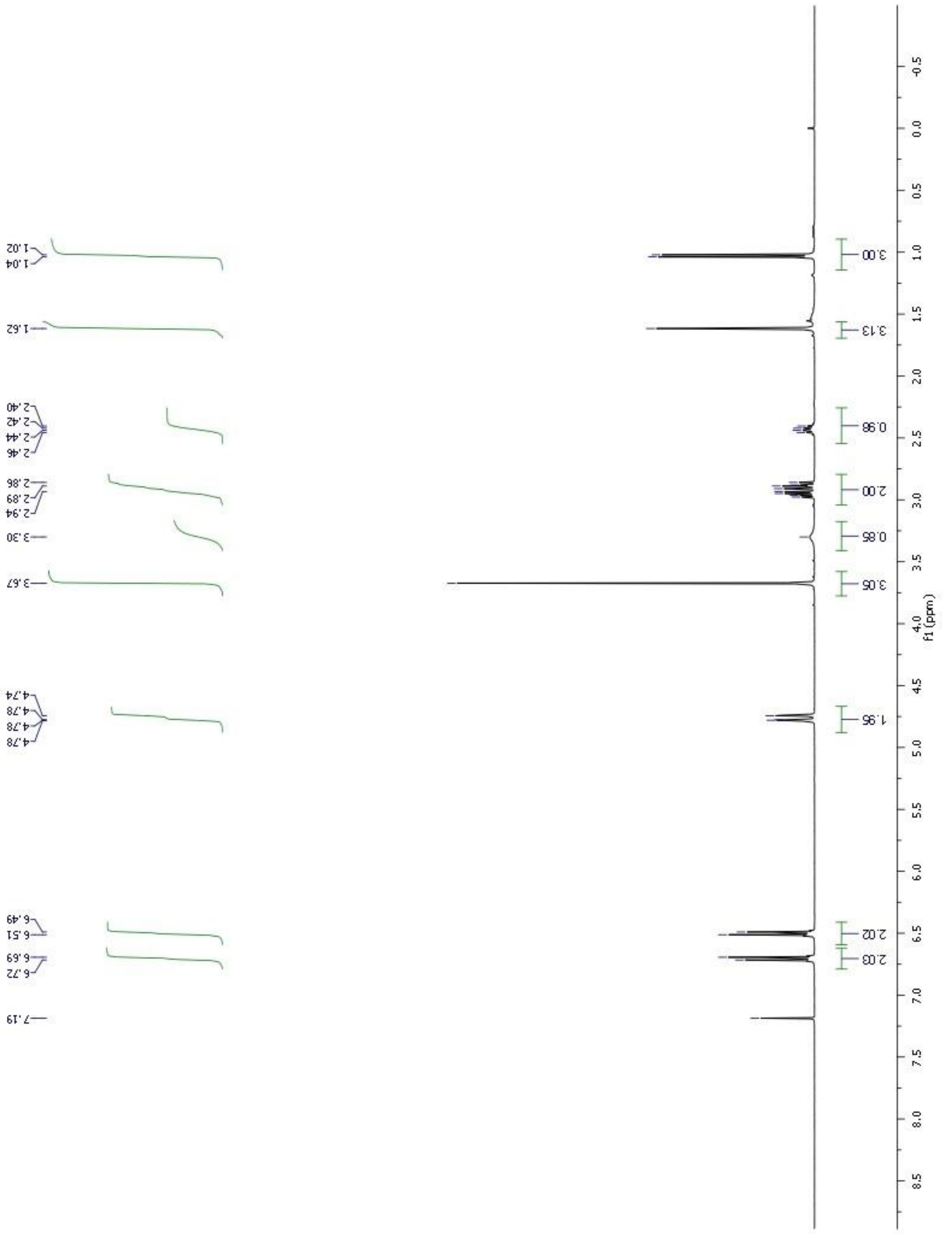
1.02
1.04
1.62
2.40
2.42
2.44
2.46
2.86
2.89
2.94
3.30
3.67
4.74
4.78
4.78
4.78
6.49
6.51
6.69
6.72
7.19
3.00
3.13
0.98
2.00
0.85
3.05
1.96
2.02
2.03
f1 (ppm)

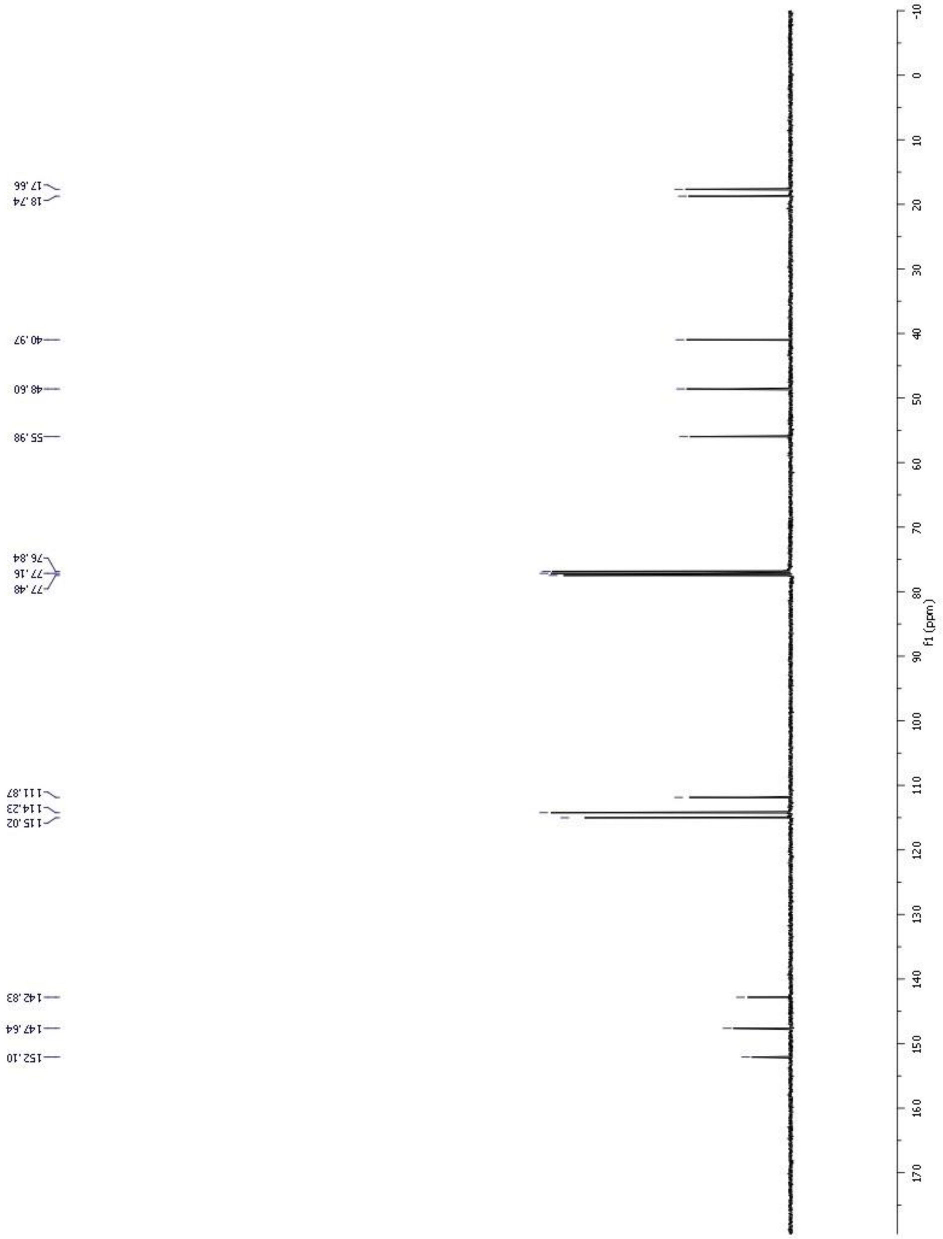
17.66
18.74
40.97
48.60
55.98
76.84
77.16
77.48
111.87
114.23
115.02
142.83
147.64
152.10
f1 (ppm)

N-(2,7-dimethyl-3-methylene-6-octen-1-yl)-4-methoxyaniline (3c)

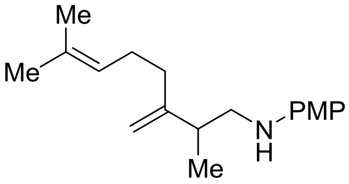

The reaction was conducted in accordance with **General Procedure B** (*via* diene **1c**). After heating the reaction for 24 hours, the mixture was concentrated *in vacuo* and purified by flash column chromatography (SiO_2, 5% EtOAc/hexanes) to furnish the title compound (41.5mg, 76%) as a yellow oil.

^{1}H NMR (400 MHz, $CDCl_3$): δ 6.77 (d, *J* = 8.8 Hz, 2H), 6.57 (d, *J* = 8.8 Hz, 2H), 5.11 (t, *J* = 6.2 Hz, 1H), 4.88 (d, *J* = 6.5 Hz, 2H), 3.75 (s, 3H), 3.40 (br, 1H), 3.03 (d, *J* = 7.0 Hz, 2H), 2.54 – 2.43 (m, 1H), 2.14 (dd, *J* = 14.5, 7.1 Hz, 2H), 2.02 (dd, *J* = 13.9, 6.5 Hz, 2H), 1.68 (s, 3H), 1.60 (s, 3H), 1.11 (d, *J* = 6.9 Hz, 3H).

^{13}C NMR (100 MHz, $CDCl_3$): δ 152.09, 151.77, 142.84, 131.97, 124.17, 115.03, 114.26, 109.93, 55.99, 49.15, 40.01, 33.28, 26.68, 25.85, 18.24, 17.89.

LRMS (ESI): m/z 274 $[M+H]^+$

FTIR (neat): 3398, 2969, 2925, 2360, 1783, 1639, 1510, 1440, 1374, 1232, 1217, 1179, 1116, 1038, 891, 816, 753 cm^{-1}.

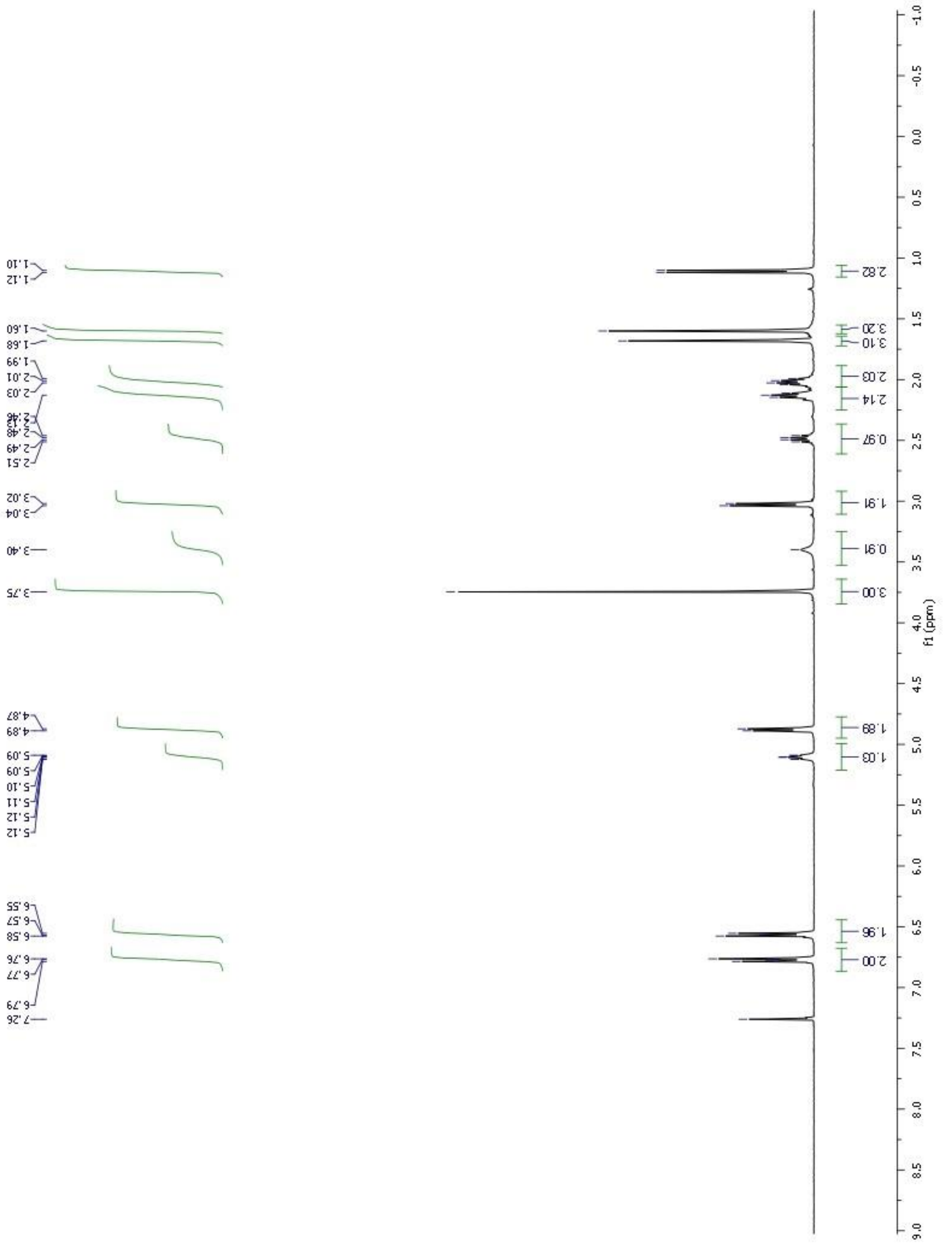

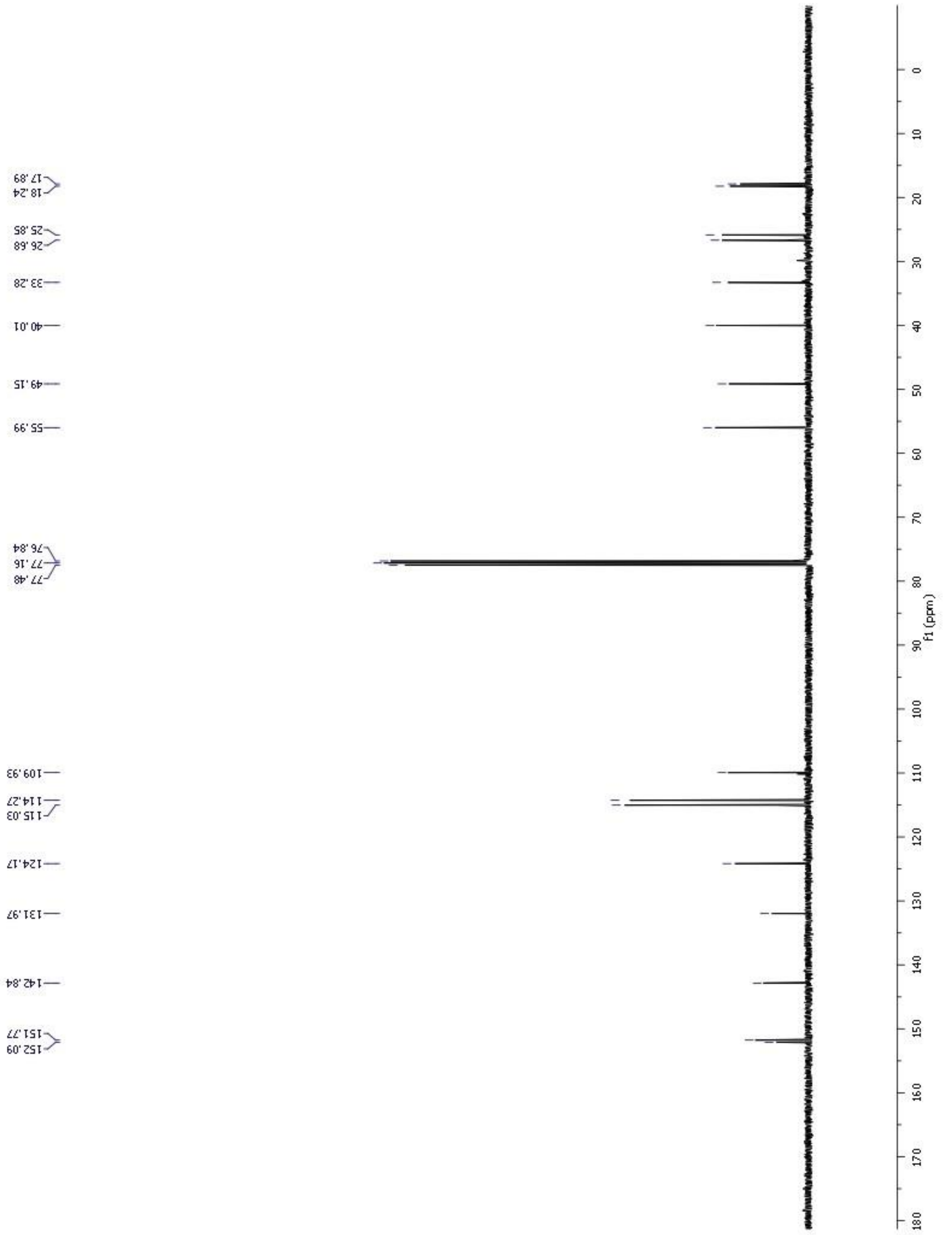
17.89
18.24
25.85
26.68
33.28
40.01
49.15
55.99
76.84
77.16
77.48
109.93
114.27
115.03
124.17
131.97
142.84
151.77
152.09
f1 (ppm)

***N*-(2-methyl-3-methylene-4-(triisopropylsioxy)pentyl)-4-methoxyaniline (3d)**

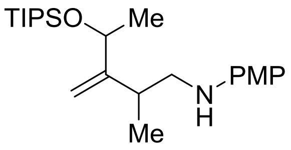

The reaction was conducted in accordance with **General Procedure B** (*via* diene **1d**). After heating the reaction for 24 hours, the mixture was concentrated *in vacuo* and purified by flash column chromatography (SiO_2, 5% EtOAc/hexanes) to furnish the title compound (55.6 mg, 71%) as a yellow oil.

^{1}H NMR (400 MHz, $CDCl_3$): δ 6.77 (d, *J* = 8.9 Hz, 2H), 6.57 (d, *J* = 9.0 Hz, 2H), 5.26 – 5.22 (m, 1H), 4.89 – 4.87 (m, 1H), 4.35 (dd, *J* = 12.8, 6.4 Hz, 1H), 3.74 (s, 3H), 3.53 (br, 1H), 3.21 – 3.00 (m, 2H), 2.48 (dd, *J* = 13.9, 7.0 Hz, 1H), 1.30 – 1.24 (m, 3H), 1.19 – 0.99 (m, 24H).

^{13}C NMR (100 MHz, $CDCl_3$): δ 156.90, 151.99, 142.80, 115.04, 115.02, 114.17, 114.15, 108.19, 107.92, 71.93, 55.98, 50.96, 50.44, 34.45, 34.09, 24.67, 24.52, 18.31, 12.55, 12.52.

LRMS (ESI): m/z 392 $[M+H]^+$

FTIR (neat): 2942, 2864, 1511, 1463, 1367, 1293, 1234, 1179, 1117, 1085, 1066, 1042, 1012, 996, 970, 917, 881, 816, 769 cm^{-1}.

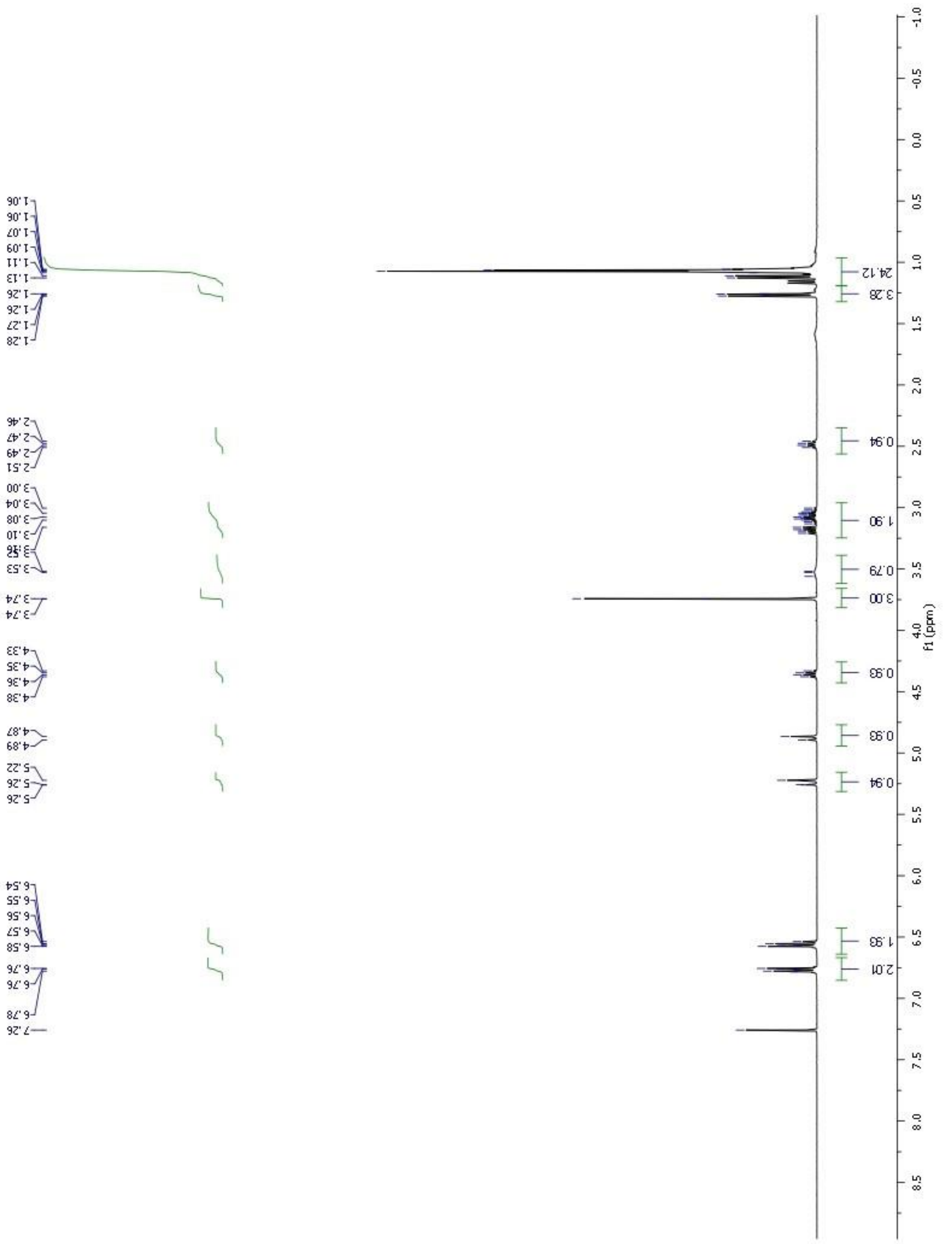
f1 (ppm)
24.12
3.28
0.94
1.90
0.79
3.00
0.93
0.93
0.94
1.93
2.01

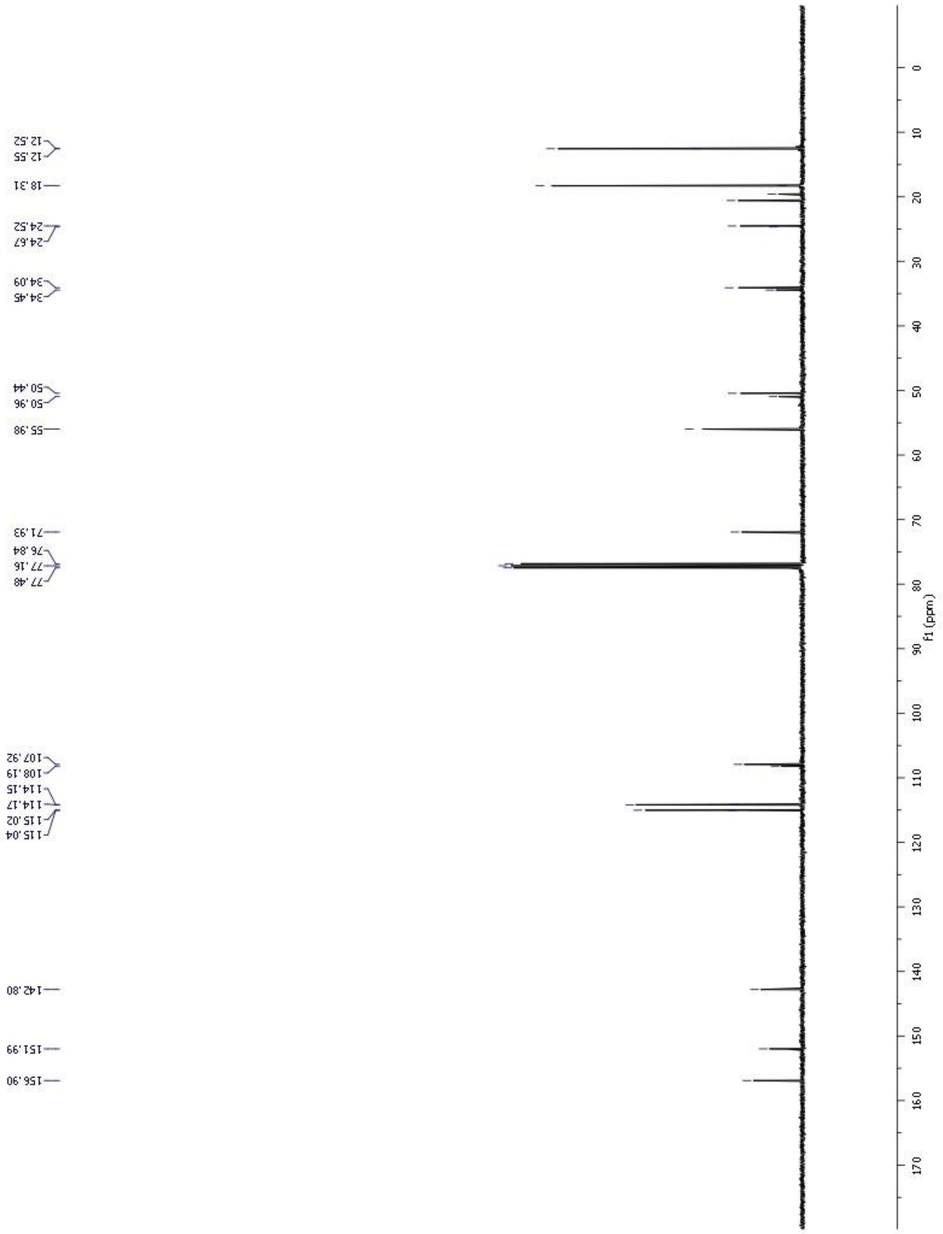
12.52
12.55
18.31
24.52
24.67
34.09
34.45
50.44
50.96
55.98
71.93
76.84
77.16
77.48
107.92
108.19
114.15
114.17
115.02
115.04
142.80
151.99
156.90
f1 (ppm)

***N*-(2-methyl-3-(*N*-benzyl-*N*-methylbenzenesulfonylamino)methylbut-3-en-1-yl)-4-methoxyaniline (3e)**

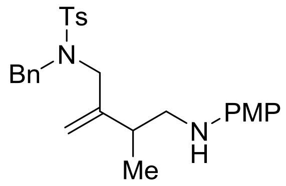

The reaction was conducted in accordance with **General Procedure B** (*via* diene **1e**). After heating the reaction for 24 hours, the mixture was concentrated *in vacuo* and purified by flash column chromatography (SiO_2, 17% EtOAc/hexanes) to furnish the title compound (68.7 mg, 74%) as a yellow oil.

^{1}H NMR (400 MHz, $CDCl_3$): δ 7.69 (d, *J* = 8.3 Hz, 2H), 7.28 (dd, *J* = 8.5, 0.6 Hz, 2H), 7.24 – 7.11 (m, 5H), 6.76 (d, *J* = 9.0 Hz, 2H), 6.51 (d, *J* = 9.0 Hz, 2H), 4.98 – 4.83 (m, 2H), 4.31 (dd, *J* = 61.4, 15.0 Hz, 2H), 3.75 (s, 3H), 3.00 – 2.85 (m, 2H), 2.48 – 2.31 (m, 3H), 0.90 (d, *J* = 7.0 Hz, 3H).

^{13}C NMR (100 MHz, $CDCl_3$): δ 152.07, 146.64, 143.49, 142.74, 137.04, 136.08, 129.84, 128.94, 128.55, 127.86, 127.43, 115.01, 114.20, 113.26, 56.01, 51.99, 51.89, 49.44, 35.97, 21.68, 17.76.

LRMS (CI): m/z 465 $[M+H]^+$

FTIR (neat): 3403, 2922, 1597, 1511, 1495, 1455, 1405, 1335, 1304, 1234, 1155, 1090, 1038, 924, 890, 816, 771, 735 cm^{-1}.

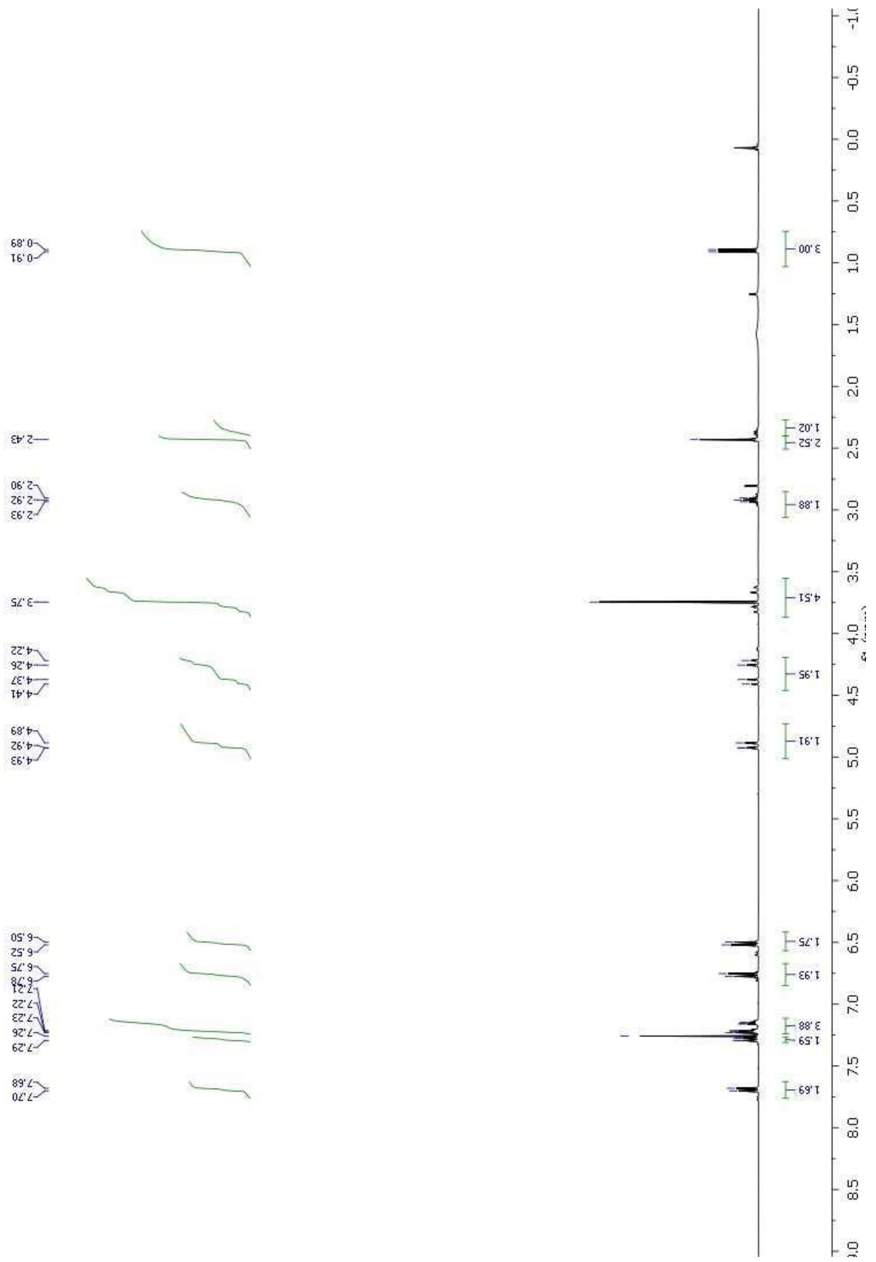

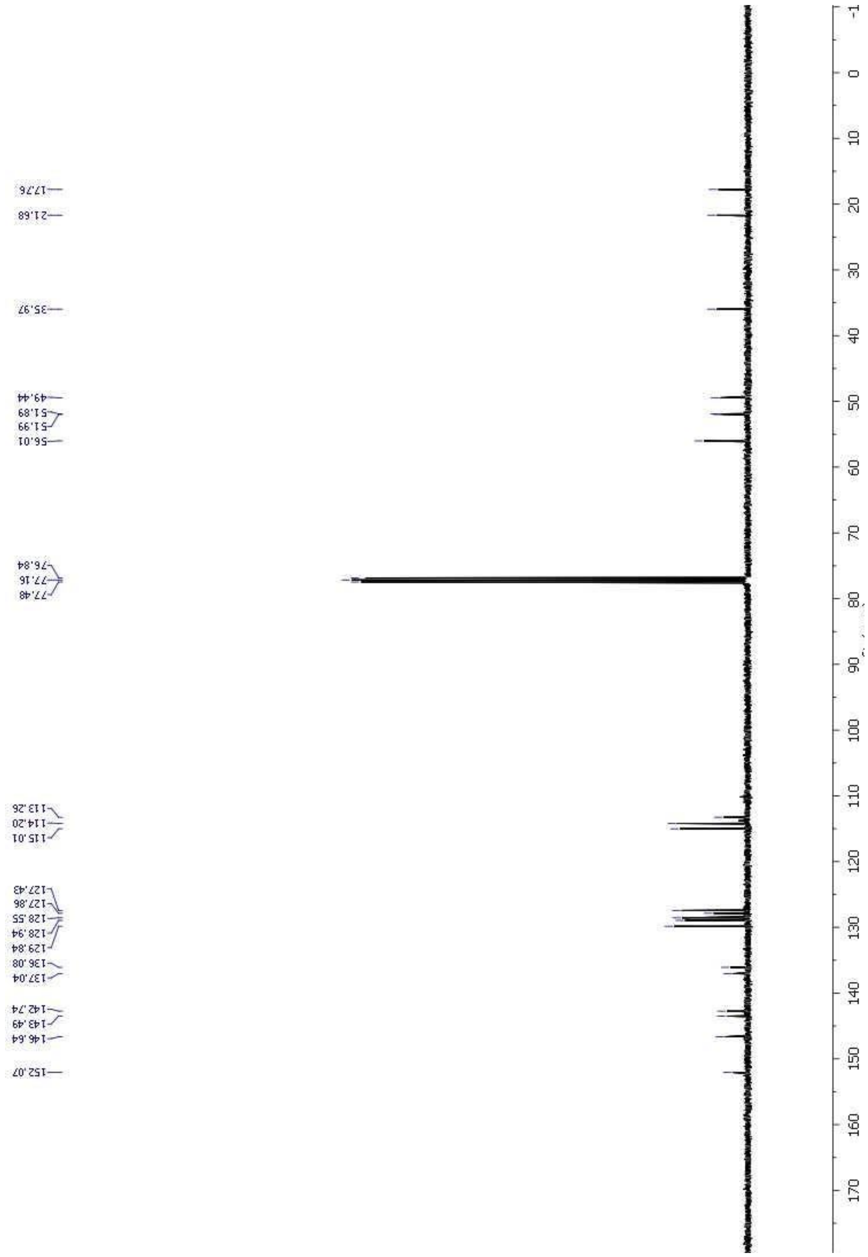
17.76
21.68
35.97
49.44
51.89
51.99
56.01
76.84
77.16
77.48
113.26
114.20
115.01
127.43
127.86
128.55
128.94
129.84
136.08
137.04
142.74
143.49
146.64
152.07

***N*-(2-methyl-3-cyclohexylbut-3-en-1-yl)-4-methoxyaniline (3f)**

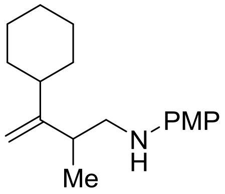

The reaction was conducted in accordance with **General Procedure B** (*via* diene **1f**). After heating the reaction for 24 hours, the mixture was concentrated *in vacuo* and purified by flash column chromatography (SiO_2, 5% EtOAc/hexanes) to furnish the title compound (38.2 mg, 70%) as a yellow oil.

^{1}H NMR (400 MHz, $CDCl_3$): δ 6.78 (d, *J* = 9.0 Hz, 1H), 6.57 (d, *J* = 9.0 Hz, 1H), 4.90 – 4.83 (m, 2H), 3.75 (s, 2H), 3.42 (br, 1H), 3.04 (d, *J* = 6.9 Hz, 2H), 2.46 (h, *J* = 6.8 Hz, 1H), 1.90 – 1.60 (m, 6H), 1.33 – 1.13 (m, 5H), 1.10 (d, *J* = 6.9 Hz, 3H).

^{13}C NMR (100 MHz, $CDCl_3$): δ 158.38, 152.08, 142.85, 115.00, 114.32, 107.82, 55.96, 49.79, 43.25, 39.11, 33.65, 33.61, 27.05, 27.00, 26.42, 19.12.

LRMS (ESI): m/z 274 $[M+H]^+$

FTIR (neat): 3389, 2923, 2850, 1637, 1510, 1448, 1407, 1295, 1232, 1178, 1123, 1038, 886, 816, 754 cm^{-1}.

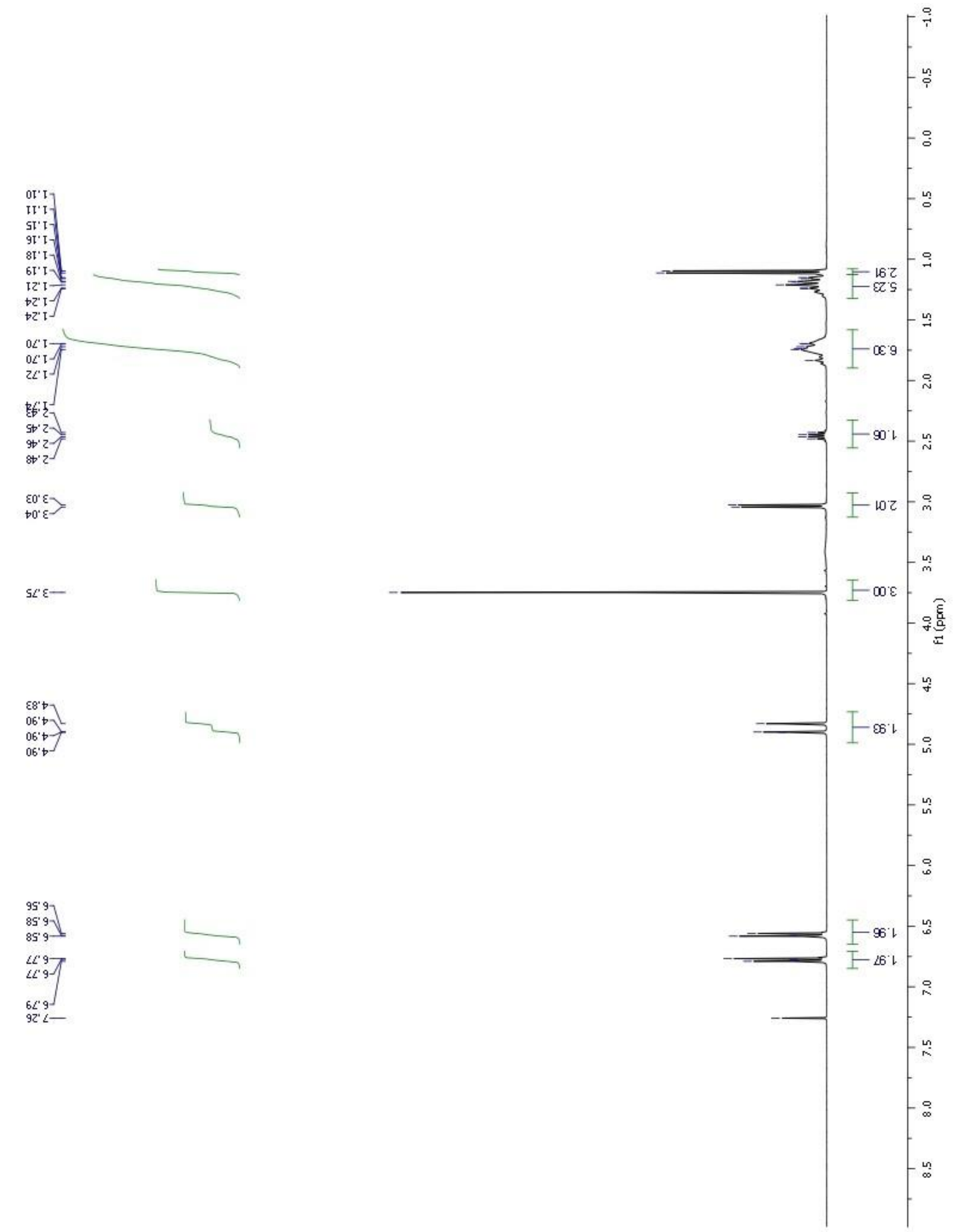

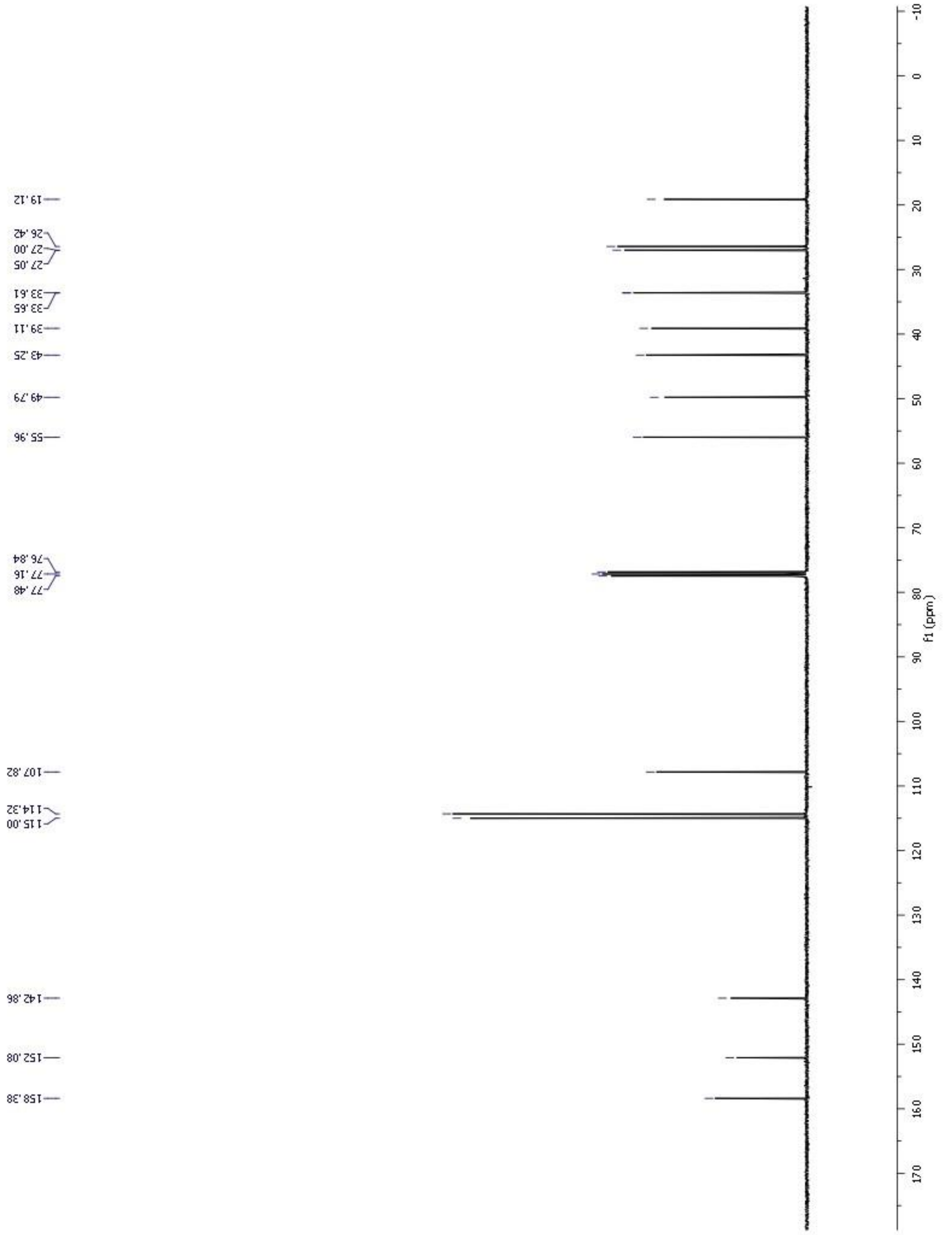
19.12
26.42
27.00
27.05
33.61
33.65
39.11
43.25
49.79
55.96
76.84
77.16
77.48
107.82
114.32
115.00
142.86
152.08
158.38
f1 (ppm)

N-(2-methyl-3-phenylbut-3-en-1-yl)-4-methoxyaniline (3g)

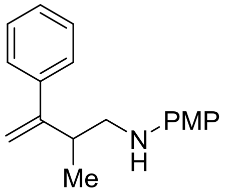

The reaction was conducted in accordance with **General Procedure B** (*via* diene **1g**). After heating the reaction for 24 hours, the mixture was concentrated *in vacuo* and purified by flash column chromatography (SiO_2, 6% EtOAc/hexane) to furnish the title compound (41.1 mg, 77%) as a yellow oil.

^{1}H NMR (400 MHz, $CDCl_3$): δ 7.39 – 7.27 (m, 5H), 6.78 (d, *J* = 9.0 Hz, 2H), 6.54 (d, *J* = 9.0 Hz, 2H), 5.32 (m, 1H), 5.15 (m, 1H), 3.75 (s, 3H), 3.49 (br, 1H), 3.17 (dt, *J* = 9.0, 4.5 Hz, 1H), 3.09 – 3.01 (m, 2H), 1.22 (d, *J* = 6.6 Hz, 3H).

^{13}C NMR (100 MHz, $CDCl_3$): δ 152.43, 152.13, 142.62, 142.27, 128.42, 127.54, 126.86, 115.00, 114.31, 112.73, 55.95, 49.94, 38.17, 18.52.

LRMS (ESI): m/z 268 $[M+H]^+$

FTIR (neat): 3397, 2930, 2829, 1619, 1509, 1463, 1441, 1407, 1294, 1233, 1178, 1122, 1035, 900, 817, 777, 700 cm^{-1}.

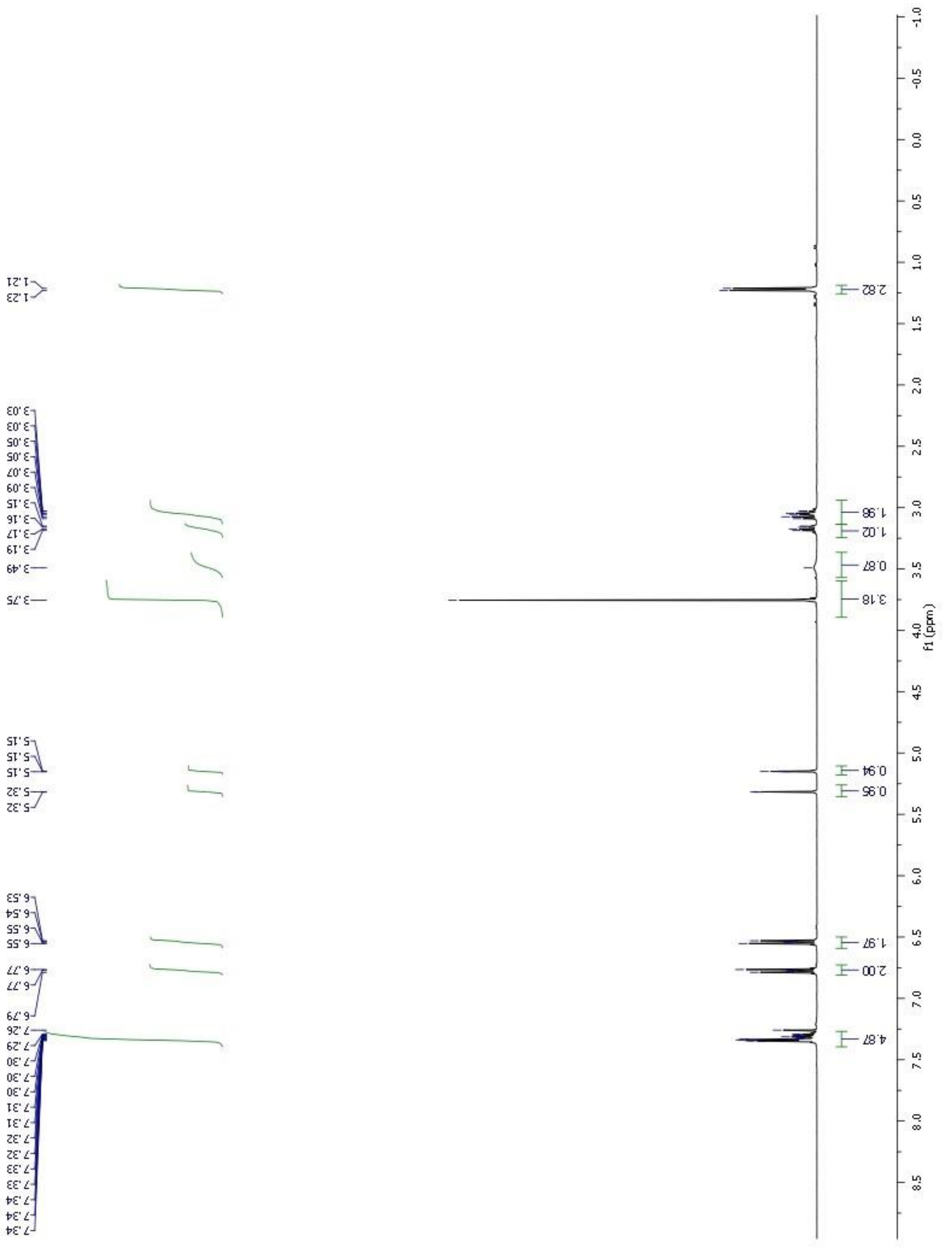
1.21
1.23
3.03
3.03
3.05
3.05
3.07
3.09
3.15
3.16
3.17
3.19
3.49
3.75
5.15
5.15
5.15
5.32
5.32
6.53
6.54
6.55
6.55
6.77
6.77
6.79
7.26
7.29
7.30
7.30
7.30
7.31
7.31
7.32
7.32
7.33
7.33
7.34
7.34
7.34
2.82
1.98
1.02
0.87
3.18
0.94
0.96
1.97
2.00
4.87
f1 (ppm)

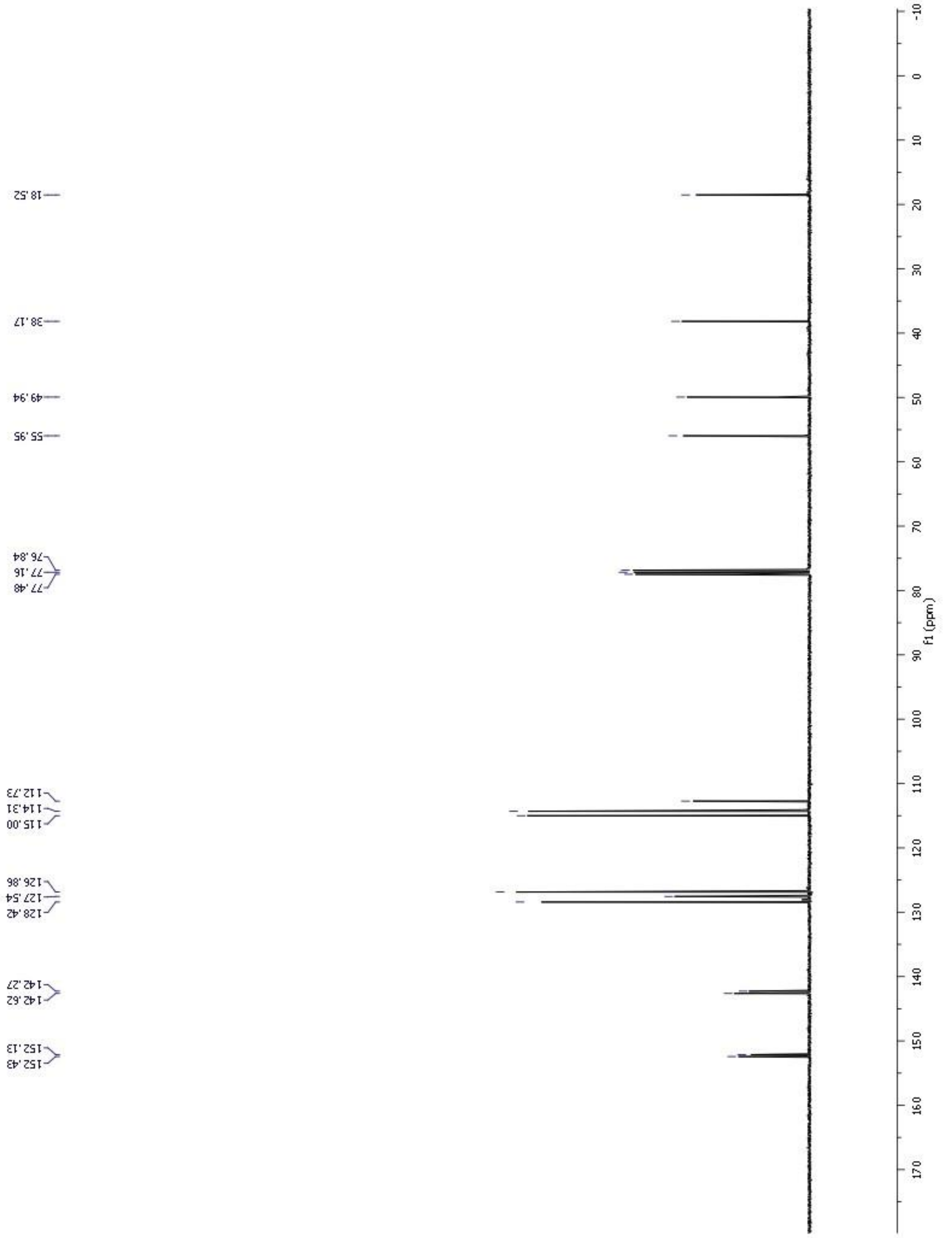

18.52
38.17
49.94
55.95
76.84
77.16
77.48
112.73
114.31
115.00
126.86
127.54
128.42
142.27
142.62
152.13
152.43
f1 (ppm)

***N*-(2-methyl-3-(4-methoxyphenyl)but-3-en-1-yl)-4-methoxyaniline (3h)**

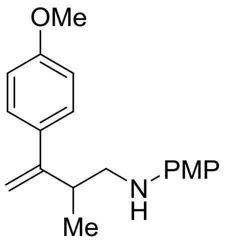

The reaction was conducted in accordance with **General Procedure B** (*via* diene **1h**). After heating the reaction for 24 hours, the mixture was concentrated *in vacuo* and purified by flash column chromatography (SiO_2, 10% EtOAc/hexanes) to furnish the title compound (42.8 mg, 72%) as a yellow oil.

^{1}H NMR (400 MHz, $CDCl_3$): 7.28 (d, *J* = 8.9 Hz, 2H), 6.86 (d, *J* = 8.9 Hz, 2H), 6.76 (d, *J* = 9.0 Hz, 2H), 6.53 (d, *J* = 9.1 Hz, 2H), 5.25 (m, 1H), 5.06 (m, 1H), 3.81 (s, 3H), 3.74 (s, 3H), 3.48 (br, 1H), 3.16 (dd, *J* = 11.7, 6.6 Hz, 1H), 3.10 – 2.91 (m, 2H), 1.20 (d, *J* = 6.7 Hz, 3H).

^{13}C NMR (100 MHz, $CDCl_3$): δ 159.20, 152.13, 151.72, 142.68, 134.63, 127.90, 115.01, 114.34, 113.79, 111.49, 55.97, 55.42, 50.00, 38.06, 18.59.

LRMS (CI): m/z 320 $[M+Na]^+$

FTIR (neat): 2956, 2833, 1606, 1508, 1463, 1408, 1374, 1293, 1240, 1178, 1116, 1031, 894, 833, 818, 745 cm^{-1}.

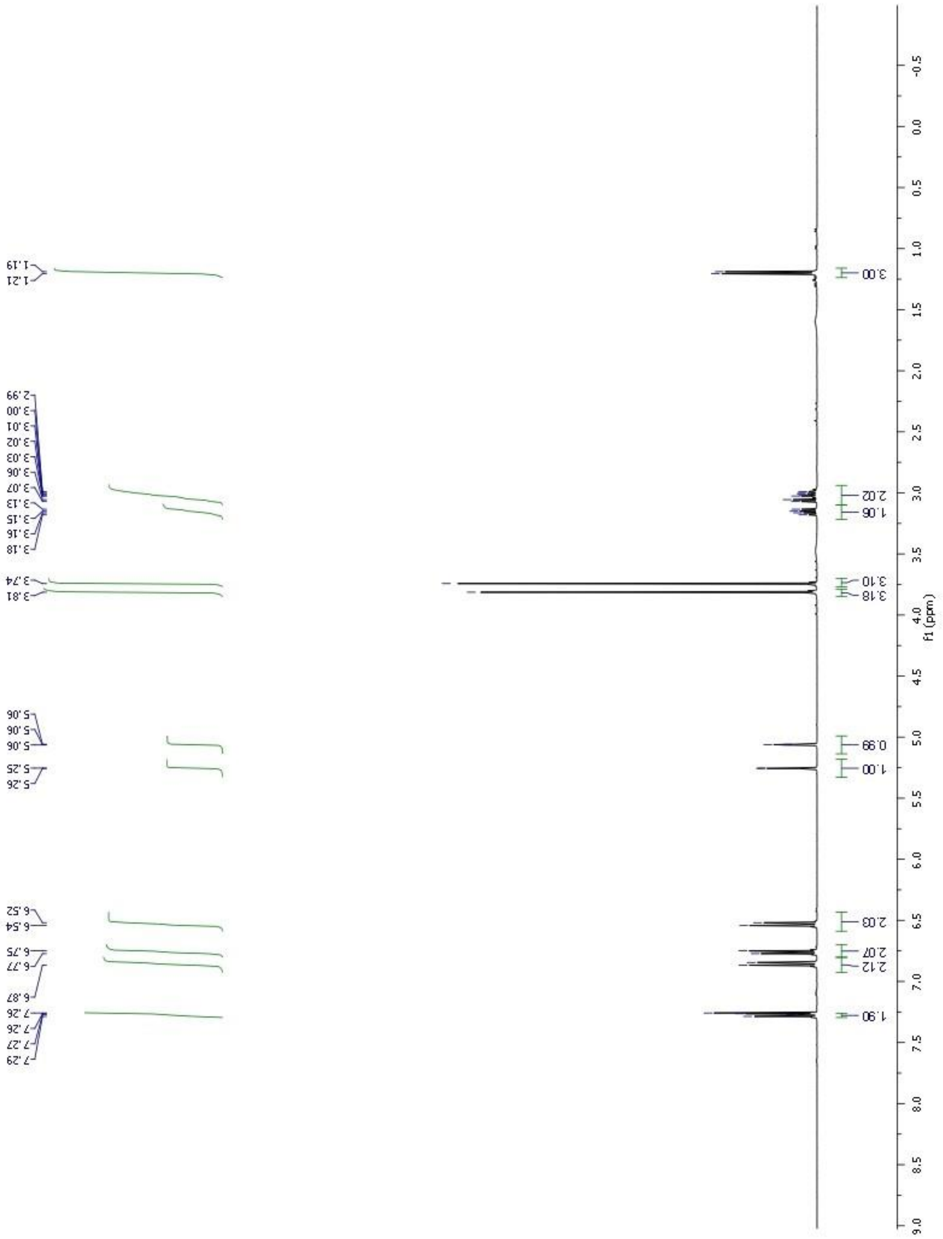

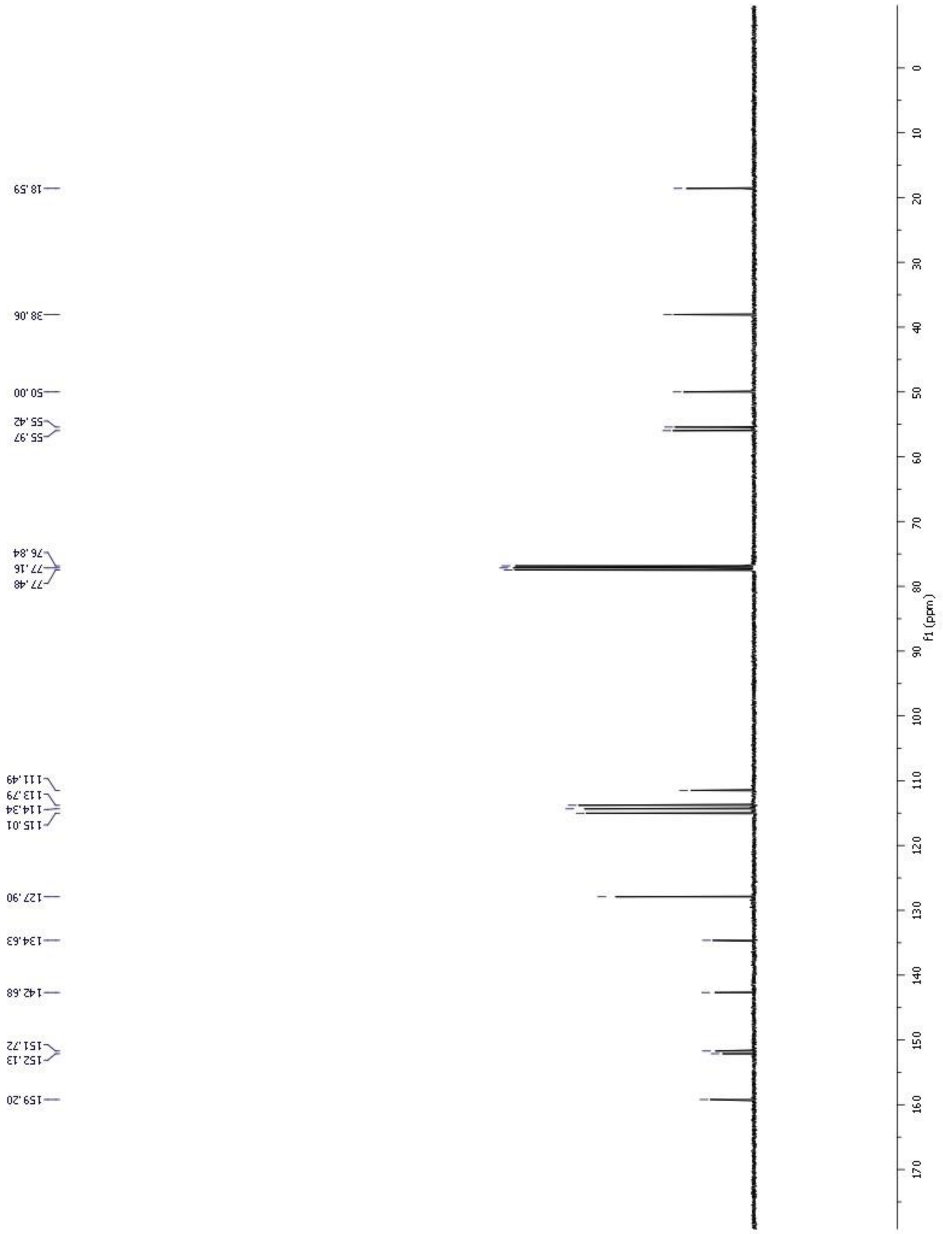

18.59
38.06
50.00
55.42
55.97
76.84
77.16
77.48
111.49
113.79
114.34
115.01
127.90
134.63
142.68
151.72
152.13
159.20
0
10
20
30
40
50
60
70
80
90
100
110
120
130
140
150
160
170
f1 (ppm)

***N*-(2-methyl-3-(4-trifluoromethylphenyl)but-3-en-1-yl)-4-methoxyaniline (3i)**

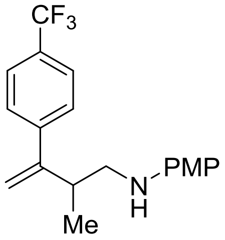

The reaction was conducted in accordance with **General Procedure B** (*via* diene **1i**). After heating the reaction for 24 hours, the mixture was concentrated *in vacuo* and purified by flash column chromatography (SiO_2, 15% EtOAc/hexanes) to furnish the title compound (54.3 mg, 81%) as a yellow oil.

^{1}H NMR (400 MHz, $CDCl_3$): δ 7.58 (dd, *J* = 8.7, 0.7 Hz, 2H), 7.42 (dd, *J* = 8.7, 0.7 Hz, 2H), 6.77 (d, *J* = 9.0 Hz, 2H), 6.53 (d, *J* = 9.1 Hz, 2H), 5.35 (m, 1H), 5.23 (m, 1H), 3.75 (s, 1H), 3.45 (br, 1H), 3.17 (dd, J = 11.9, 6.6 Hz, 1H), 3.11 – 2.95 (m, 2H), 1.21 (d, *J* = 6.7 Hz, 3H).

^{13}C NMR (100 MHz, $CDCl_3$): δ 152.28, 151.49, 145.93, 145.92, 142.36, 129.61 (q, *J* = 32.5 Hz), 127.23, 125.39 (q, *J* = 3.8 Hz), 124.32 (q, *J* = 270.6 Hz), 115.05, 114.40, 114.32, 55.94, 49.85, 38.17, 18.40.

^{19}F NMR (376 MHz, $CDCl_3$): δ -62.48.

LRMS (CI): m/z 336 $[M+H]^+$

FTIR (neat): 2933, 1615, 1510, 1464, 1405, 1323, 1234, 1163, 1117, 1063, 1036, 1014, 908, 849, 817, 758, 714 cm^{-1}.

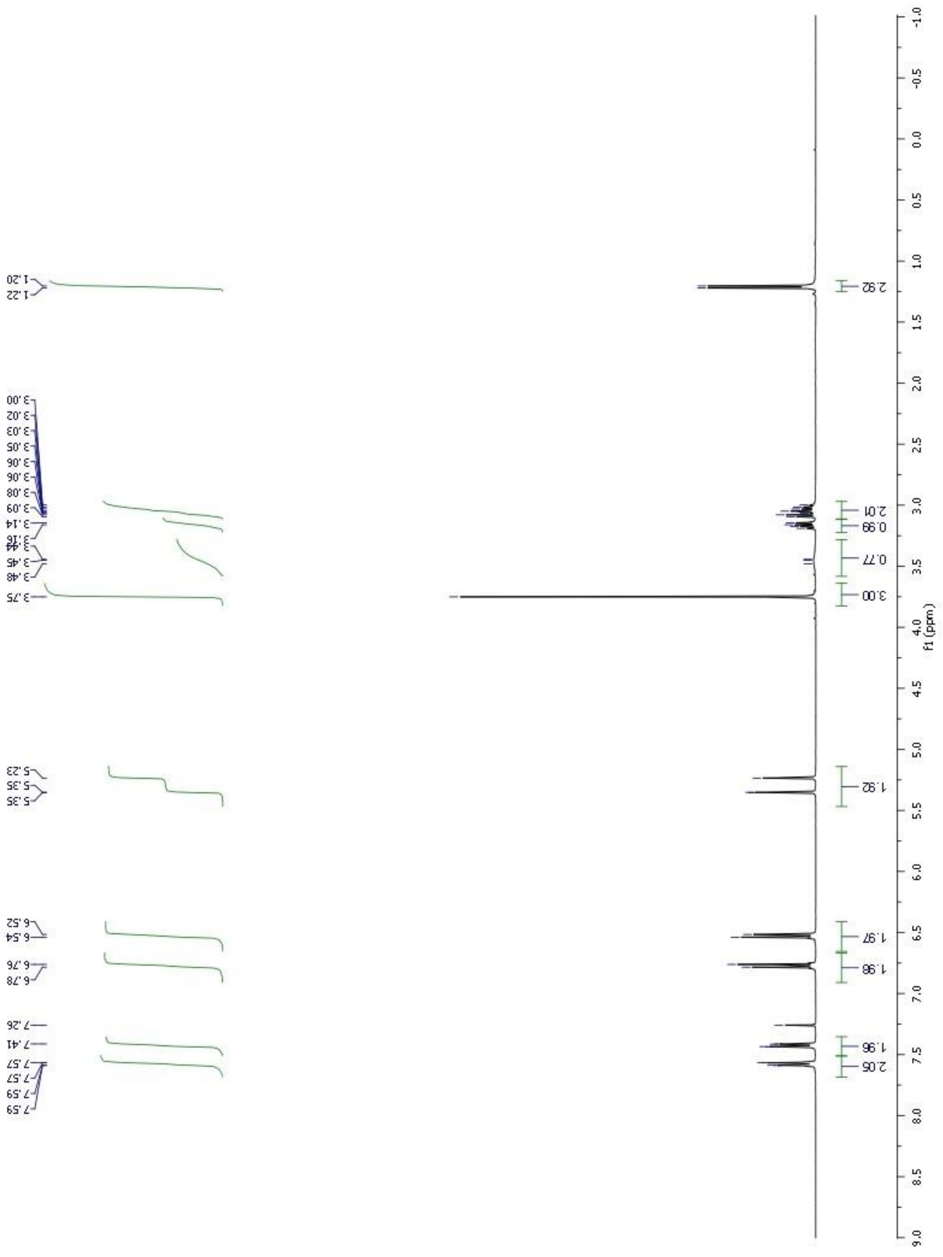
f1 (ppm)

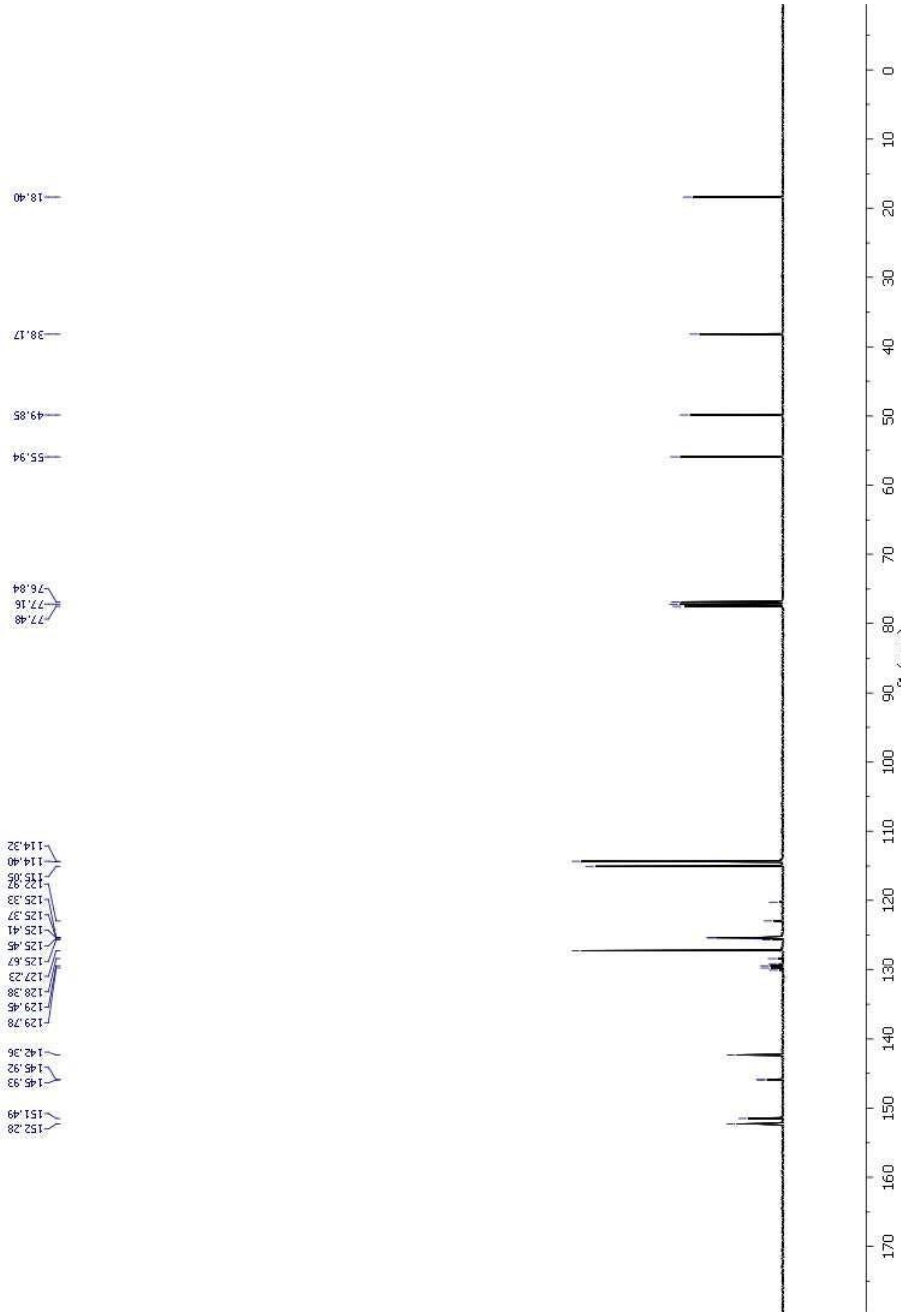

18.40
38.17
49.85
55.94
76.84
77.16
77.48
114.32
114.40
115.05
122.92
125.33
125.37
125.41
125.45
125.67
127.23
128.38
129.45
129.78
142.36
145.92
145.93
151.49
152.28
0
10
20
30
40
50
60
70
80
90
100
110
120
130
140
150
160
170

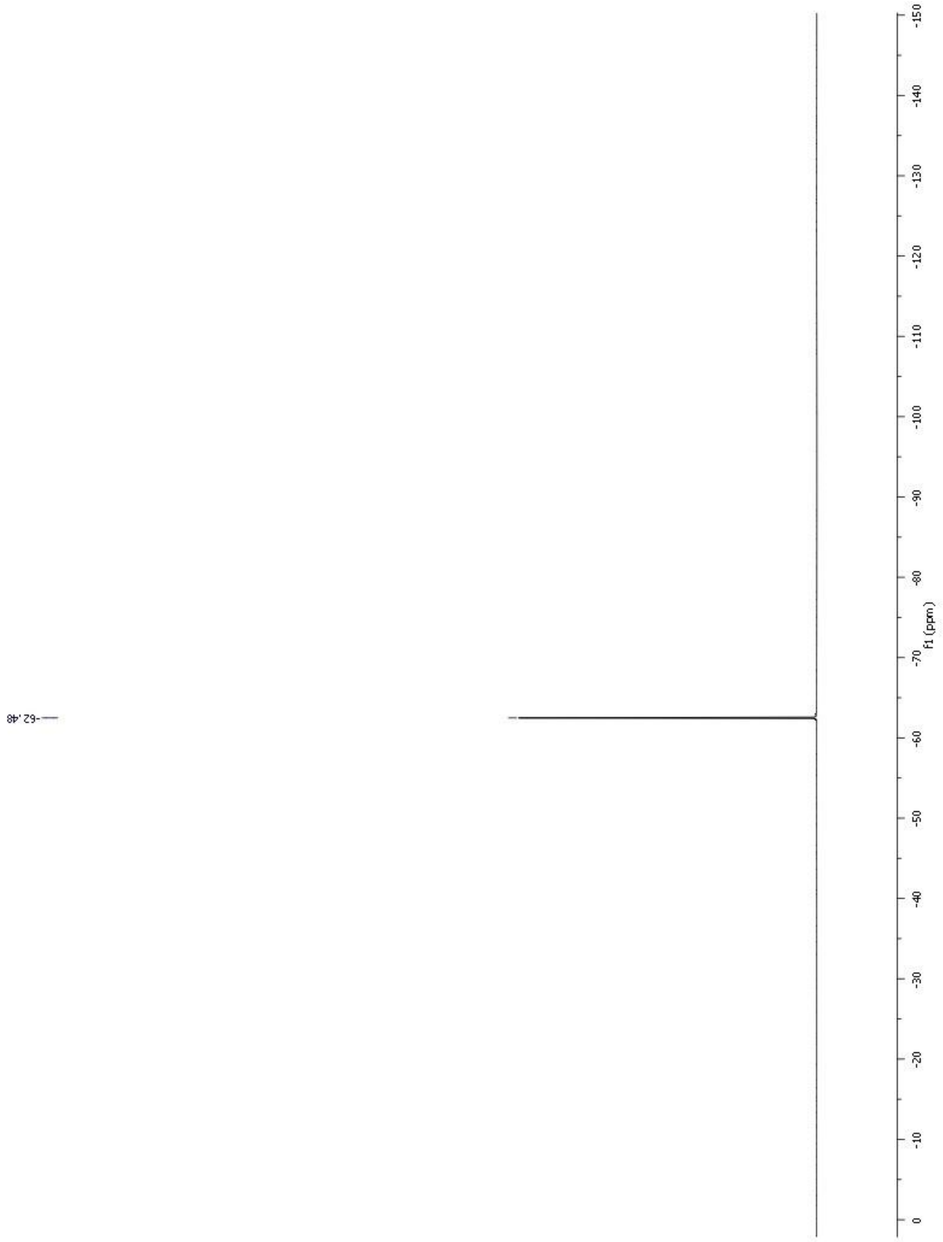
-62.48
f1 (ppm)
0
-10
-20
-30
-40
-50
-60
-70
-80
-90
-100
-110
-120
-130
-140
-150

III. Experimental Procedures and Spectral Data Adducts 4a-8a

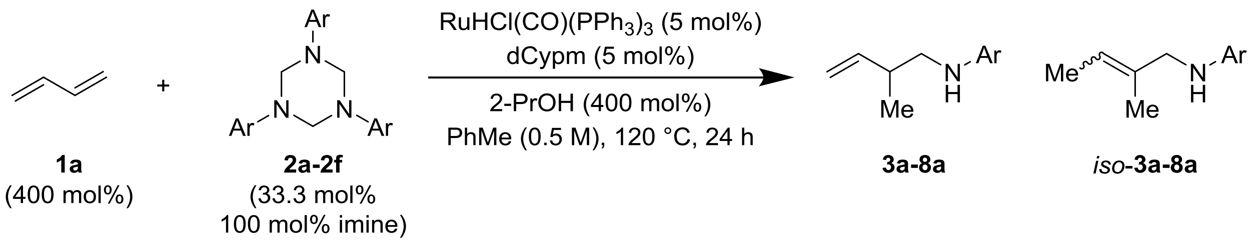

General Procedure C for the coupling of 1,3-butadiene 1a with triazine 2b-2f

To an oven-dried pressure tube equipped with magnetic stir bar was added $RuHCl(CO)(PPh_3)_3$ (9.5 mg, 0.010 mmol, 5 mol%), bis(dicyclohexylphosphino)methane (dCypm) (4.1 mg, 0.010 mmol, 5 mol%), and triazine (0.067 mmol 33.3 mol% (0.200 mol, 100 mol% of imine)). The tube was sealed with a rubber septum, purged with argon, and toluene (0.4 mL, 0.5 M with respect to formimine), 1,3-butadiene **1a** (70 µL, 0.800 mmol, 400 mol%), and isopropanol (61 µL, 0.800 mmol, 400 mol%) were added. The rubber septum was quickly replaced with a screw cap and the reaction was heated to 120 °C for 24 hours. The reaction mixture was allowed to cool to room temperature, concentrated *in vacuo*, and purified by flash column chromatography (SiO_2) to furnish the title compounds.

***N*-(2-methylbut-3-en-1-yl)-4-dimethylaminoaniline (4a)**

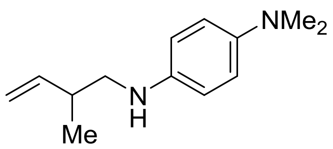

The reaction was conducted in accordance with **General Procedure C** (*via* triazine **2b**). After heating the reaction for 24 hours, the mixture was concentrated *in vacuo* and purified by flash column chromatography (SiO_2, 20% EtOAc/hexanes) to furnish the title compound (33.5 mg, 82%) as a yellow oil.

^{1}H NMR (400 MHz, *d6*-acetone): δ 6.67 (d, *J* = 9.0 Hz, 2H), 6.57 (d, *J* = 9.0 Hz, 2H), 5.92 – 5.66 (m, 1H), 5.03 (dddd, J = 22.0, 10.4, 1.9, 1.1 Hz, 2H), 2.97 (d, J = 6.9 Hz, 2H), 2.90 – 2.78 (m, 1H), 2.75 (s, 6H), 2.46 (ddd, *J* = 8.4, 4.5, 3.4 Hz, 1H), 1.06 (d, *J* = 6.7 Hz, 3H).

^{13}C NMR (100 MHz, *d6*-acetone): δ 144.60, 143.56, 142.38, 116.54, 114.72, 114.49, 51.19, 42.42, 38.40, 18.14.

LRMS (ESI): m/z 205 $[M+H]^+$

FTIR (neat): 2867, 1672, 1610, 1517, 1444, 1351, 1318, 1257, 1222, 1165, 1131, 1059, 946, 816 cm^{-1}.

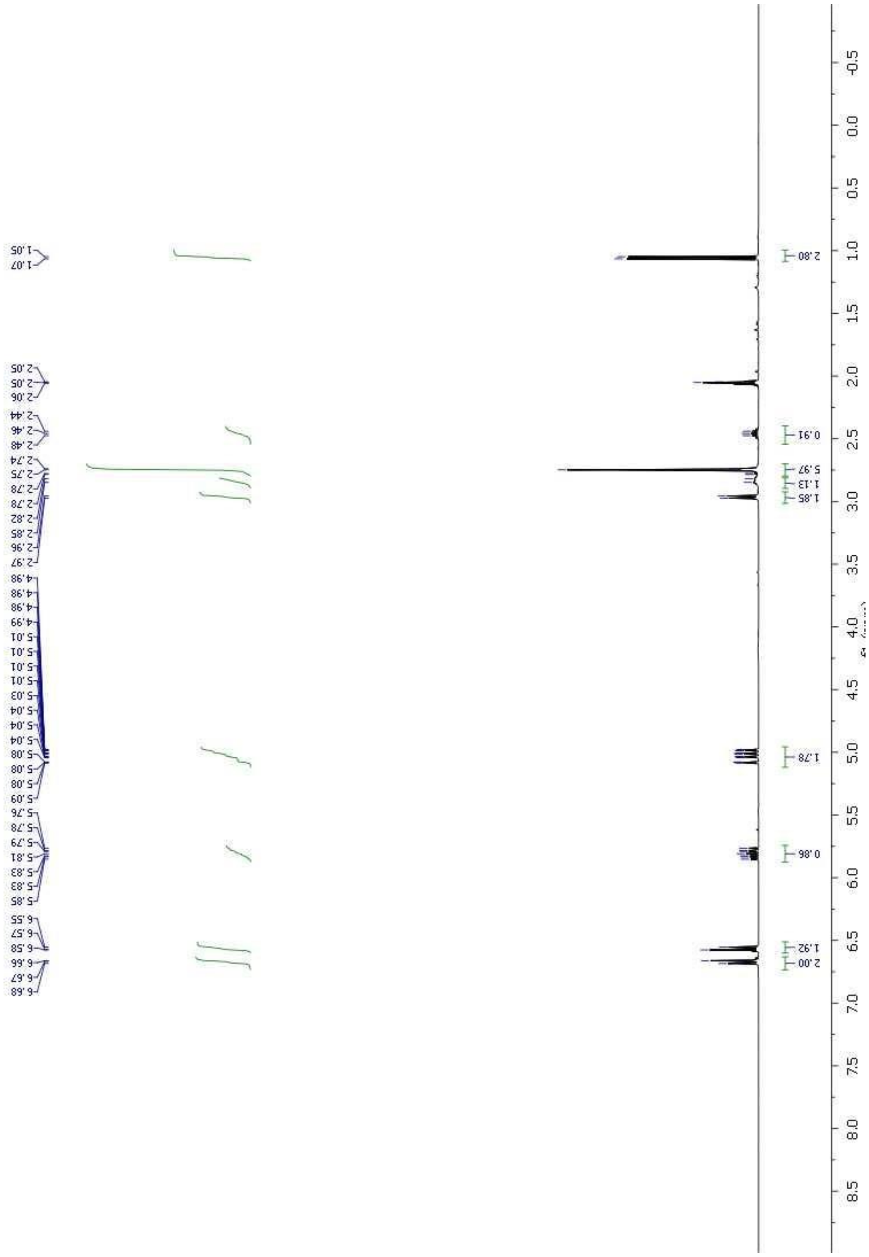

-0.5
0.0
0.5
1.0
1.5
2.0
2.5
3.0
3.5
4.0
4.5
5.0
5.5
6.0
6.5
7.0
7.5
8.0
8.5
2.80
0.91
5.97
1.13
1.85
1.78
0.86
1.92
2.00

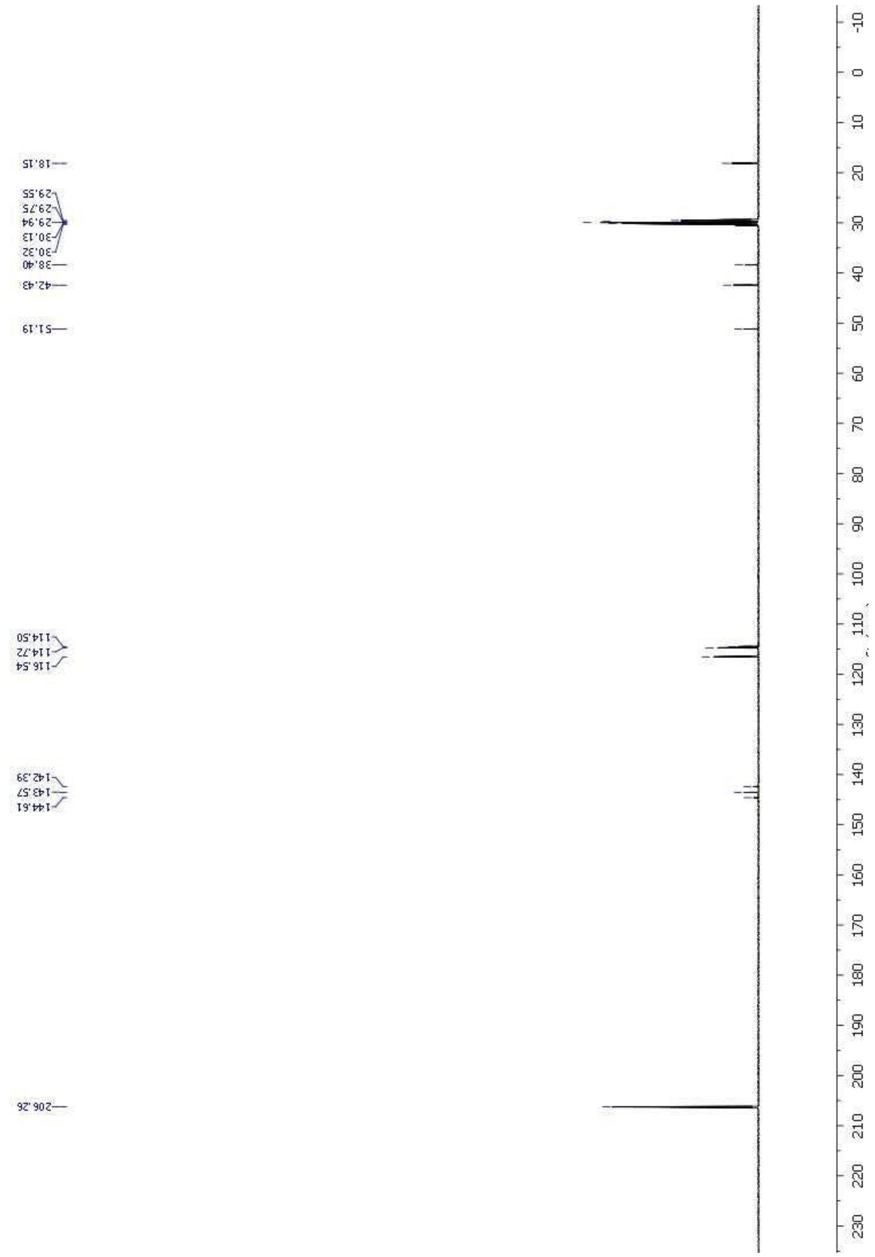

18.15
29.55
29.75
29.94
30.13
30.32
38.40
42.43
51.19
114.50
114.72
116.54
142.39
143.57
144.61
206.26
-10 0 10 20 30 40 50 60 70 80 90 100 110 120 130 140 150 160 170 180 190 200 210 220 230

***N*-(2-methylbut-3-en-1-yl)-2-methoxyaniline (5a)**

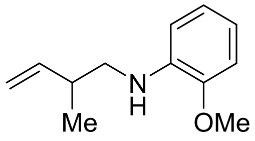

The reaction was conducted in accordance with **General Procedure C** (*via* triazine **2c**). After heating the reaction for 24 hours, the mixture was concentrated *in vacuo* and purified by flash column chromatography (SiO_2, 5% EtOAc/hexanes) to furnish the title compound (27.9 mg, 73%) as a yellow oil.

^{1}H NMR (400 MHz, $CDCl_3$): δ 6.90 – 6.83 (m, 1H), 6.77 (dd, *J* = 7.9, 1.4 Hz, 1H), 6.71 – 6.55 (m, 2H), 5.77 (ddd, *J* = 17.3, 10.3, 7.6 Hz, 1H), 5.10 (dddd, *J* = 13.9, 10.3, 1.7, 1.1 Hz, 2H), 4.31 (br, 1H), 3.84 (s, 3H), 3.10 (dd, *J* = 12.0, 6.0 Hz, 1H), 3.00 (dd, *J* = 12.0, 7.8 Hz, 1H), 2.52 (dtd, *J* = 13.7, 6.8, 1.0 Hz, 1H), 1.11 (d, *J* = 6.8 Hz, 3H).

^{13}C NMR (100 MHz, $CDCl_3$): δ 146.97, 142.16, 138.44, 121.40, 116.34, 114.96, 110.02, 109.58, 55.60, 49.30, 37.76, 18.17.

LRMS (ESI): m/z 192 $[M+H]^+$

FTIR (neat): 3424, 3066, 2956, 2833, 1601, 1511, 1455, 1428, 1343, 1302, 1246, 1220, 1177, 1126, 1047, 1029, 995, 914, 817, 732 cm^{-1}.

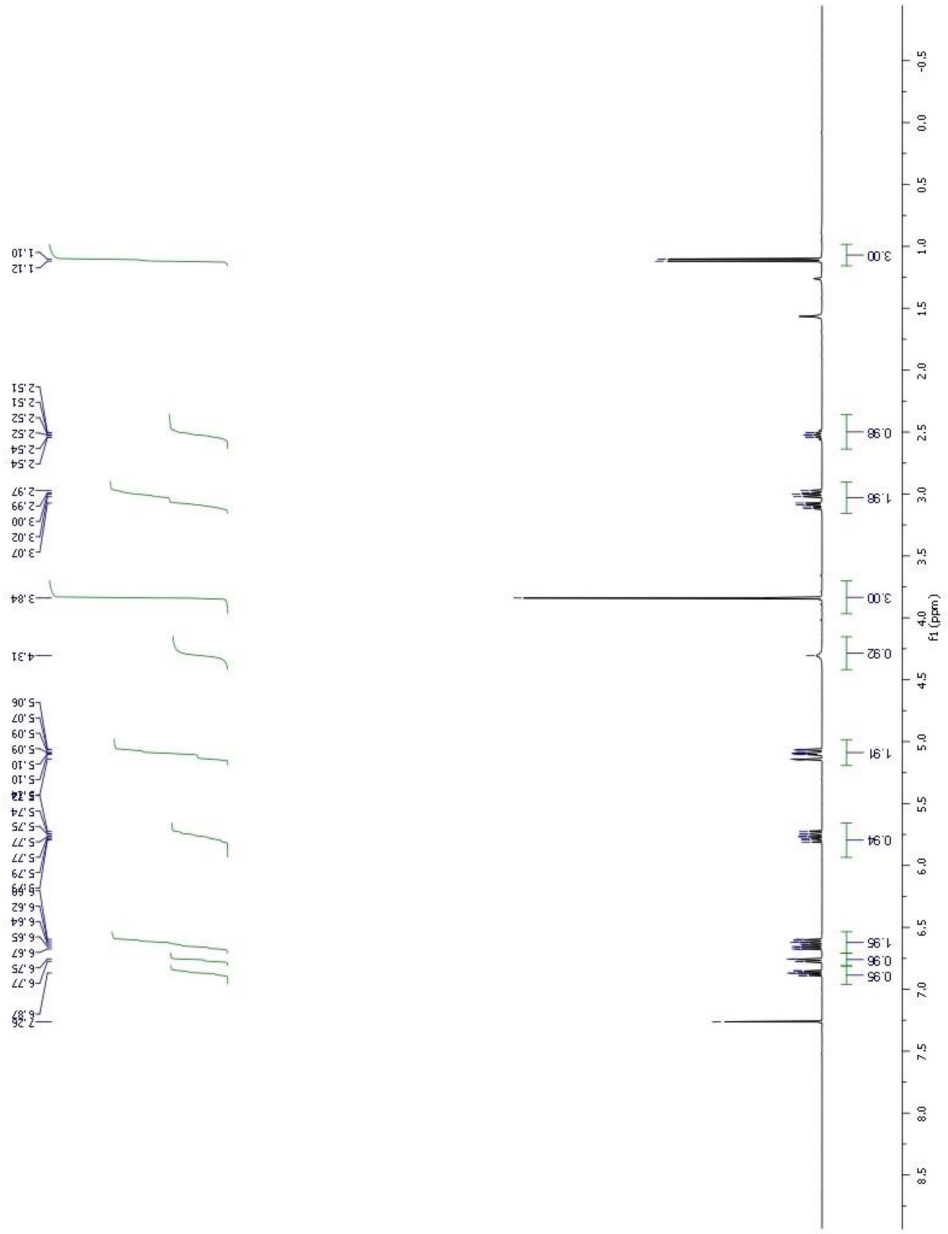
f1 (ppm)

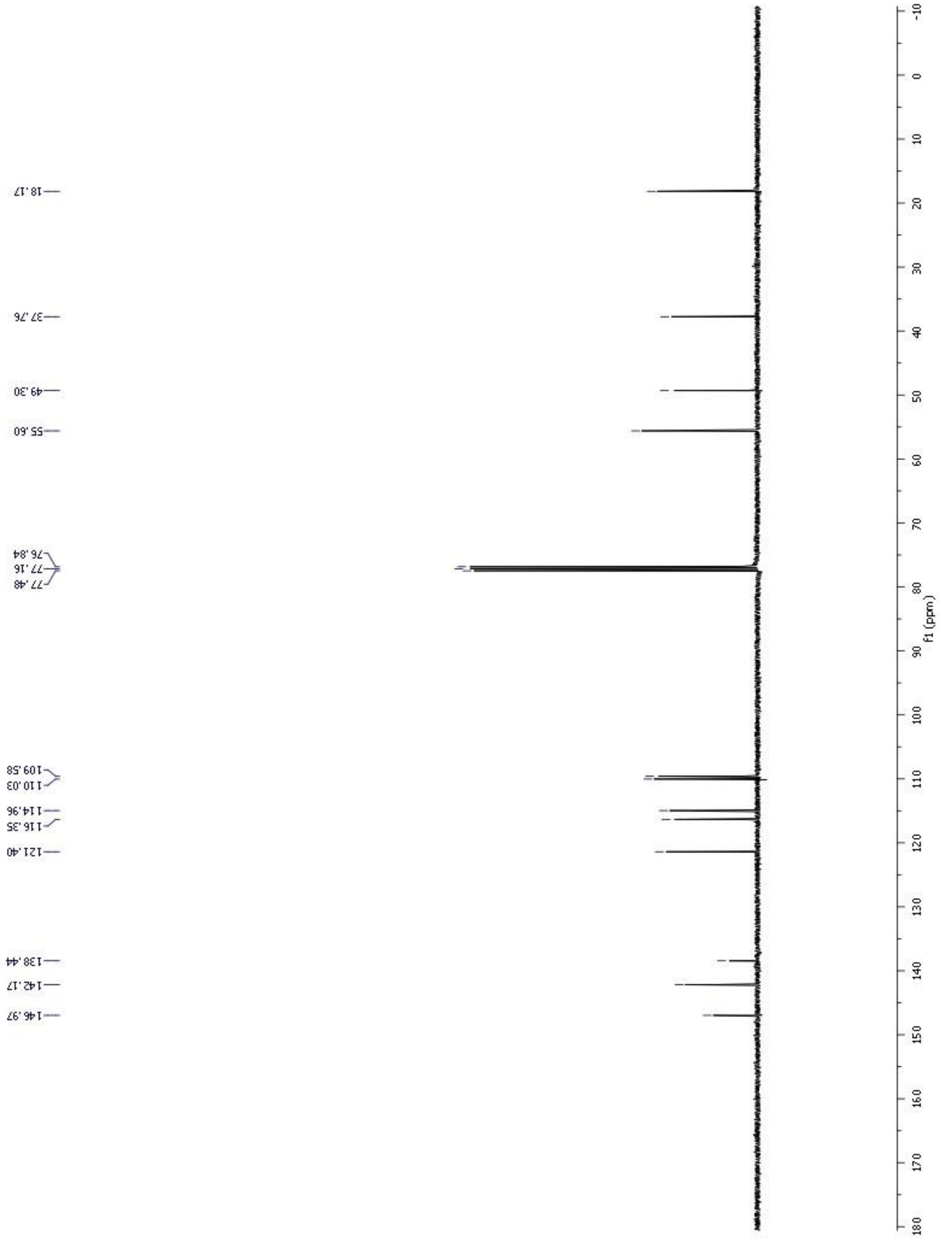
18.17
37.76
49.30
55.60
76.84
77.16
77.48
109.58
110.03
114.96
116.35
121.40
138.44
142.17
146.97
f1 (ppm)

***N*-(2-methylbut-3-en-1-yl)-aniline (6a)**

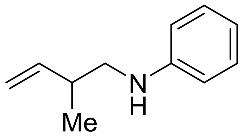

The reaction was conducted in accordance with **General Procedure C** (*via* triazine **2d**). After heating the reaction for 24 hours, the mixture was concentrated *in vacuo* and purified by flash column chromatography (SiO_2, 5% EtOAc/hexanes) to furnish the title compound (26.1 mg, 81%) as a yellow oil.

^{1}H NMR (400 MHz, $CDCl_3$): δ 7.23 – 7.10 (m, 2H), 6.70 (tt, *J* = 7.3, 1.1 Hz, 1H), 6.61 (ddd, *J* = 3.0, 2.5, 1.5 Hz, 1H), 5.73 (ddd, *J* = 17.3, 10.3, 7.8 Hz, 1H), 5.19 – 5.02 (m, 2H), 3.71 (br, 1H), 3.11 (dd, *J* = 11.9, 5.6 Hz, 1H), 2.95 (dd, *J* = 11.9, 8.2 Hz, 1H), 2.63 – 2.37 (m, 1H), 1.11 (d, *J* = 6.8 Hz, 3H).

^{13}C NMR (100 MHz, $CDCl_3$): δ 148.46, 142.10, 129.36, 117.38, 115.29, 113.04, 49.41, 37.92, 18.17.

LRMS (ESI): m/z 162 $[M+H]^+$

FTIR (neat): 3411, 3052, 2925, 1912, 1601, 1504, 1472, 1431, 1375, 1316, 1253, 1178, 1153, 1070, 992, 914, 866, 812, 746, 690 cm^{-1}.

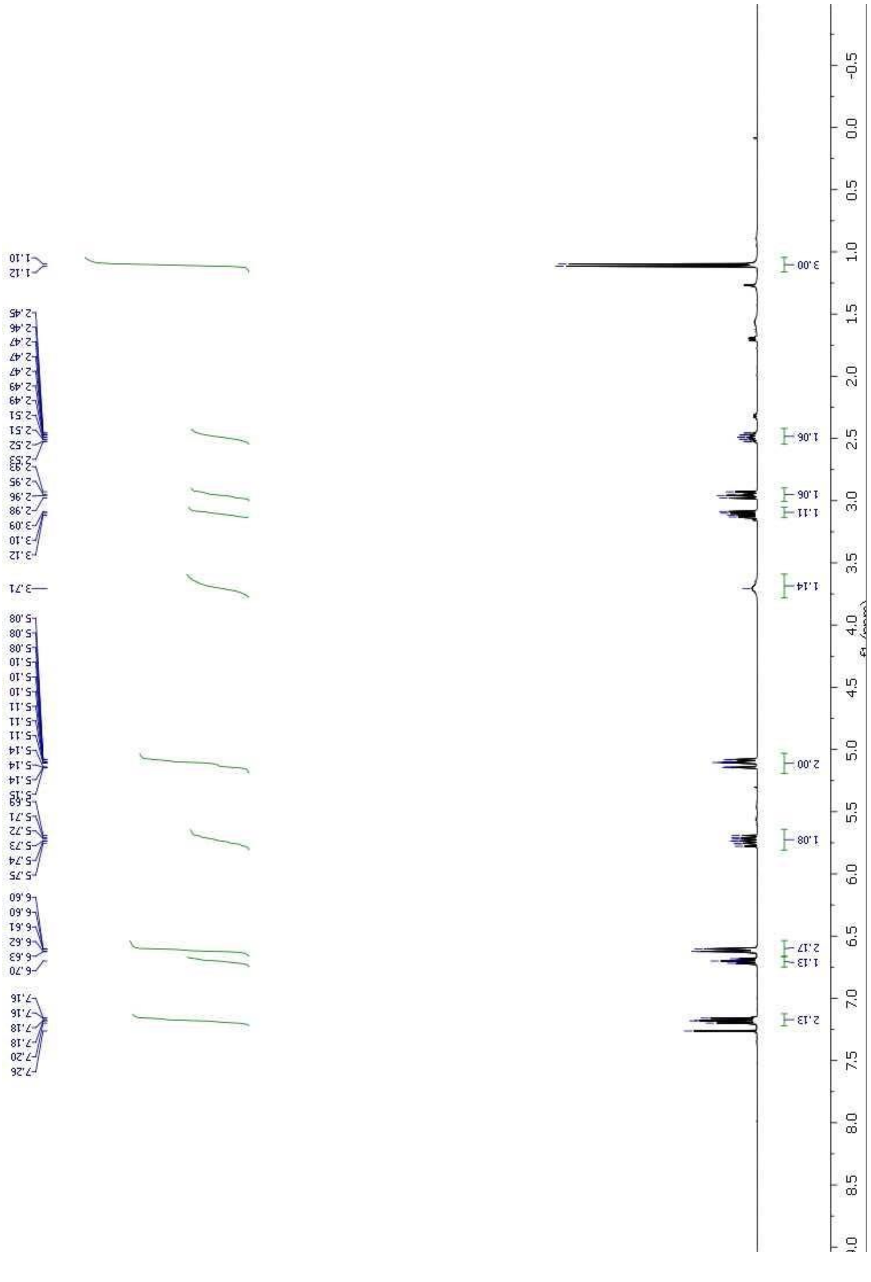

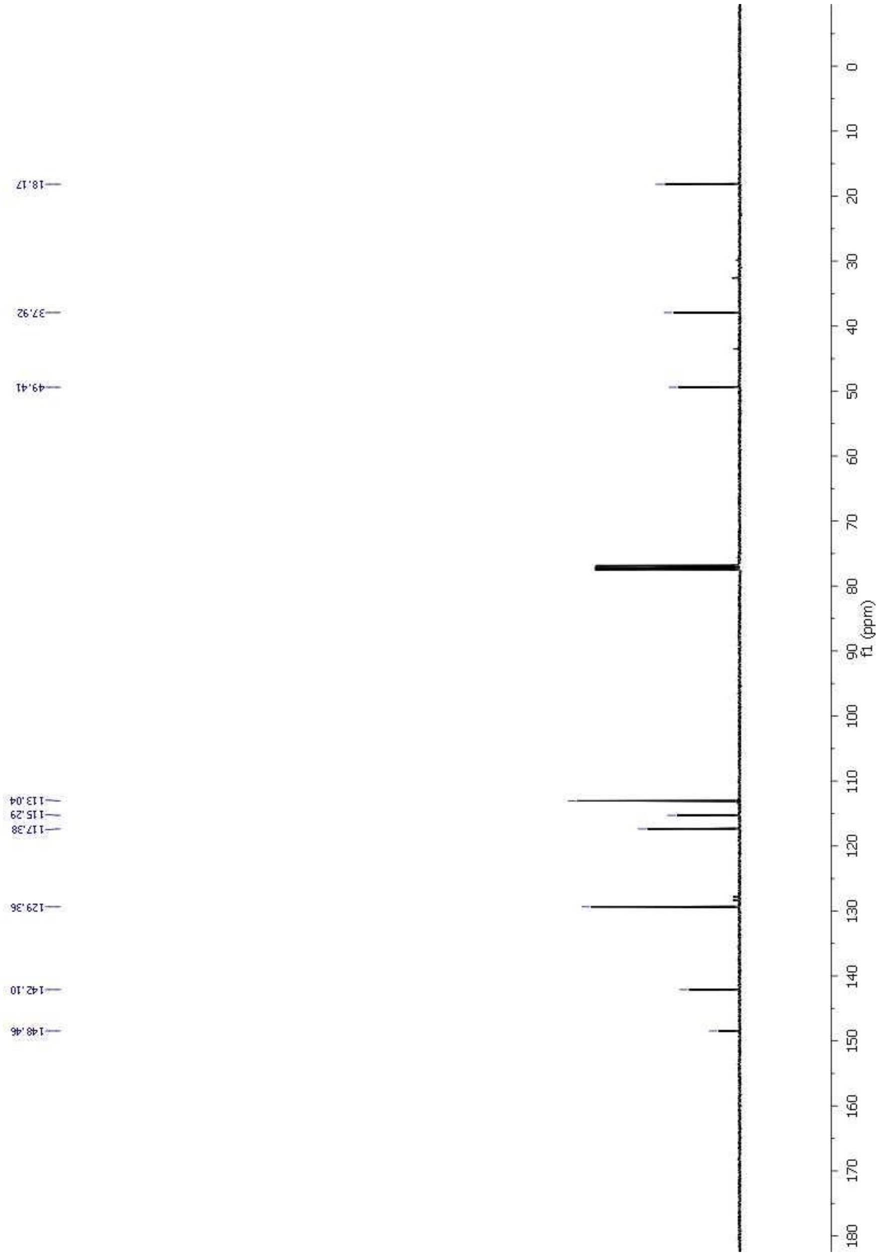
18.17
37.92
49.41
113.04
115.29
117.38
129.36
142.10
148.46
f1 (ppm)

***N*-(2-methylbut-3-en-1-yl)-4-fluoroaniline (7a)**

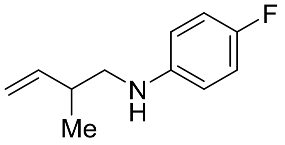

The reaction was conducted in accordance with **General Procedure C** (*via* triazine **2e**). After heating the reaction for 24 hours, the mixture was concentrated *in vacuo* and purified by flash column chromatography (SiO_2, 6% EtOAc/hexanes) to furnish the title compound (26.8 mg, 75%) as a yellow oil.

^{1}H NMR (400 MHz, $CDCl_3$): δ 6.88 (t, *J* = 8.7 Hz, 2H), 6.53 (dd, *J* = 8.9, 4.4 Hz, 2H), 5.81 – 5.58 (m, 1H), 5.16 – 4.96 (m, 2H), 3.58 (br, 1H), 3.06 (dd, *J* = 11.8, 5.4 Hz, 1H), 2.89 (dd, *J* = 11.7, 8.3 Hz, 1H), 2.61 – 2.31 (m, 1H), 1.09 (d, *J* = 6.8 Hz, 3H).

^{13}C NMR (100 MHz, $CDCl_3$): δ 155.87 (d, *J* = 234.6 Hz), 144.81 (d, *J* = 1.8 Hz), 142.00, 115.85, 115.54 (d, J = 18.3 Hz), 113.82 (d, *J* = 7.4 Hz), 50.06, 37.93, 18.19.

^{19}F NMR (376 MHz, $CDCl_3$): δ -128.37.

LRMS (ESI): m/z 180 $[M+H]^+$

FTIR (neat): 3424, 2924, 1613, 1508, 1404, 1375, 1314, 1219, 1155, 1111, 996, 916, 817, 768, 735 cm^{-1}.

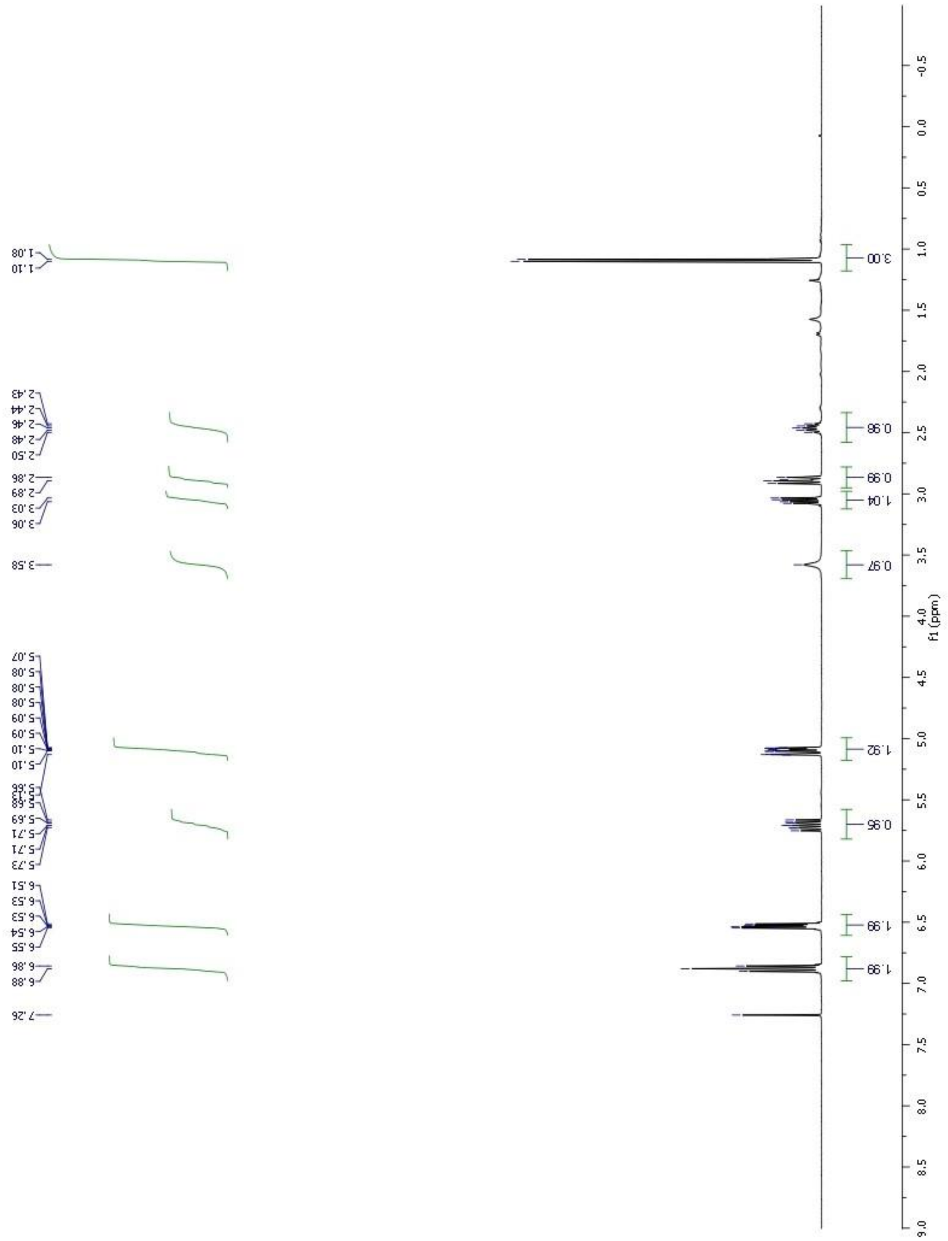
f1 (ppm)
1.08
1.10
2.43
2.44
2.46
2.48
2.50
2.86
2.89
3.03
3.06
3.58
5.07
5.10
5.66
5.69
5.71
5.71
5.73
6.51
6.53
6.53
6.53
6.54
6.55
6.86
6.88
7.26
3.00
0.98
0.99
1.04
0.97
1.92
0.96
1.99
1.99

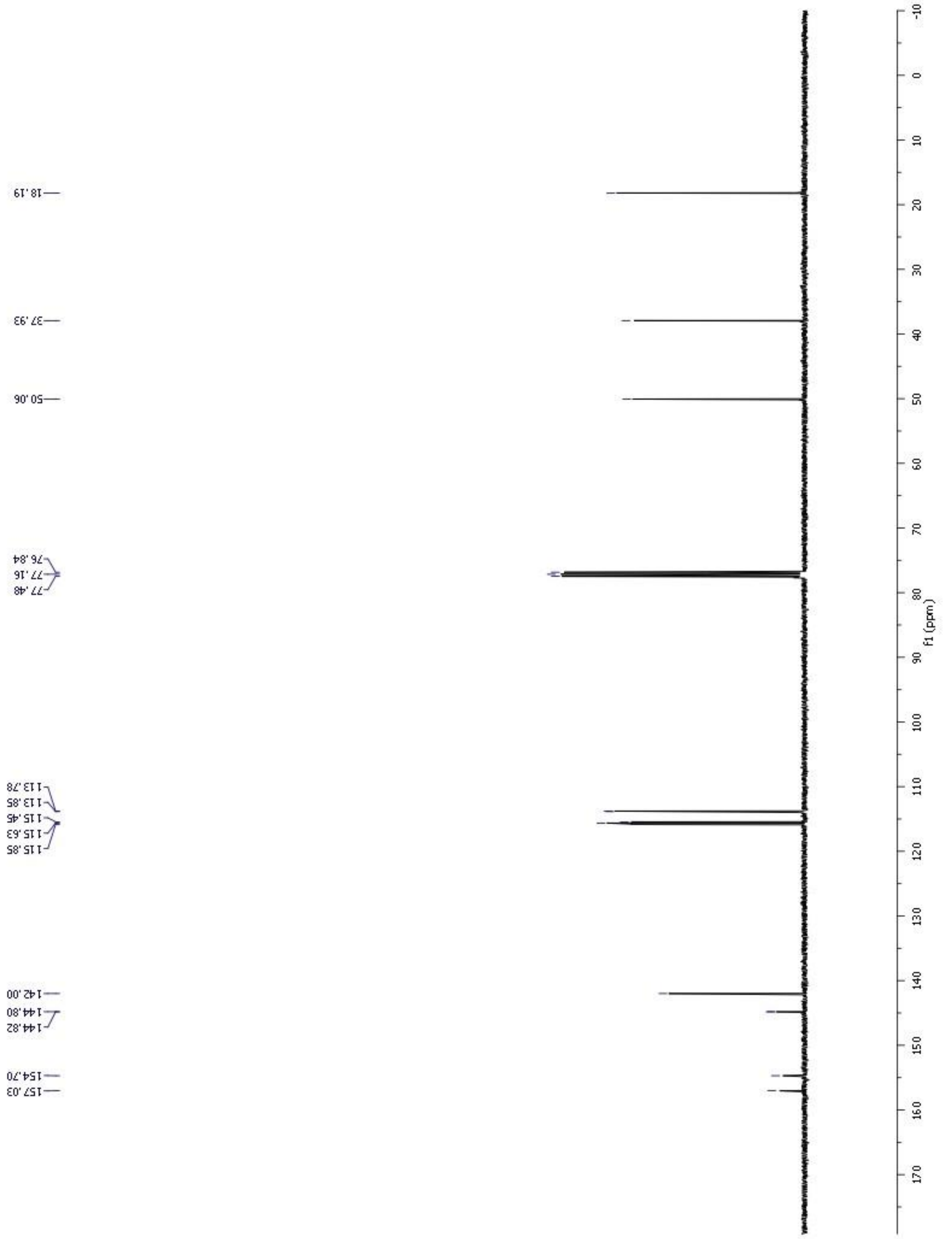
18.19
37.93
50.06
76.84
77.16
77.48
113.78
113.85
115.45
115.63
115.85
142.00
144.80
144.82
154.70
157.03
f1 (ppm)

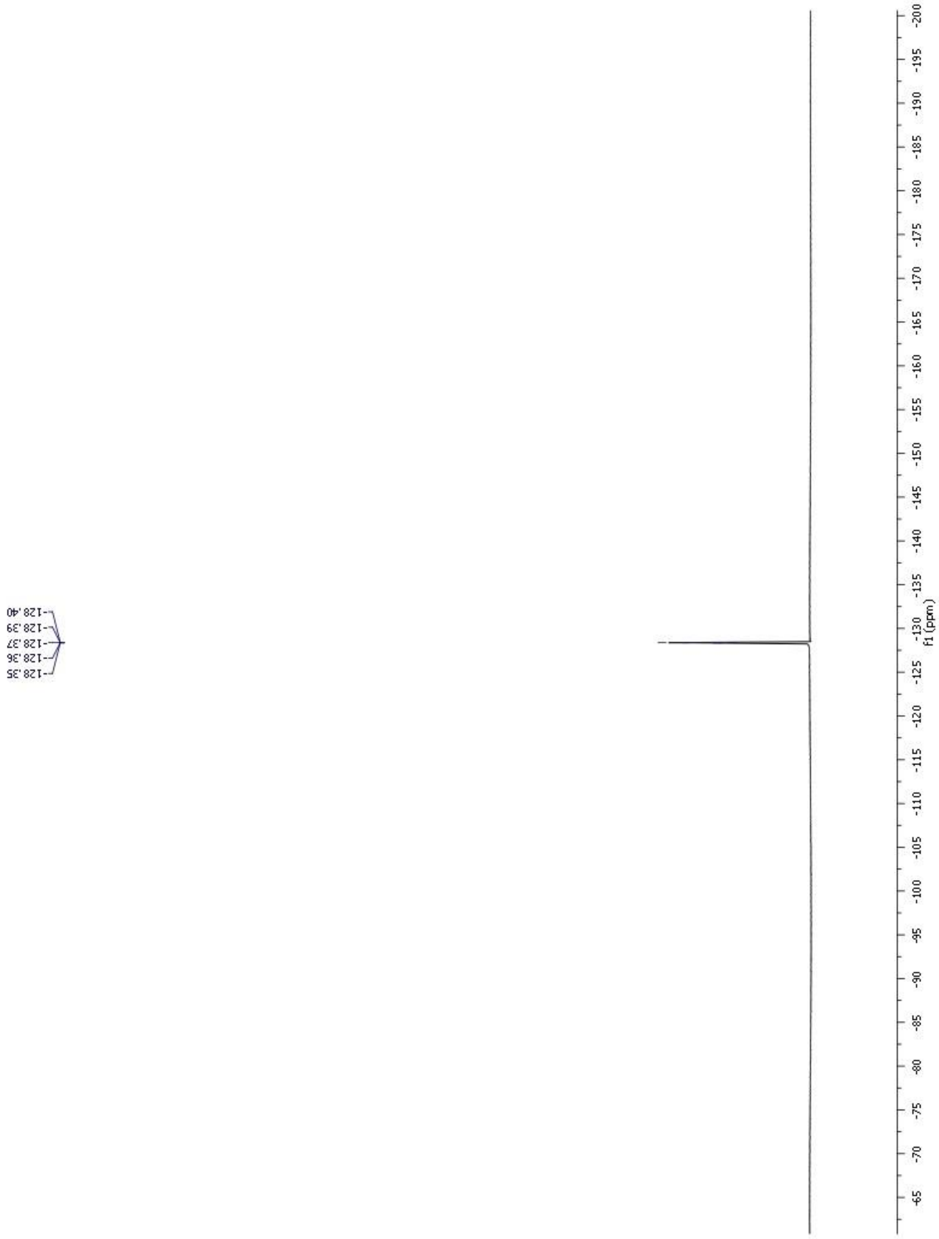
-128.40
-128.39
-128.37
-128.36
-128.35
f1 (ppm)

***N*-(2-methylbut-3-en-1-yl)-4-methoxyl-3-pyridylamine (8a)**

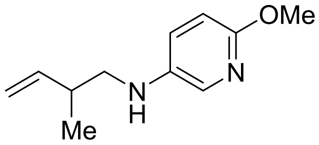

The reaction was conducted in accordance with **General Procedure C** (*via* triazine **2f**). After heating the reaction for 24 hours, the mixture was concentrated *in vacuo* and purified by flash column chromatography (SiO_2, 17% EtOAc/hexanes) to furnish the title compound (32.2 mg, 84%) as a yellow oil.

^{1}H NMR (400 MHz, $CDCl_3$): δ 7.55 (dd, *J* = 3.0, 0.5 Hz, 1H), 6.97 (dd, *J* = 8.8, 3.0 Hz, 1H), 6.61 (dd, *J* = 8.8, 0.7 Hz, 1H), 5.70 (ddd, *J* = 17.1, 10.4, 7.9 Hz, 1H), 5.20 – 4.98 (m, 2H), 3.86 (s, 3H), 3.40 (br, 1H), 3.06 (dd, *J* = 11.9, 5.4 Hz, 1H), 2.89 (dd, *J* = 11.9, 8.3 Hz, 1H), 2.59 – 2.35 (m, 1H), 1.09 (d, *J* = 6.8 Hz, 3H).

^{13}C NMR (100 MHz, $CDCl_3$): δ 157.49, 141.85, 139.38, 130.64, 126.11, 115.61, 110.87, 53.46, 50.27, 37.95, 18.15.

LRMS (ESI): m/z 193 $[M+H]^+$

FTIR (neat): 3366, 2966, 1639, 1577, 1492, 1432, 1381, 1258, 1129, 1030, 1008, 915, 821, 773, 740 cm^{-1}.

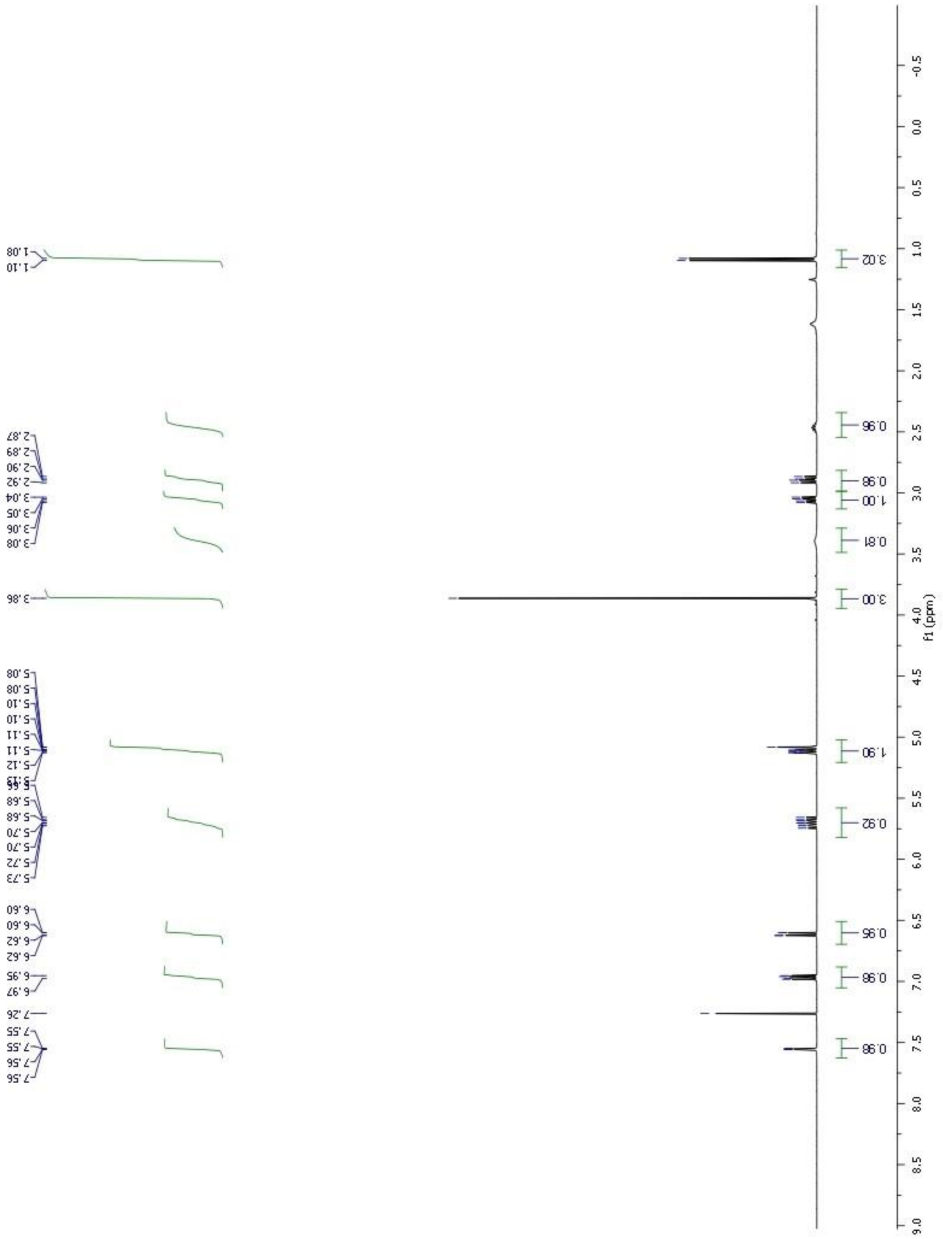
f1 (ppm)

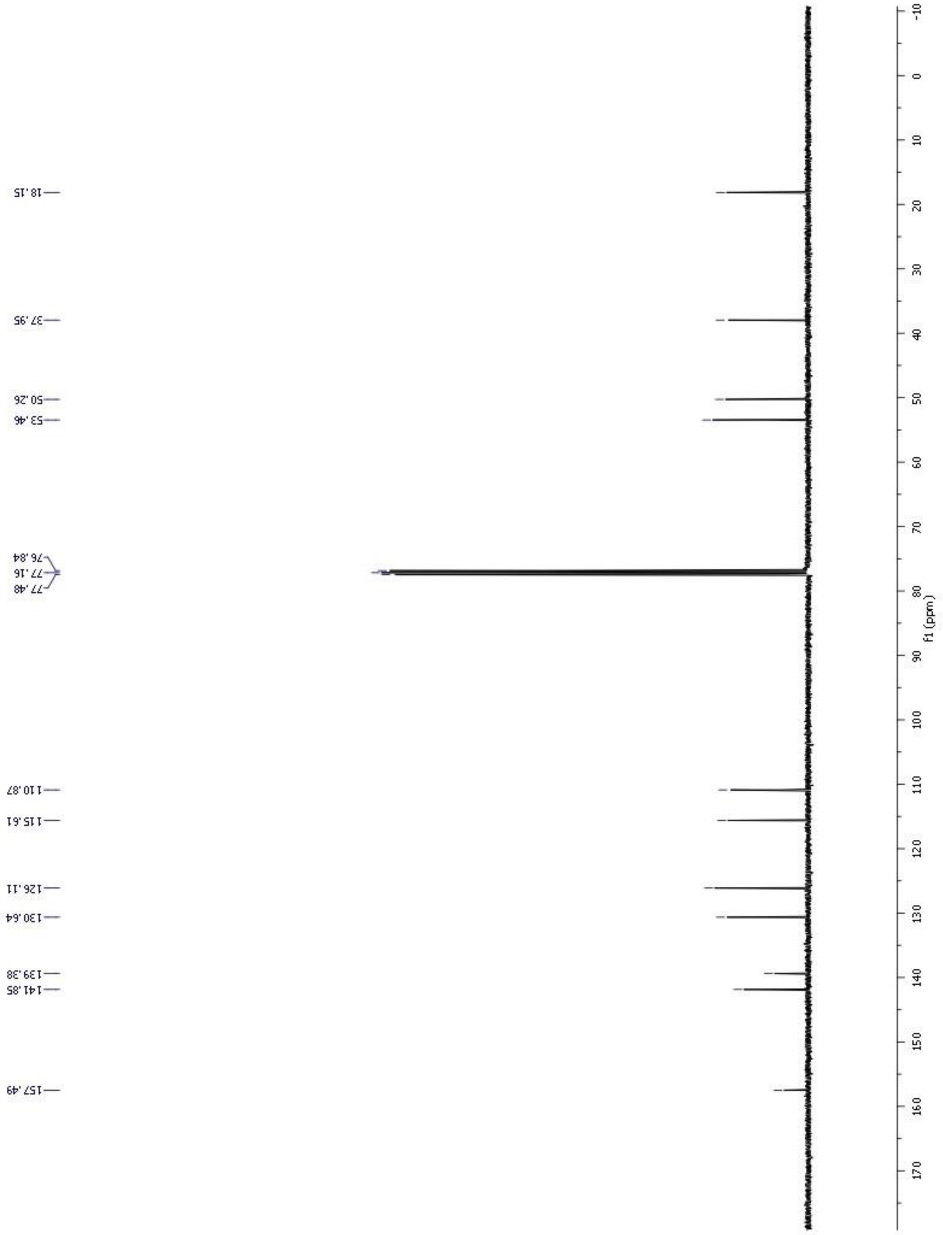
18.15
37.95
50.26
53.46
76.84
77.16
77.48
110.87
115.61
126.11
130.64
139.38
141.85
157.49
f1 (ppm)

IV. Experimental Procedure and Spectral Data for the Elaboration of Product 3i and 3b

***N*-allyl-*N*-(2-methyl-3-(4-trifluoromethylphenyl)but-3-en-1-yl)-4-methoxyaniline**

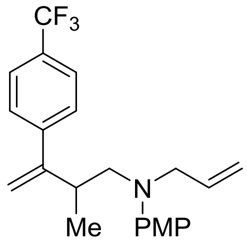

N-(2-methyl-3-(4-trifluoromethylphenyl)but-3-en-1-yl)-4-methoxyaniline **3i** (30.0 mg, 0.089 mmol, 100 mol%), K_2CO_3 (18.5 mg, 0.13 mmol, 150 mol%.), and allyl bromide (11.6 µl, 0.13 mmol, 150 mol%) were suspended in 1.0 mL of DMF and the mixture stirred overnight at room temperature. Water and Et_2O were added to the suspension, and the aqueous layer was extracted three times with ether. The combined organic layer was washed with water, brine, dried over Na_2SO_4, and evaporated to dryness. The residue was purified by flash column chromatography (SiO_2, 5% EtOAc/hexanes) to furnish the title compound as a yellow oil (25.2 mg, 75%).

^{1}H NMR (400 MHz, $CDCl_3$): δ 7.55 (dd, *J* = 8.7, 0.6 Hz, 2H), 7.40 (dd, *J* = 8.7, 0.7 Hz, 2H), 6.78 (d, *J* = 9.2 Hz, 2H), 6.60 (d, *J* = 9.2 Hz, 2H), 5.75 (ddt, *J* = 17.0, 10.5, 5.2 Hz, 1H), 5.32 (m, 1H), 5.21 (m, 1H), 5.17 – 4.94 (m, 2H), 3.91 – 3.68 (m, 5H), 3.43 – 3.26 (m, 1H), 3.19 – 2.97 (m, 2H), 1.20 (d, *J* = 6.4 Hz, 3H).

^{13}C NMR (100 MHz, $CHCl_3$): δ 152.39, 151.75, 146.48, 143.16, 134.61, 129.43 (q, *J* = 32.5 Hz), 127.05, 125.27 (q, *J* = 3.8 Hz), 124.37 (q, *J* = 270.6 Hz), 116.40, 115.16, 114.75, 113.95, 57.92, 55.87, 55.42, 37.01, 18.52.

^{19}F NMR (376 MHz, $CDCl_3$): δ -62.46.

LRMS (ESI): m/z 376 $[M+H]^+$

FTIR (neat): 2934, 1615, 1511, 1463, 1405, 1324, 1241, 1164, 1122, 1065, 1040, 1015, 908, 850, 812, 738 cm^{-1}.

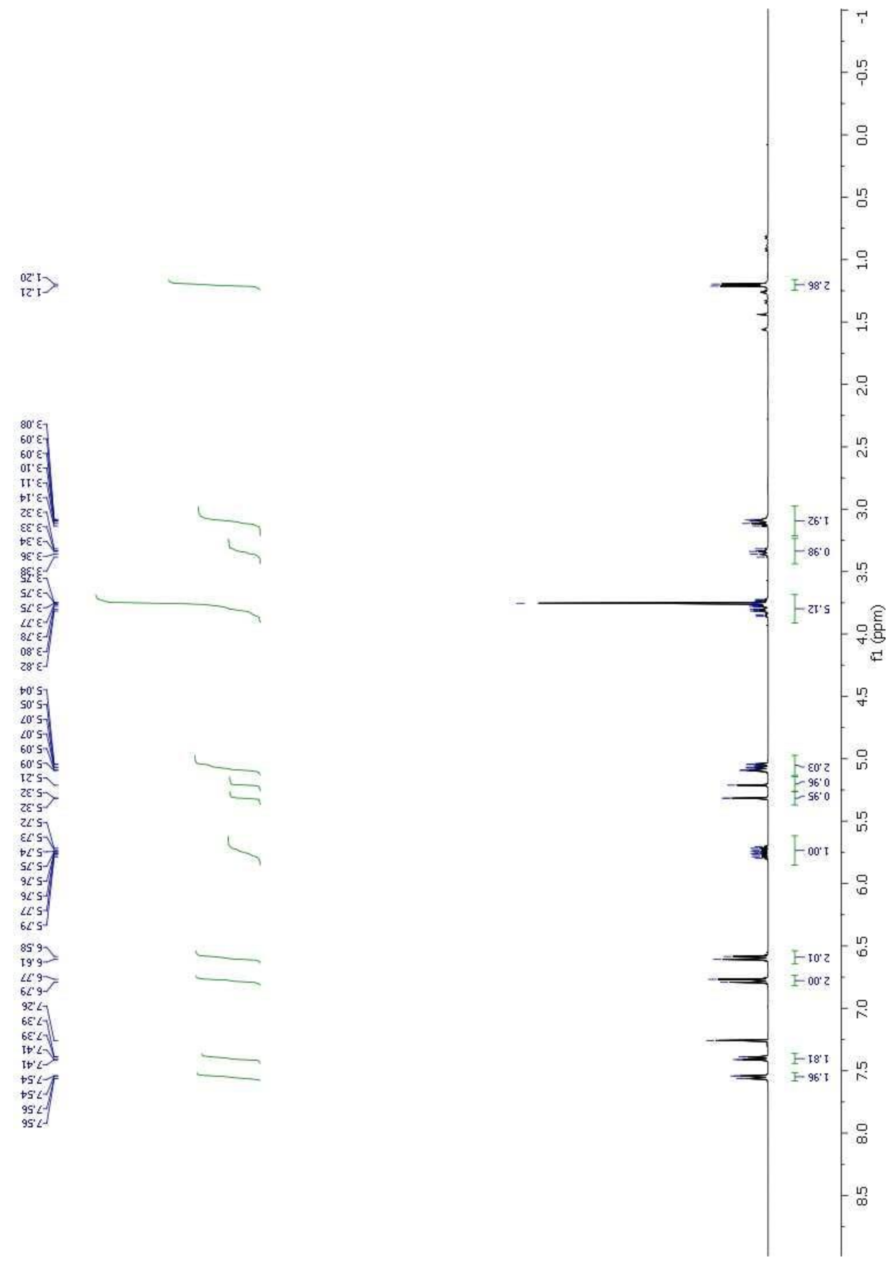

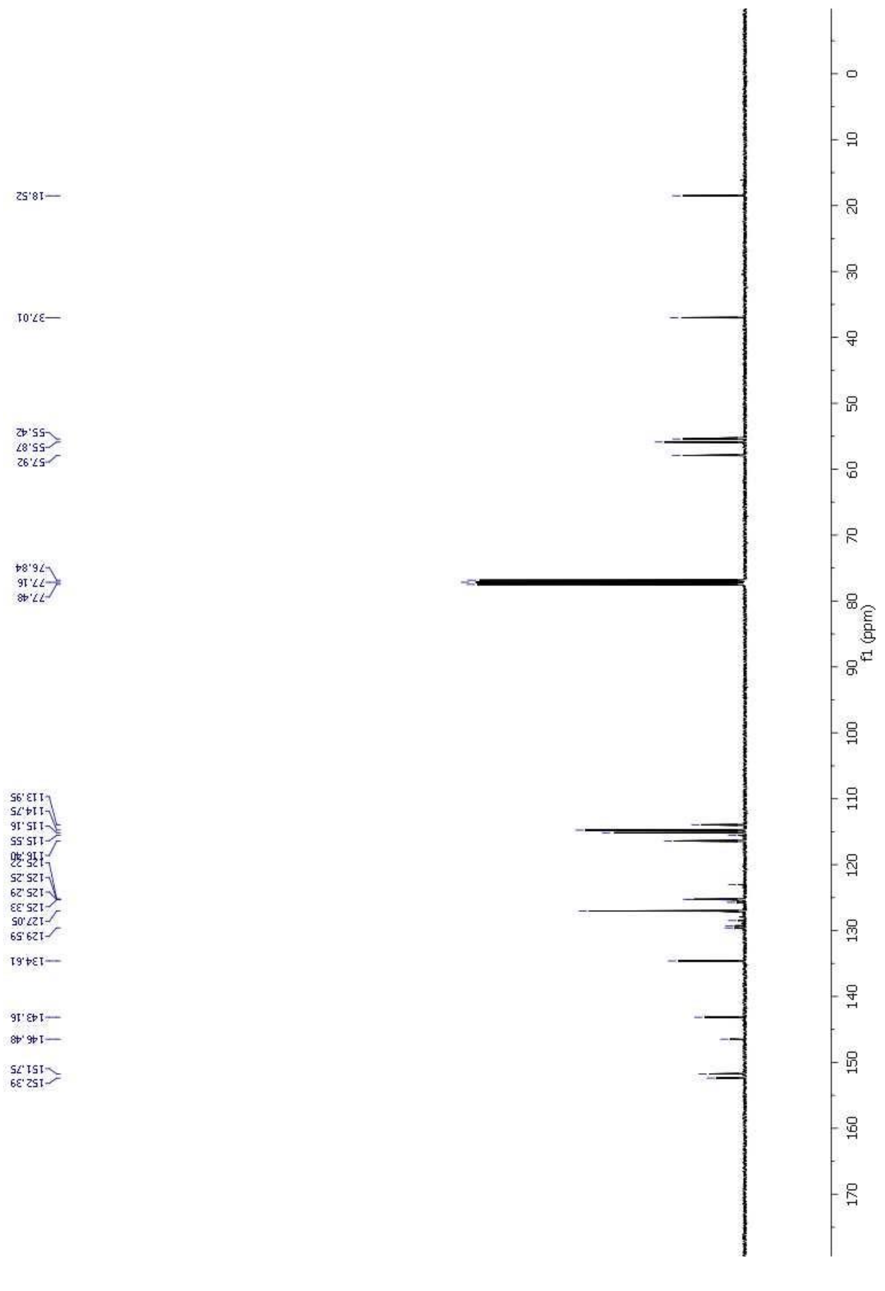
18.52
37.01
55.42
55.87
57.92
76.84
77.16
77.48
113.95
114.75
115.16
115.55
116.40
125.22
125.25
125.29
125.33
127.05
129.59
134.61
143.16
146.48
151.75
152.39
f1 (ppm)
0
10
20
30
40
50
60
70
80
90
100
110
120
130
140
150
160
170

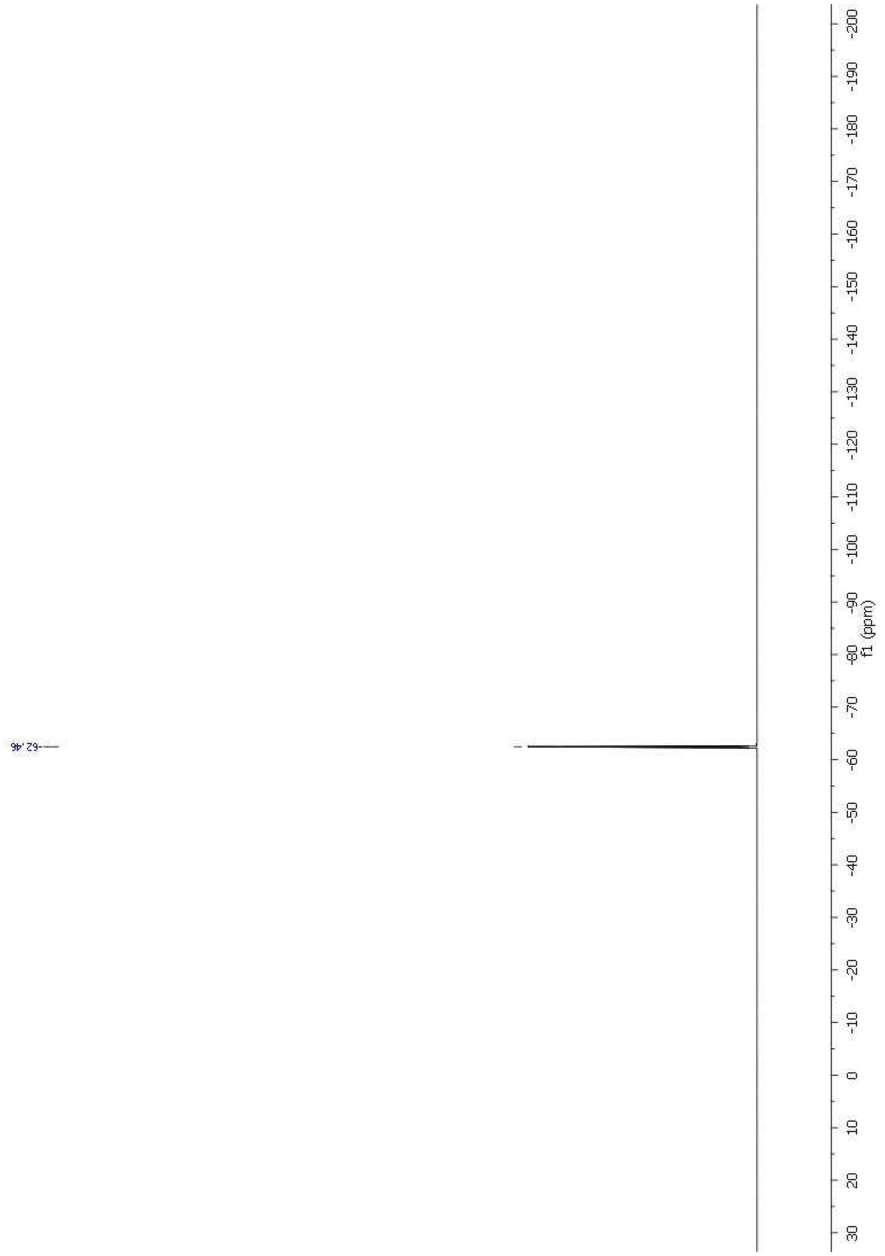

-62.46
f1 (ppm)

N-(4-methoxyphenyl)-3-trifluorophenyl-4-methylpiperidin-2-en (4i)

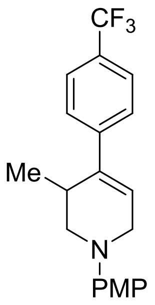

N-allyl-*N*-(2-methyl-3-(4-trifluoromethylphenyl)but-3-en-1-yl)-4-methoxyaniline (25.2 mg, 0.067 mmol, 100 mol%) was dissolved in dry degassed CH_2Cl_2 (2.0 mL) in a round-bottom flask under argon. The Grubb's II catalyst (2.8 mg, 0.0033 mmol, 5 mol%) was added, and the solution was stirred at 40 °C for 16 hours. The solvent was evaporated, and the crude residue was purified by flash column chromatography (SiO_2, 5% EtOAc/hexanes) to furnish the title compound (16.8 mg, 72% yield) as a white solid.

^{1}H NMR (400 MHz, $CDCl_3$): δ 7.60 (d, *J* = 8.1 Hz, 2H), 7.48 (d, *J* = 8.1 Hz, 2H), 6.94 (d, *J* = 9.2 Hz, 2H), 6.88 (d, *J* = 9.3 Hz, 2H), 6.09 (t, *J* = 3.3 Hz, 1H), 3.90 – 3.61 (m, 5H), 3.31 (d, *J* = 4.1 Hz, 2H), 3.09 – 2.92 (m, 1H), 1.12 (d, *J* = 6.9 Hz, 3H).

^{13}C NMR (100 MHz, $CHCl_3$): δ 153.42, 145.57, 144.13, 140.47, 129.15 (q, *J* = 32.5 Hz), 126.36, 125.47 (q, *J* = 3.8 Hz), 124.41 (q, *J* = 270.6 Hz), 124.06, 117.45, 114.73, 55.83, 54.90, 50.08, 32.52, 18.43.

^{19}F NMR (376 MHz, $CDCl_3$): δ -62.42.

LRMS (ESI): m/z 348 $[M+H]^+$

FTIR (neat): 2918, 1831, 1610, 1510, 1463, 1412, 1389, 1323, 1286, 1273, 1245, 1166, 1116, 1069, 1039, 1012, 814, cm^{-1}.

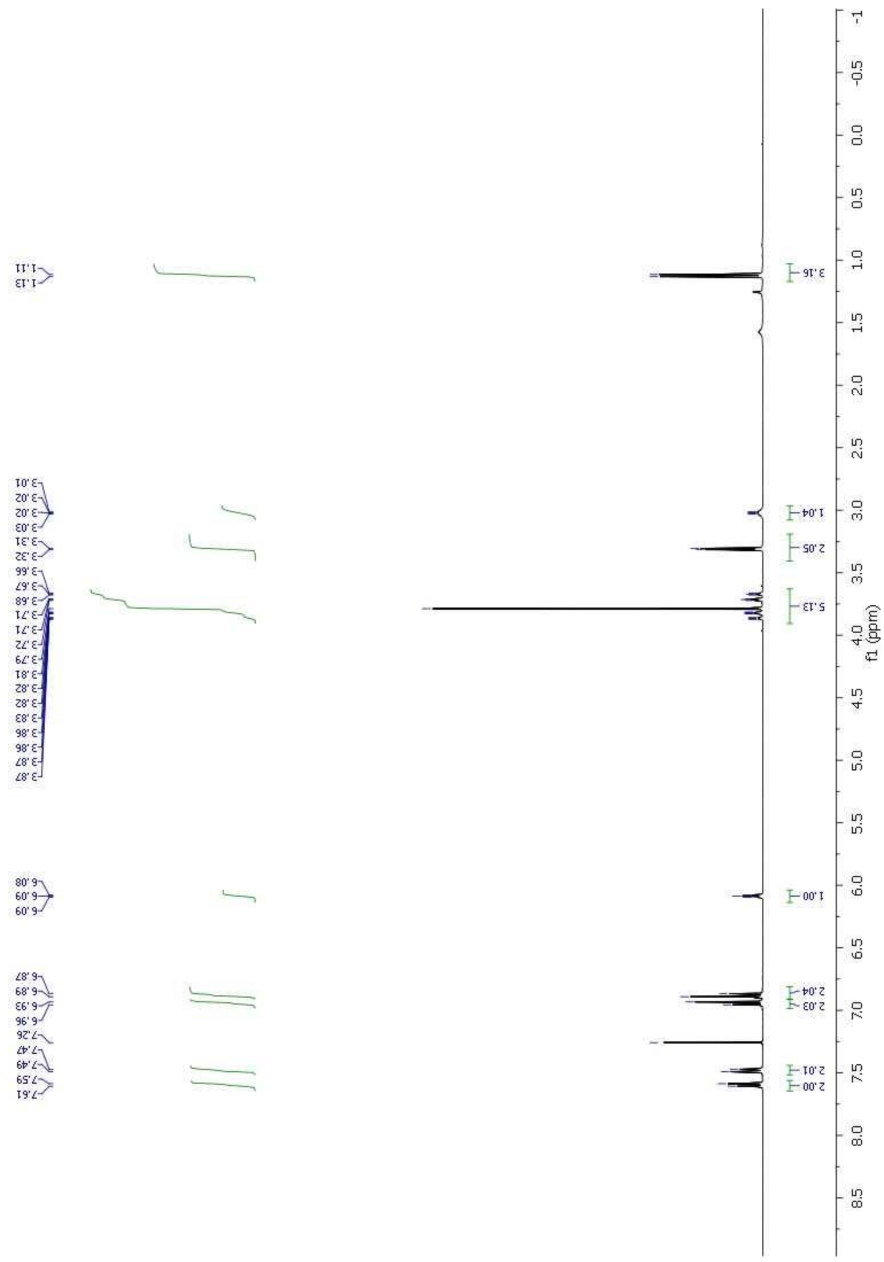
f1 (ppm)
3.16
1.04
2.05
5.13
1.00
2.04
2.03
2.01
2.00

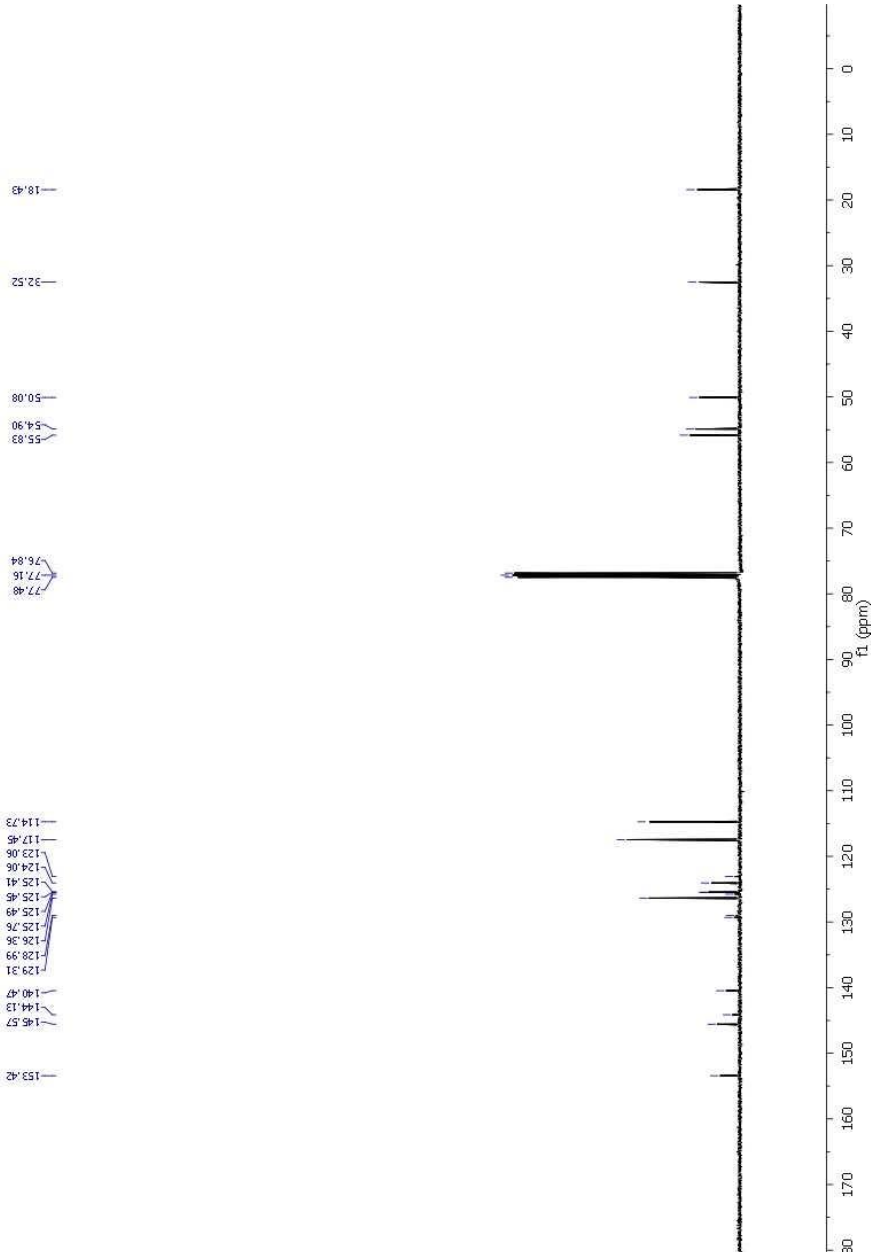
18.43
32.52
50.08
54.90
55.83
76.84
77.16
77.48
114.73
117.46
123.06
124.06
125.41
125.45
125.49
125.76
126.36
128.99
129.31
140.47
144.13
145.57
153.42
f1 (ppm)

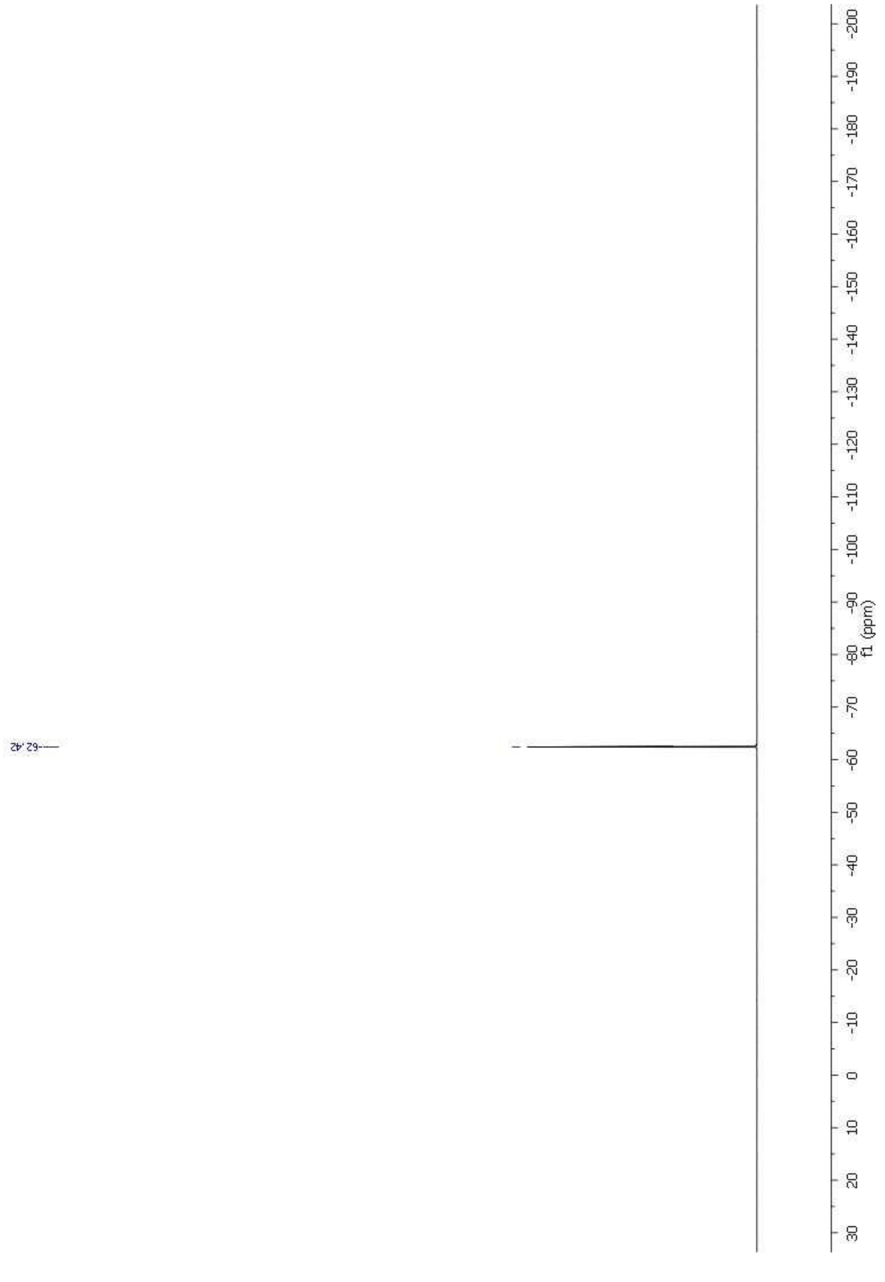
-62.42
f1 (ppm)

2-(4-methoxyphenyl)-4,5-dimethyl-6-oxa-2-azabicyclo[3.2.1]octan-7-one (4b)

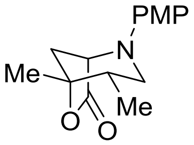

N-(2,3-dimethylbut-3-en-1-yl)-4-methoxyaniline **3b** (30.0 mg, 0.146 mmol, 100 mol%) was dissolved in MeCN/H_2O (1:1, 2.0 mL) and glyoxalic acid monohydrate (15.0 mg, 0.161 mmol, 110 mol%) was added. The solution was stirred for 24 hours at room temperature. H_2O was added and the aqueous layer extracted with CH_2Cl_2. The combined organic layers were dried over Na2SO4.The solvent was removed under reduced pressure, and the residue was purified by flash column chromatography (SiO_2, 17% EtOAc/hexanes) to furnish the title compound (30.5 mg, 80%).

^{1}H NMR (400 MHz, $CDCl_3$): δ 7.02-7.06 (m, 2H), 6.82-6.86 (m, 2H), 3.90 (s, 1H), 3.77 (s, 3H), 3.42-3.47 (m, 1H), 3.06-3.14 (m, 1H), 2.23 (q, *J* = 7.0 Hz, 1H), 1.99-2.03 (m, 2H), 1.43 (s, 3H), 1.06 (d, *J* = 7.0 Hz, 3H).

^{13}C NMR (100 MHz, $CHCl_3$): δ 173.9, 154.8, 143.6, 120.2, 114.6, 85.8, 68.7, 55.6, 46.1, 45.6, 36.8, 20.6, 11.9.

LRMS (ESI): m/z 262 $[M+H]^+$

FTIR (neat): 2930, 2834, 1770, 1510, 1454, 1383, 1240, 1209, 1155, 1122, 1094, 1060, 1036, 976, 934, 917, 900, 877, 825, 800 cm^{-1}.

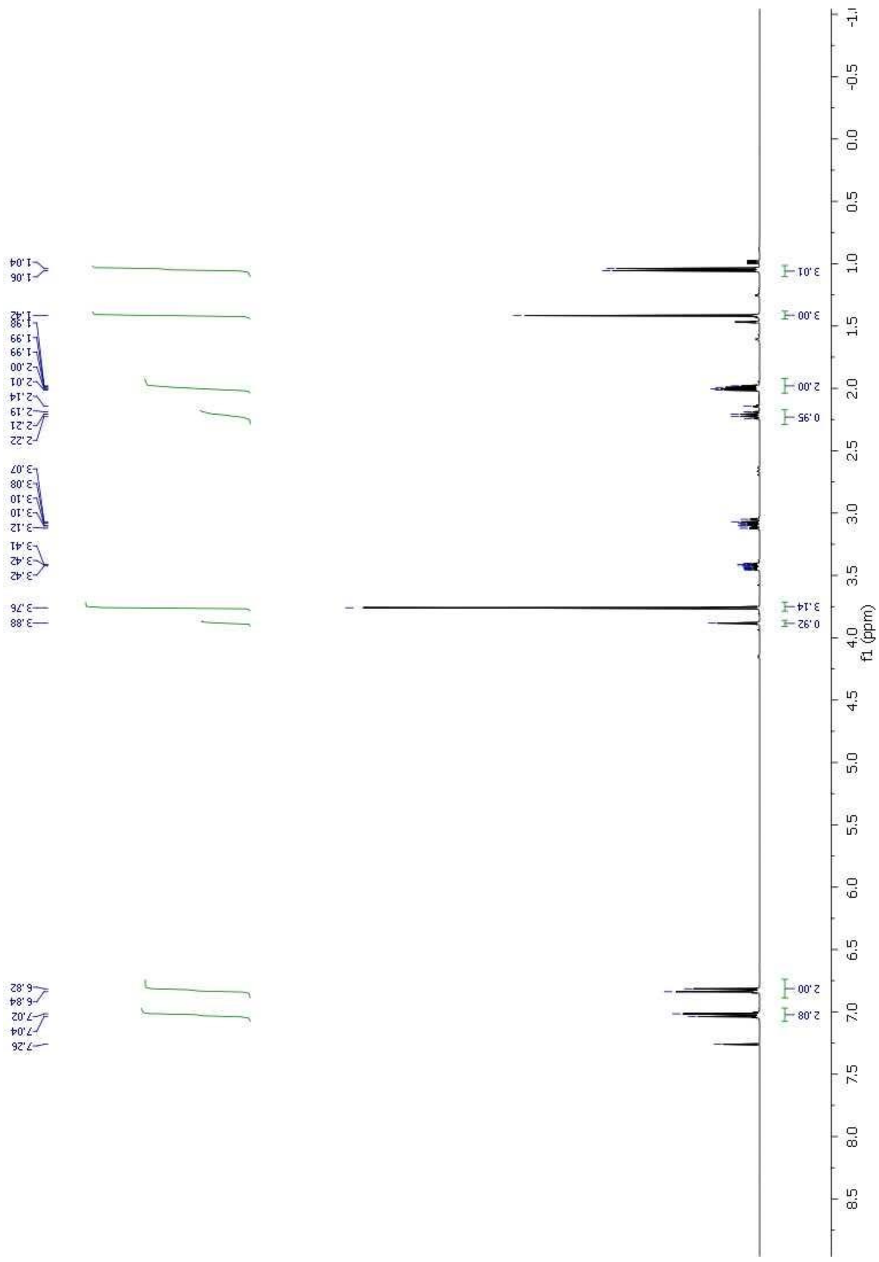
f1 (ppm)

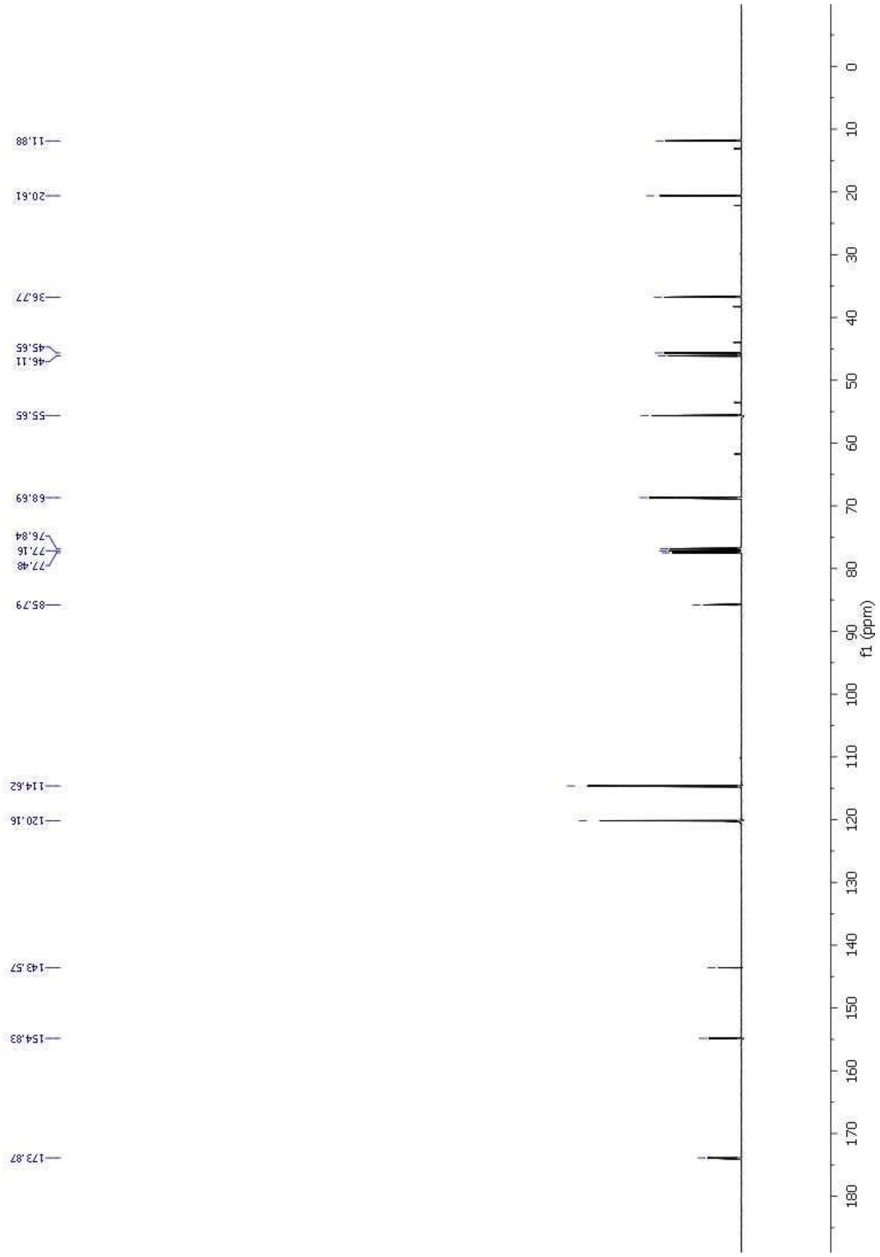
11.88
20.61
36.77
45.65
46.11
55.65
68.69
76.84
77.16
77.48
85.79
114.62
120.16
143.57
154.83
173.87
f1 (ppm)

V. Experimental Procedures and Spectral Data Adducts *deuterio*-3b

***N*-(2,3-dimethylbut-3-en-1-yl)-4-methoxyaniline (*deuterio*-3b)**

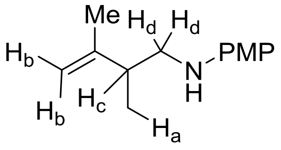

To an oven-dried pressure tube equipped with magnetic stir bar was added $RuHCl(CO)(PPh_3)_3$ (9.5 mg, 0.010 mmol, 5 mol%), bis(dicyclohexylphosphino)methane (dCypm) (4.1 mg, 0.010 mmol, 5 mol%), and *deuterio*-triazine **2a** (0.067 mmol, 33.3 mol% (0.200 mmol ,100 mol% of imine)). The tube was sealed with a rubber septum, purged with argon, and xylene (0.5 M with respect to formimine), diene **1b** (0.400 mmol, 200 mol%), and isopropanol (61 µL, 0.800 mmol, 400 mol%) were added. The rubber septum was quickly replaced with a screw cap and the reaction was heated to 140 °C for 24 hours. The reaction mixture was allowed to cool to room temperature, concentrated *in vacuo*, and purified by flash column chromatography (SiO_2, 6% EtOAc/hexanes) to furnish *deuterio*-**3b** (30.4 mg, 74%).

^{1}H NMR (400 MHz, $CDCl_3$): δ 6.78 (d, *J* = 9.0 Hz, 2H), 6.57 (d, *J* = 9.0 Hz, 2H), 4.87 – 4.84 (m, 1H), 4.83 – 4.80 (m, 1H), 3.75 (s, 3H), 3.36 (br, 1H), 2.49 (q, *J* = 6.9 Hz, 1H), 1.69 (dd, *J* = 1.3, 0.9 Hz, 3H), 1.10 (d, *J* = 6.9 Hz, 3H).

^{2}H NMR (77 MHz, $CHCl_3$): δ 3.01, 2.96.

HRMS (ESI): Calcd. For $C_{13}H_{17}D_2NO$ $[M+H]^+$ 208.1665, Found: 208.1665

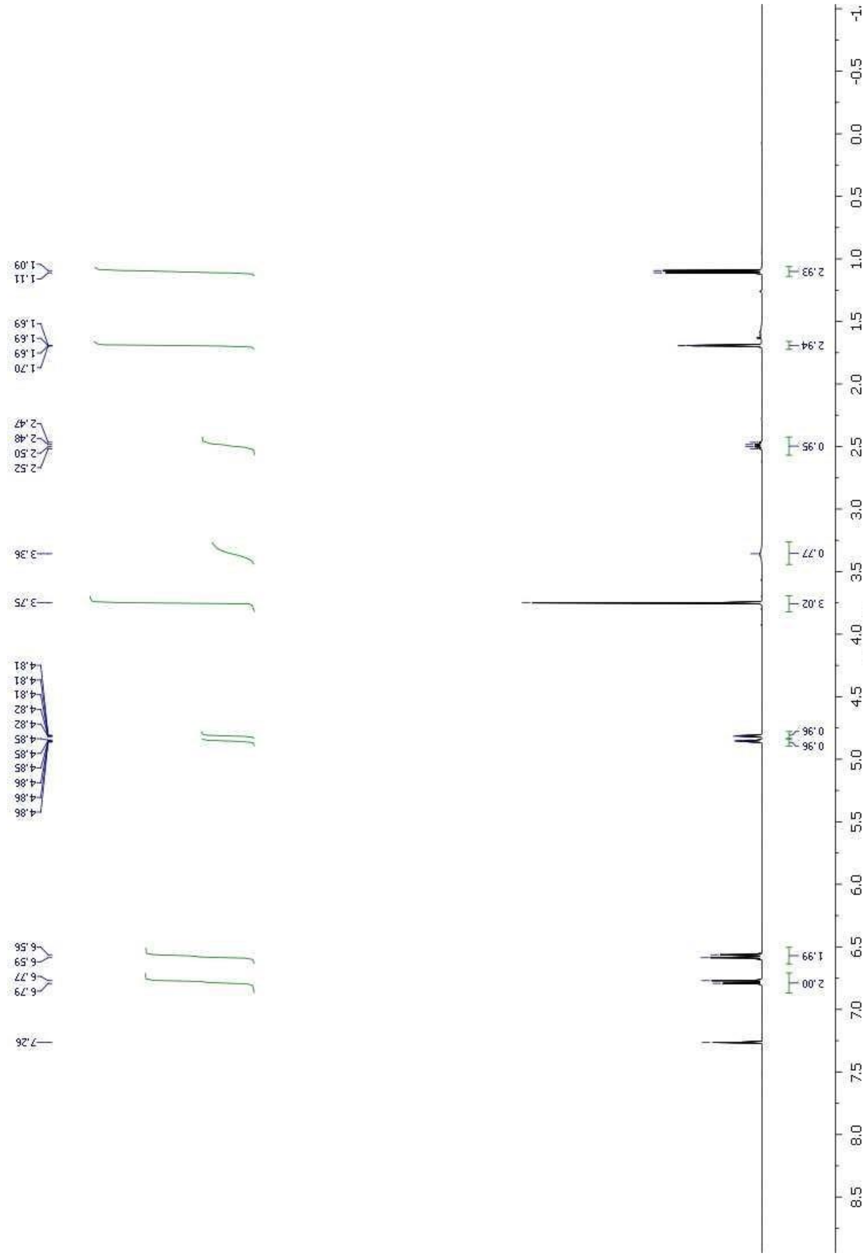

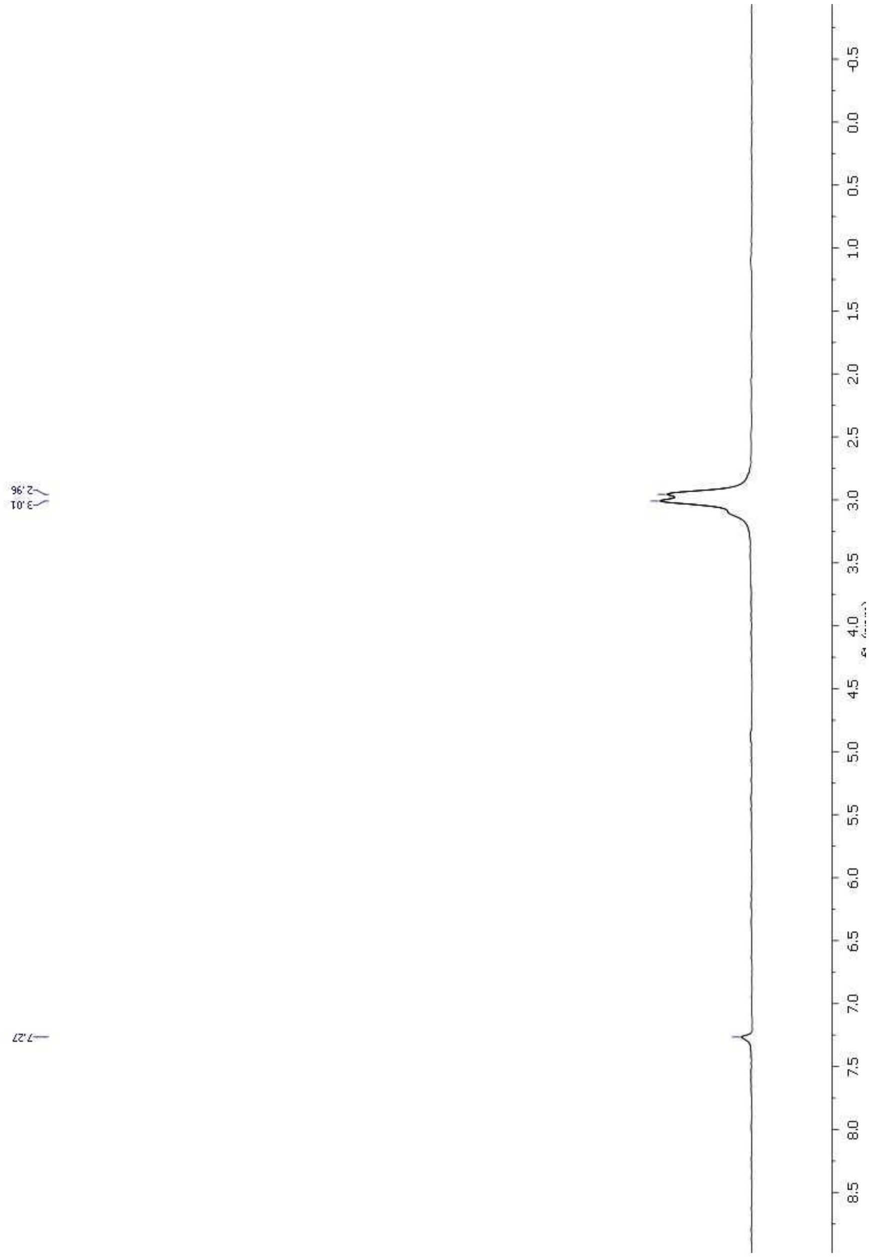

2.96
3.01
7.27
-0.5
0.0
0.5
1.0
1.5
2.0
2.5
3.0
3.5
4.0
4.5
5.0
5.5
6.0
6.5
7.0
7.5
8.0
8.5

***N*-(2,3-dimethylbut-3-en-1-yl)-4-methoxyaniline (*deuterio*-3b)**

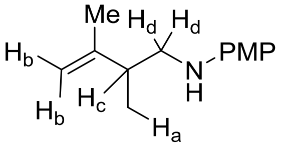

To an oven-dried pressure tube equipped with magnetic stir bar was added $RuHCl(CO)(PPh_3)_3$ (9.5 mg, 0.010 mmol, 5 mol%), bis(dicyclohexylphosphino)methane (dCypm) (4.1 mg, 0.010 mmol, 5 mol%), and triazine **2a** (0.067 mmol, 33.3 mol%, (0.200 mmol, 100 mol% of imine)). The tube was sealed with a rubber septum, purged with argon, and xylene (0.5 M with respect to formimine), diene **1b** (0.800 mmol, 400 mol%), and d_8-isopropanol (61 µL, 0.800 mmol, 400 mol%) were added. The rubber septum was quickly replaced with a screw cap and the reaction was heated to 140 °C for 24 hours. The reaction mixture was allowed to cool to room temperature, concentrated *in vacuo*, and purified by flash column chromatography (SiO_2, 6% EtOAc/hexanes) to furnish *deuterio*-**3b** (30.7 mg, 75%).

^{1}H NMR (400 MHz, $CDCl_3$): δ 7.44 – 7.27 (m, 5H), 6.76 (d, *J* = 8.9 Hz, 2H), 6.55 (d, *J* = 8.9 Hz, 2H), 6.00 – 5.80 (m, 0.29H), 5.26 – 5.03 (m, 0.88H), 4.52 (s, 2H), 3.75 (s, 3H), 3.63 (br, 1H), 3.40 (dd, *J* = 26.0, 8.9 Hz, 2H), 3.10 (s, 2H), 1.14 (s, 3H).

^{2}H NMR (77 MHz, $CHCl_3$): δ 4.86, 2.49, 1.11.

HRMS (ESI): Calcd. For $C_{13}H_{18}DNO$ $[M+H]^+$ 207.1602, Found: 207.1601

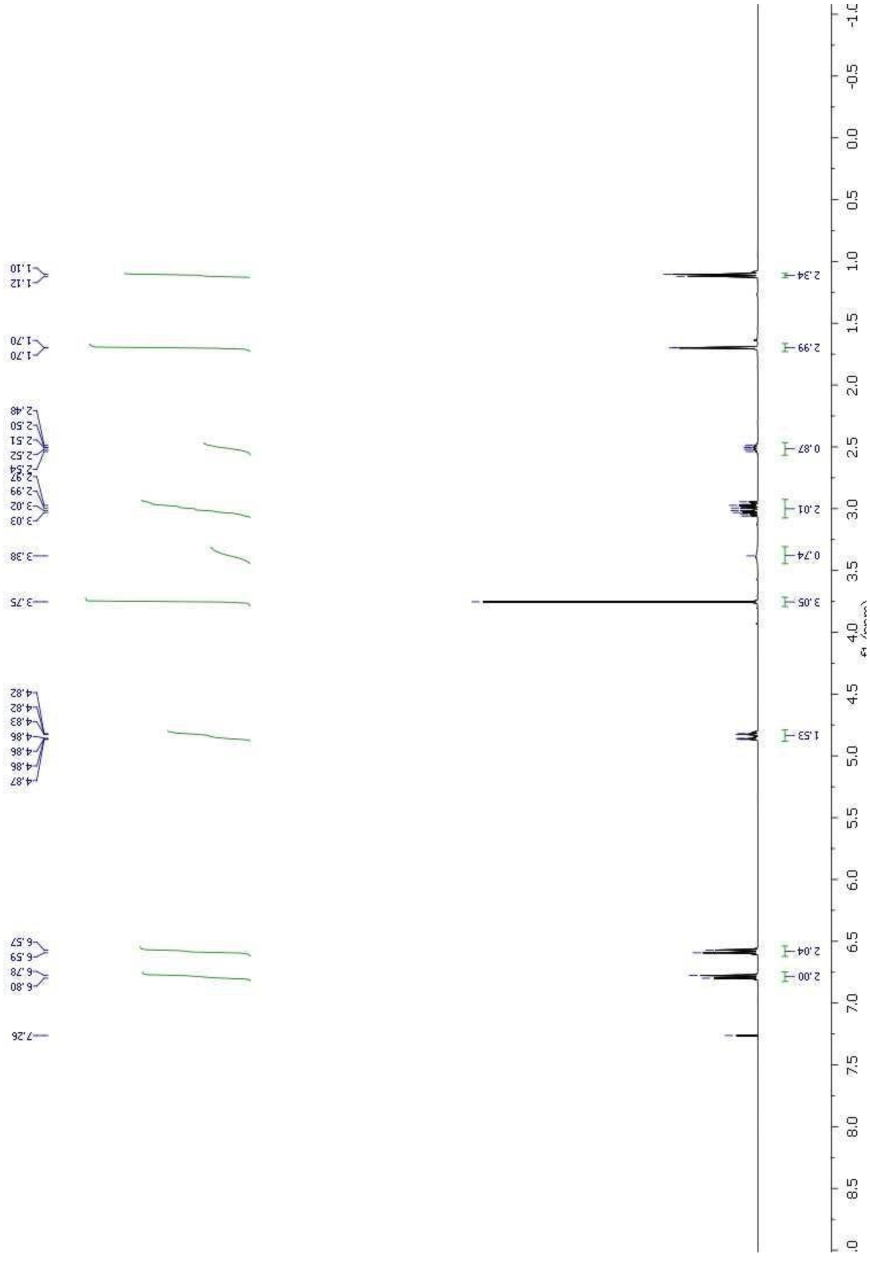
1.10
1.12
1.70
1.70
2.48
2.50
2.51
2.52
2.54
2.97
2.99
3.02
3.03
3.38
3.75
4.82
4.82
4.83
4.86
4.86
4.86
4.87
6.57
6.59
6.78
6.80
7.26
2.34
2.99
0.87
2.01
0.74
3.05
1.53
2.04
2.00
f1 (ppm)

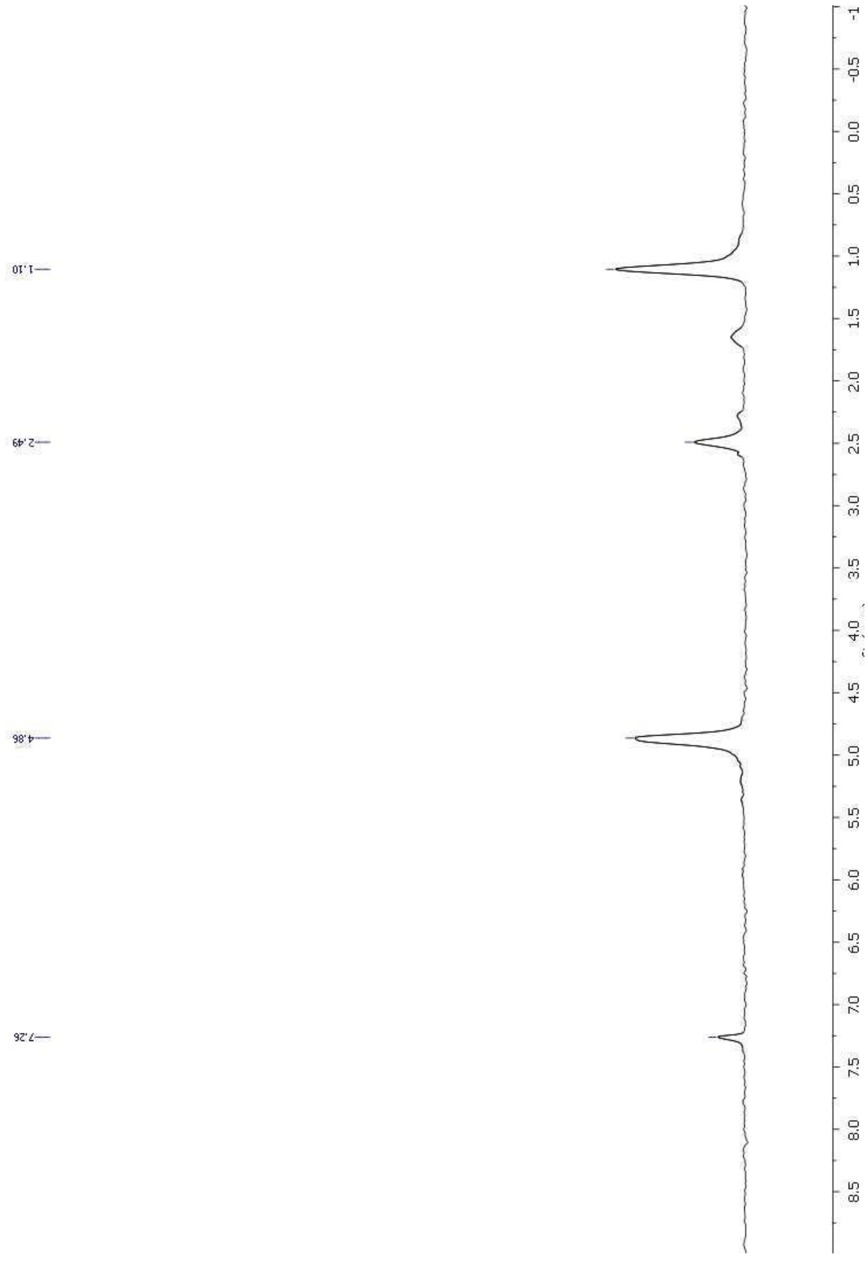
1.10
2.49
4.86
7.26

VI. Experimental Procedure and Spectral Data Adducts (*R*)-5a and (*R*)-5b

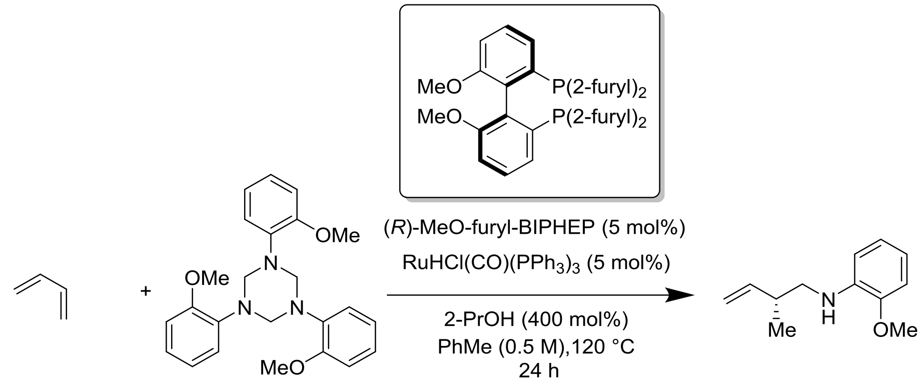

1a (400 mol%)

2c (33.3 mol% 100 mol% imine)

(*R*)-**5a** 49% yield, 94:6 e.r.

Experimental Procedure for the enantioselective coupling of triazine 2c to diene 1a

To an oven-dried pressure tube equipped with magnetic stir bar was added RuHCl(CO)(PPh_3)$_3$ (9.5 mg, 0.010 mmol, 5 mol%), (*R*)-(+)-2,2'-Bis(di-2-furanylphosphino)-6,6'-dimethoxy-1,1'-biphenyl ((*R*)-MeO-furyl-BIPHEP) (4.1 mg, 0.010 mmol, 5 mol%), and triazine **2c** (27.0 mg, 0.067 mmol, 33.3 mo% (0.200 mmol, 100 mol% of imine)). The tube was sealed with a rubber septum, purged with argon, and toluene (0.4 mL, 0.5 M with respect to formimine), 1,3-butadiene **1a** (70 µL, 0.800 mmol, 400 mol%), and isopropanol (61 µL, 0.800 mmol, 400 mol%) were added. The rubber septum was quickly replaced with a screw cap and the reaction was heated to 120 °C for 24 hours. The reaction mixture was allowed to cool to room temperature, concentrated *in vacuo*, and purified by flash column chromatography (SiO_2, 5% EtOAc/hexanes) to furnish the title compounds (*R*)-**5a** (18.7 mg, 49%, 94:6 er). Spectral Data (^{1}H NMR, ^{13}C NMR, LRMS and IR) are consistent with **5a** (see S36).

HPLC (Chiralcel OJ-H column, hexanes:*i*-PrOH = 95:5, 0.5 mL/min, 210 nm), er = 94:6.

$[\alpha]^{30}_D$ = - 101.7 (c = 1.0, $CHCl_3$)

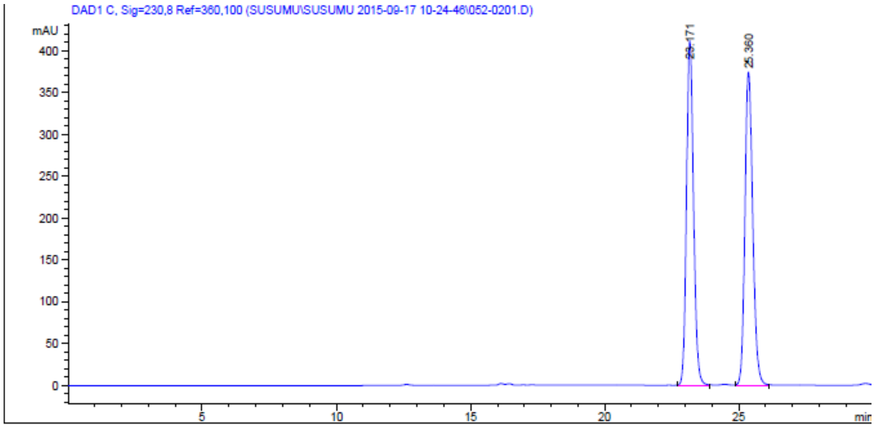

Signal 1: DAD1 C, Sig=230,8 Ref=360,100

Peak #	RetTime [min]	Type	Width [min]	Area [mAU*s]	Height [mAU]	Area %
1	23.171	BB	0.2787	7438.81494	411.84775	49.7495
2	25.360	BB	0.3082	7513.72168	374.32516	50.2505
Totals :				1.49525e4	786.17291	

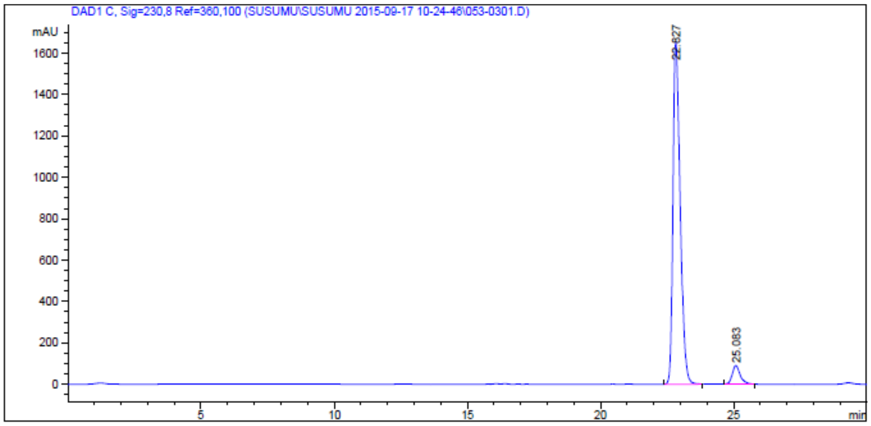

Signal 1: DAD1 C, Sig=230,8 Ref=360,100

Peak #	RetTime [min]	Type	Width [min]	Area [mAU*s]	Height [mAU]	Area %
1	22.827	BB	0.2879	3.12022e4	1655.40845	94.2530
2	25.083	BB	0.3212	1902.54163	89.80824	5.7470
Totals :				3.31048e4	1745.21669	

Experimental Procedures and Spectral Data Adducts (*R*)-5b

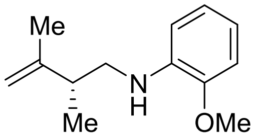

<u>Experimental Procedure for the enantioselective coupling of triazine 2c to diene 1b</u>

To an oven-dried pressure tube equipped with magnetic stir bar was added $RuHCl(CO)(PPh_3)_3$ (9.5 mg, 0.010 mmol, 5 mol%), (*R*)-(+)-2,2'-Bis(di-2-furanylphosphino)-6,6'-dimethoxy-1,1'-biphenyl ((*R*)-MeO-furyl-BIPHEP) (4.1 mg, 0.010 mmol, 5 mol%), and triazine **2c** (27.0 mg, 0.067 mmol, 33.3 mo% (0.200 mmol, 100 mol% of imine)). The tube was sealed with a rubber septum, purged with argon, and xylene (0.4 mL, 0.5 M with respect to formimine), isoprene **1b** (80 µL, 0.800 mmol, 400 mol%), and isopropanol (61 µL, 0.800 mmol, 400 mol%) were added. The rubber septum was quickly replaced with a screw cap and the reaction was heated to 140 °C for 24 hours. The reaction mixture was allowed to cool to room temperature, concentrated *in vacuo*, and purified by flash column chromatography (SiO_2, 5% EtOAc/hexanes) to furnish the title compounds (*R*)-**5b** (22.1 mg, 54%, 93:7 er).

<u>^{1}H NMR</u> (400 MHz, $CDCl_3$): δ 6.88 (td, *J* = 7.6, 1.4 Hz, 1H), 6.77 (dd, *J* = 7.9, 1.4 Hz, 1H), 6.69 – 6.59 (m, 2H), 4.91 – 4.81 (m, 2H), 4.25 (br, 1H), 3.84 (s, 3H), 3.07 (d, *J* = 7.1 Hz, 2H), 2.56 (h, *J* = 6.9 Hz, 1H), 1.72 (dd, *J* = 1.4, 0.9 Hz, 3H), 1.14 (d, J = 6.9 Hz, 3H).

<u>^{13}C NMR</u> (100 MHz, $CHCl_3$): δ 147.72, 146.96, 138.49, 121.39, 116.23, 111.54, 109.89, 109.56, 55.60, 47.58, 40.84, 19.01, 17.70.

<u>LRMS</u> (ESI): m/z 206 $[M+H]^+$

<u>FTIR</u> (neat): 3067, 2961, 2833, 1643, 1602, 1513, 1455, 1429, 1375, 1343, 1304, 1246, 1220, 1176, 1146, 1128, 1047, 1029, 892, 733 cm^{-1}.

<u>HPLC</u> (Chiralcel OJ-H column, hexanes:*i*-PrOH = 95:5, 0.5 mL/min, 235 nm), er = 93:7.

$[\alpha]^{30}_D$ = - 94.0 (c = 1.0, $CHCl_3$)

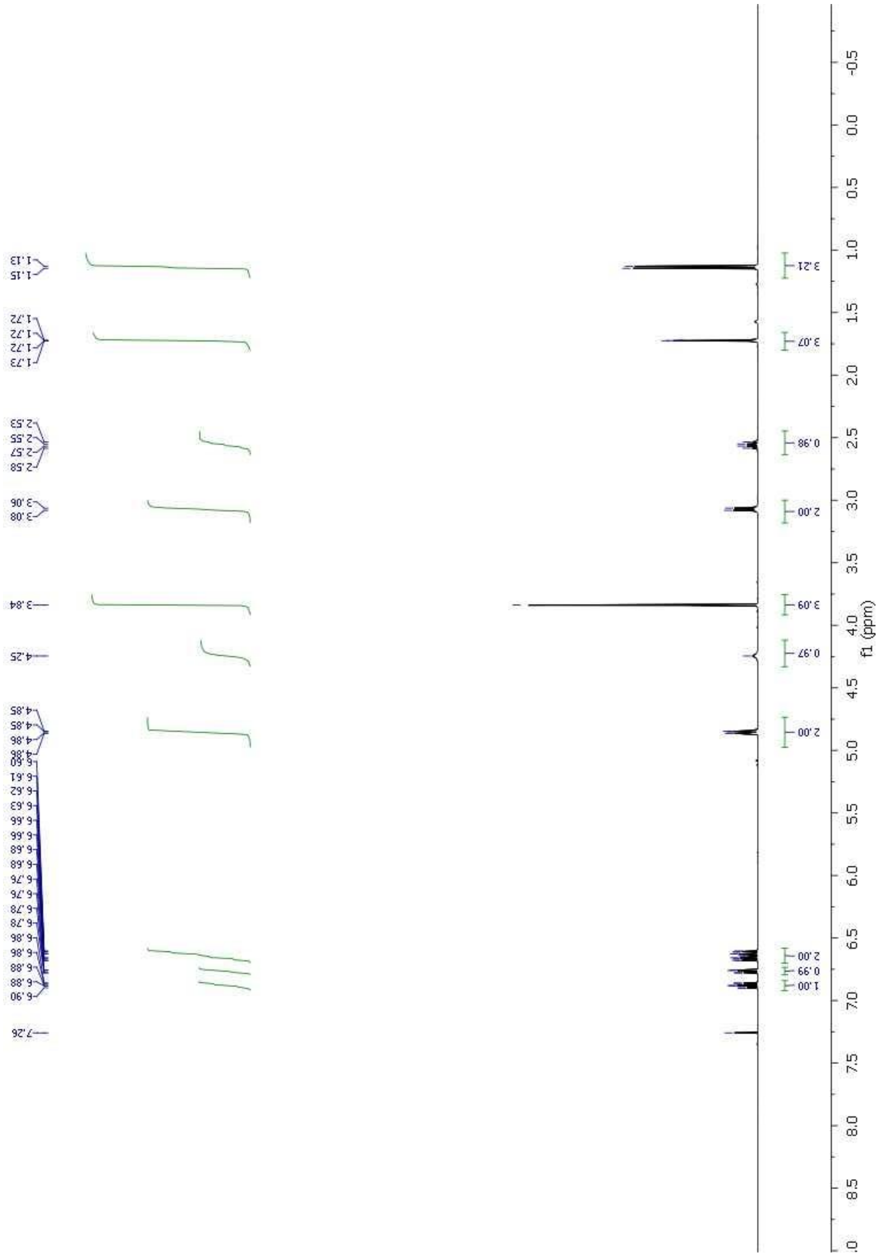

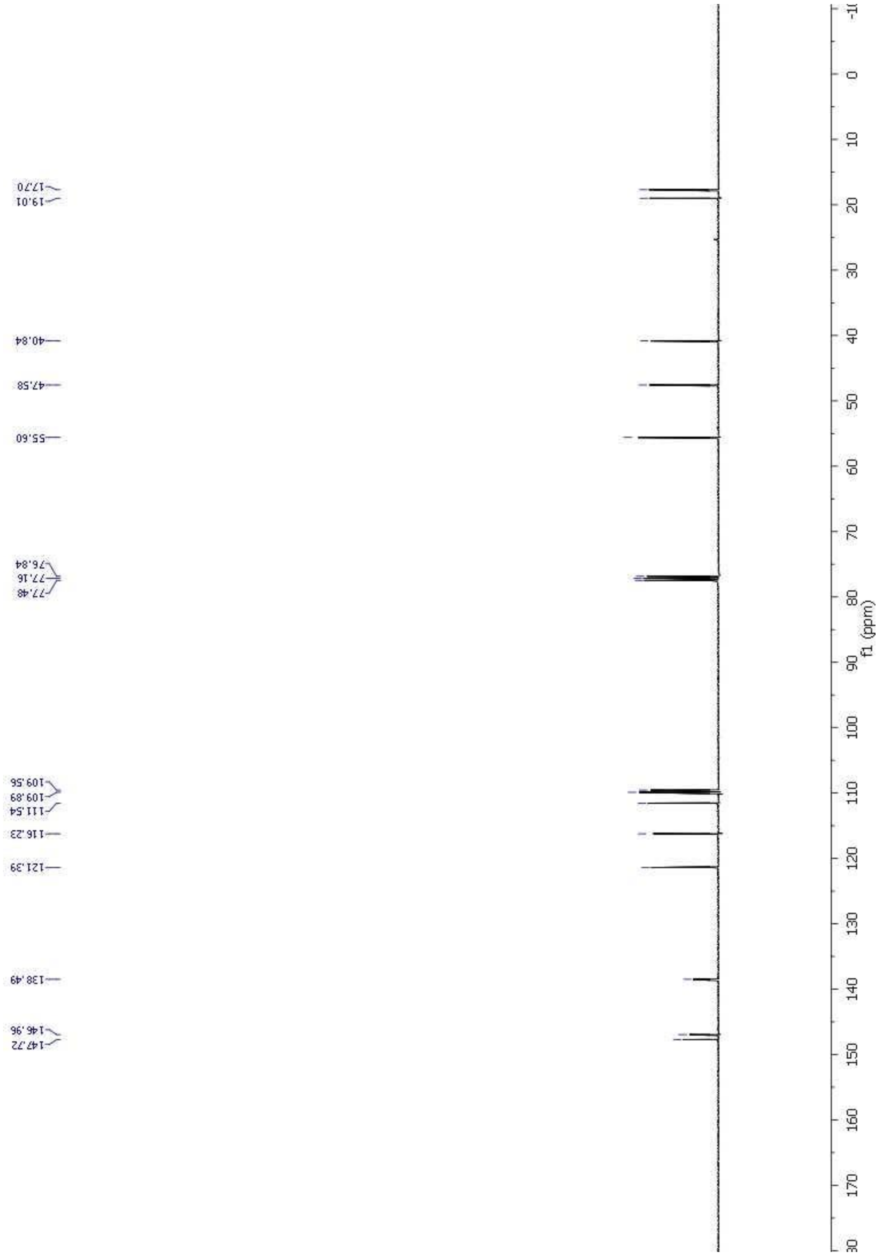
17.70
19.01
40.84
47.58
55.60
76.84
77.16
77.48
109.56
109.89
111.54
116.23
121.39
138.49
146.96
147.72
f1 (ppm)

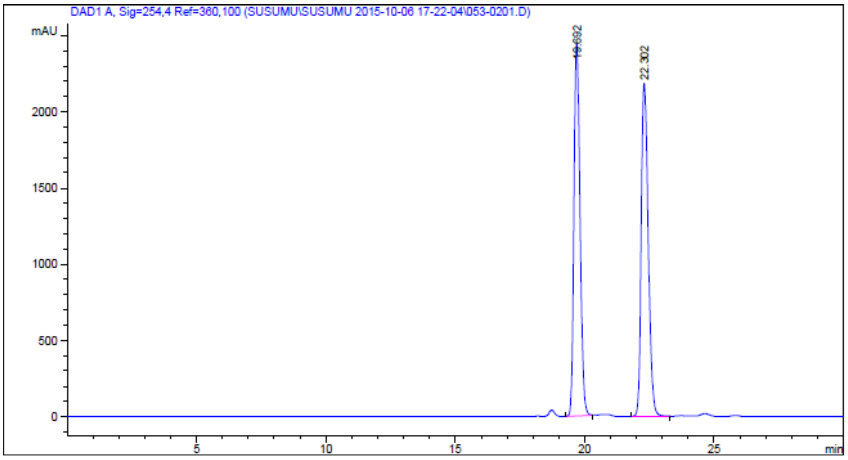

Signal 1: DAD1 A, Sig=254,4 Ref=360,100

Peak #	RetTime [min]	Type	Width [min]	Area [mAU*s]	Height [mAU]	Area %
1	19.692	BB	0.2567	4.00035e4	2447.94849	49.4271
2	22.302	BB	0.2904	4.09309e4	2186.30664	50.5729
Totals :				8.09343e4	4634.25513	

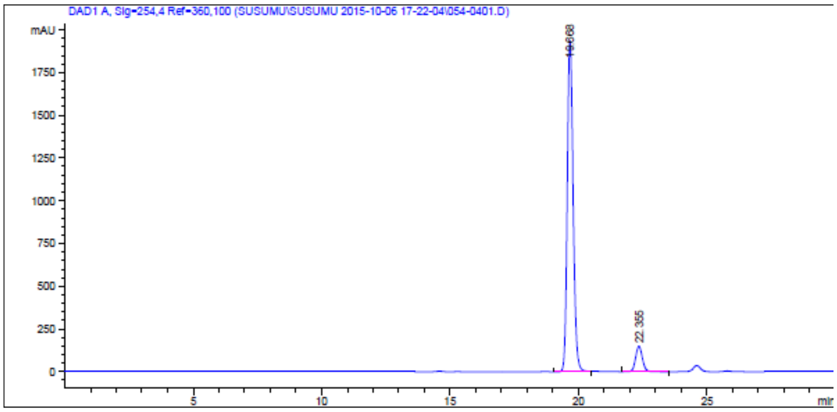

Signal 1: DAD1 A, Sig=254,4 Ref=360,100

Peak #	RetTime [min]	Type	Width [min]	Area [mAU*s]	Height [mAU]	Area %
1	19.667	BB	0.2495	3.11432e4	1937.34961	93.3256
2	22.355	MM	0.2668	2227.29687	139.13069	6.6744
Totals :				3.33705e4	2076.48030	

VII. Experimental Procedure and Crystallographic Material for S1

N-(2-methylbut-3-en-1-yl)-*N*-(4-nitrobenzenesulfonyl)-2-methoxyaniline (S1)

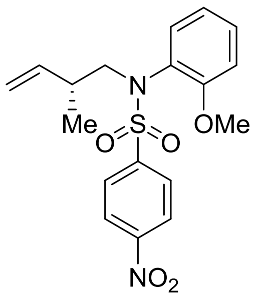

To a solution of (*R*)-*N*-(2-methylbut-3-en-1-yl)-2-methoxyaniline ((*R*)-**5a**) (19.1 mg, 0.100 mmol, 100 mol%) and Et_3N (21 μL, 0.15 mmol, 150 mol%) in CH_2Cl_2 (2.0 mL), 4-nitrobenzenesulfonyl chloride (26.5 mg, 0.12 mmol, 120 mo%) was added. The reaction mixture was stirred for 20 hours at room temperature and concentrated *in vacuo*. The residue was purified by flash column chromatography (SiO_2, 16% EtOAc/hexanes) to furnish the title compound (18.8 mg, 50%) as a pale yellow solid.

^{1}H NMR (400 MHz, $CDCl_3$): δ 8.26 (d, *J* = 9.0 Hz, 2H), 7.79 (d, *J* = 9.0 Hz, 2H), 7.40 – 7.28 (m, 2H), 6.98 (td, *J* = 7.6, 1.3 Hz, 1H), 6.76 (dd, *J* = 8.3, 1.1 Hz, 1H), 5.08 – 4.89 (m, 2H), 3.27 (s, 3H), 2.24 (dt, *J* = 14.0, 7.0 Hz, 1H), 1.04 (s, 3H).

^{13}C NMR (100 MHz, $CDCl_3$): δ 149.78, 146.23, 134.02, 130.42, 128.81, 125.68, 123.60, 121.07, 115.22, 111.74, 55.50, 54.75, 37.28, 29.85.

LRMS (ESI): m/z 377 $[M+H]^+$

FTIR (neat): 2922, 2851, 1605, 1528, 1494, 1463, 1400, 1348, 1308, 1282, 1255, 1164, 1089, 1063, 1043, 1024, 955, 918, 853, 789, 748, 738, 708 cm^{-1}.

X-ray Experimental for $C_{18}H_{20}N_2O_5S$: Crystals grew as clusters of colorless prisms by slow evaporation from Et_2O/pentane. The data crystal was cut from a larger crystal and had approximate dimensions; 0.23 x 0.15 x 0.07 mm. The data were collected on an Agilent Technologies SuperNova Dual Source diffractometer using a μ-focus Cu Kα radiation source (λ = 1.5418Å) with collimating mirror monochromators. A total of 674 frames of data were collected using ω-scans with a scan range of 1° and a counting time of 3 seconds per frame for frames collected with a detector offset of +/- 41° and 12 seconds per frame with frames collected with a detector offset of +/- 108°. The data were collected at 100 K using an Oxford Cryostream low temperature device. Details of crystal data, data collection and structure refinement are listed in Table 1. Data collection, unit cell refinement and data reduction were performed using Agilent Technologies CrysAlisPro V 1.171.37.31.[5] The structure was solved by direct methods using SuperFlip[6] and refined by full-matrix least-squares on F^2 with anisotropic displacement parameters for the non-H atoms using SHELXL-2014/7.[7] Structure analysis was aided by use of the programs PLATON98[8] and WinGX.[9] The hydrogen atoms were calculated in ideal positions with isotropic displacement parameters set to 1.2xUeq of the attached atom (1.5xUeq for methyl hydrogen atoms). The absolute configuration was determined using the method of Flack[10] and confirmed using the Hooft y-parameter method.[11] The Flack parameter refined to -0.05(3), while the Hooft y-parameter refined to 0.01(2).

The function, $\Sigma w(|F_o|^2 - |F_c|^2)^2$, was minimized, where $w = 1/[(\sigma(F_o))^2 + (0.0594*P)^2 + (0.7503*P)]$ and $P = (|F_o|^2 + 2|F_c|^2)/3$. $R_W(F^2)$ refined to 0.111, with R(F) equal to 0.0406 and a goodness of fit, S, = 1.05. Definitions used for calculating R(F), $R_W(F^2)$ and the goodness of fit, S, are

[5]CrysAlisPro. Agilent Technologies (2013). Agilent Technologies UK Ltd., Oxford, UK, SuperNova CCD System, CrysAlicPro Software System, 1.171.37.31.
[6]SuperFlip. (2007). A program for crystal structure solution. Palatinus, L. and Chapuis, G. J. Appl. Cryst. 40, 786-790.
[7]Sheldrick, G. M. (2015). SHELXL-2014/7. Program for the Refinement of Crystal Structures. Acta Cryst., C71, 9-18.
[8]Spek, A. L. (1998). PLATON, A Multipurpose Crystallographic Tool. Utrecht University, The Netherlands.
[9]WinGX 1.64. (1999). An Integrated System of Windows Programs for the Solution, Refinement and Analysis of Single Crystal X-ray Diffraction Data. Farrugia, L. J. J. Appl. Cryst. 32. 837-838.
[10]Flack, H. D. (1983). Acta Cryst A39, 876-881.
[11]Hooft, R. W. W., Straver, L. H. and Spek, A. L. (2008). J. Appl. Cryst. 41, 96-103.

given below.[12] The data were checked for secondary extinction effects but no correction was necessary. Neutral atom scattering factors and values used to calculate the linear absorption coefficient are from the International Tables for X-ray Crystallography (1992).[13] All figures were generated using SHELXTL/PC.[14] Tables of positional and thermal parameters, bond lengths and angles, torsion angles and figures are found elsewhere.

---

[12] $R_w(F^2) = \{\Sigma w(|F_o|^2 - |F_c|^2)^2/\Sigma w(|F_o|)^4\}^{1/2}$ where w is the weight given each reflection.
$R(F) = \Sigma(|F_o| - |F_c|)/\Sigma|F_o|\}$ for reflections with $F_o > 4(\sigma(F_o))$.
$S = [\Sigma w(|F_o|^2 - |F_c|^2)^2/(n - p)]^{1/2}$, where n is the number of reflections and p is the number of refined parameters.

[13] International Tables for X-ray Crystallography (1992). Vol. C, Tables 4.2.6.8 and 6.1.1.4, A. J. C. Wilson, editor, Boston: Kluwer Academic Press.

[14] Sheldrick, G. M. (1994). SHELXTL/PC (Version 5.03). Siemens Analytical X-ray Instruments, Inc., Madison, Wisconsin, USA.

Table S1. Crystal data and structure refinement for **S1**.

Empirical formula	C18 H20 N2 O5 S	
Formula weight	376.42	
Temperature	100(2) K	
Wavelength	1.54184 Å	
Crystal system	monoclinic	
Space group	P 21	
Unit cell dimensions	a = 6.7708(3) Å	α= 90°.
	b = 15.0499(6) Å	β= 98.942(4)°.
	c = 18.2635(7) Å	γ = 90°.
Volume	1838.43(13) $Å^3$	
Z	4	
Density (calculated)	1.360 Mg/m^3	
Absorption coefficient	1.842 mm^{-1}	
F(000)	792	
Crystal size	0.230 x 0.150 x 0.070 mm^3	
Theta range for data collection	2.449 to 74.183°.	
Index ranges	-6<=h<=8, -10<=k<=18, -22<=l<=21	
Reflections collected	7283	
Independent reflections	4742 [R(int) = 0.0178]	
Completeness to theta = 67.684°	99.9 %	
Absorption correction	Semi-empirical from equivalents	
Max. and min. transmission	1.00 and 0.885	
Refinement method	Full-matrix least-squares on F^2	
Data / restraints / parameters	4742 / 1 / 474	
Goodness-of-fit on F^2	1.050	
Final R indices [I>2sigma(I)]	R1 = 0.0406, wR2 = 0.1088	
R indices (all data)	R1 = 0.0430, wR2 = 0.1112	
Absolute structure parameter	-0.05(3)	
Extinction coefficient	n/a	
Largest diff. peak and hole	0.370 and -0.363 e.$Å^{-3}$	

Table S2. Atomic coordinates (x 10^4) and equivalent isotropic displacement parameters (Å^{2}x 10^3) for **S1**. U(eq) is defined as one third of the trace of the orthogonalized U^{ij} tensor.

	x	y	z	U(eq)
C1	-2586(6)	3652(3)	-226(2)	22(1)
C2	-1376(5)	3320(3)	408(2)	24(1)
C3	-2133(6)	2695(3)	847(2)	26(1)
C4	-4088(6)	2391(3)	652(2)	28(1)
C5	-5280(6)	2704(3)	14(3)	29(1)
C6	-4522(6)	3337(3)	-418(2)	26(1)
C7	1684(6)	3428(3)	1245(2)	30(1)
C8	-209(6)	4065(3)	-1088(2)	28(1)
C9	-941(6)	3418(3)	-1727(2)	30(1)
C10	875(6)	3091(4)	-2061(2)	40(1)
C11	-2474(7)	3859(4)	-2295(2)	42(1)
C12	-4219(7)	3562(4)	-2539(2)	54(1)
C13	-548(6)	5623(3)	298(2)	26(1)
C14	-1180(6)	5604(3)	985(2)	30(1)
C15	190(7)	5762(3)	1618(2)	32(1)
C16	2164(6)	5929(3)	1540(2)	29(1)
C17	2796(6)	5965(3)	856(2)	30(1)
C18	1419(6)	5809(3)	230(2)	30(1)
C19	12518(6)	6323(3)	5260(2)	25(1)
C20	11269(6)	6664(3)	4634(2)	26(1)
C21	12028(6)	7309(3)	4206(2)	32(1)
C22	13973(7)	7595(3)	4400(2)	36(1)
C23	15196(7)	7268(3)	5015(3)	35(1)
C24	14466(6)	6626(3)	5442(2)	28(1)
C25	8183(7)	6556(4)	3803(2)	36(1)
C26	10180(7)	5820(4)	6120(2)	38(1)
C27	10873(9)	6396(5)	6785(3)	59(2)
C28	9120(9)	6554(4)	7185(3)	54(1)
C29	7962(10)	7241(4)	7073(3)	67(2)
C30	12662(10)	6117(9)	7299(3)	109(4)
C31	10569(6)	4383(3)	4652(2)	28(1)

C32	8593(6)	4166(3)	4709(2)	30(1)
C33	7221(6)	4062(3)	4069(2)	32(1)
C34	7875(6)	4175(3)	3391(2)	31(1)
C35	9818(6)	4373(3)	3327(2)	33(1)
C36	11185(6)	4486(3)	3963(2)	32(1)
N1	-1844(5)	4313(2)	-687(2)	23(1)
N2	3641(6)	6060(3)	2210(2)	38(1)
N3	11839(5)	5629(3)	5702(2)	28(1)
N4	6389(6)	4104(3)	2710(2)	39(1)
O1	-1747(5)	5872(2)	-1101(2)	35(1)
O2	-4227(4)	5405(2)	-311(2)	31(1)
O3	515(4)	3663(2)	547(1)	26(1)
O4	5394(5)	6210(3)	2143(2)	53(1)
O5	3061(6)	6023(4)	2817(2)	61(1)
O6	11754(5)	4031(3)	6038(2)	44(1)
O7	14221(4)	4577(2)	5279(2)	36(1)
O8	9389(4)	6334(2)	4496(2)	30(1)
O9	4664(5)	4005(4)	2780(2)	69(1)
O10	6971(6)	4172(3)	2118(2)	54(1)
S1	-2261(1)	5350(1)	-505(1)	26(1)
S2	12258(2)	4607(1)	5470(1)	30(1)

Table S3. Bond lengths [Å] and angles [°] for **S1**.

C1-C6	1.386(6)	C14-H14	0.95
C1-C2	1.403(5)	C15-C16	1.389(6)
C1-N1	1.443(5)	C15-H15	0.95
C2-O3	1.367(5)	C16-C17	1.384(6)
C2-C3	1.386(6)	C16-N2	1.468(5)
C3-C4	1.394(6)	C17-C18	1.378(6)
C3-H3	0.95	C17-H17	0.95
C4-C5	1.392(6)	C18-H18	0.95
C4-H4	0.95	C19-C24	1.387(6)
C5-C6	1.387(6)	C19-C20	1.408(6)
C5-H5	0.95	C19-N3	1.439(5)
C6-H6	0.95	C20-O8	1.353(5)
C7-O3	1.437(4)	C20-C21	1.394(6)
C7-H7A	0.98	C21-C22	1.379(6)
C7-H7B	0.98	C21-H21	0.95
C7-H7C	0.98	C22-C23	1.378(7)
C8-N1	1.468(5)	C22-H22	0.95
C8-C9	1.541(6)	C23-C24	1.381(6)
C8-H8A	0.99	C23-H23	0.95
C8-H8B	0.99	C24-H24	0.95
C9-C11	1.503(6)	C25-O8	1.435(5)
C9-C10	1.536(5)	C25-H25A	0.98
C9-H9	1.00	C25-H25B	0.98
C10-H10A	0.98	C25-H25C	0.98
C10-H10B	0.98	C26-N3	1.481(5)
C10-H10C	0.98	C26-C27	1.507(7)
C11-C12	1.277(7)	C26-H26A	0.99
C11-H11	0.95	C26-H26B	0.99
C12-H12A	0.95	C27-C30	1.473(9)
C12-H12B	0.95	C27-C28	1.506(7)
C13-C18	1.385(6)	C27-H27	1.00
C13-C14	1.389(5)	C28-C29	1.294(8)
C13-S1	1.770(4)	C28-H28	0.95
C14-C15	1.385(6)	C29-H29A	0.95

C29-H29B	0.95	C35-C36	1.379(6)
C30-H30A	0.98	C35-H35	0.95
C30-H30B	0.98	C36-H36	0.95
C30-H30C	0.98	N1-S1	1.629(4)
C31-C36	1.394(6)	N2-O4	1.233(5)
C31-C32	1.397(6)	N2-O5	1.234(5)
C31-S2	1.767(4)	N3-S2	1.631(4)
C32-C33	1.384(6)	N4-O9	1.204(5)
C32-H32	0.95	N4-O10	1.211(5)
C33-C34	1.389(6)	O1-S1	1.429(3)
C33-H33	0.95	O2-S1	1.432(3)
C34-C35	1.372(6)	O6-S2	1.434(3)
C34-N4	1.478(5)	O7-S2	1.426(3)

C6-C1-C2	119.7(4)	H7A-C7-H7C	109.5
C6-C1-N1	119.3(4)	H7B-C7-H7C	109.5
C2-C1-N1	121.0(4)	N1-C8-C9	111.1(3)
O3-C2-C3	124.6(4)	N1-C8-H8A	109.4
O3-C2-C1	115.4(4)	C9-C8-H8A	109.4
C3-C2-C1	119.9(4)	N1-C8-H8B	109.4
C2-C3-C4	119.8(4)	C9-C8-H8B	109.4
C2-C3-H3	120.1	H8A-C8-H8B	108.0
C4-C3-H3	120.1	C11-C9-C10	112.1(3)
C5-C4-C3	120.5(4)	C11-C9-C8	110.5(4)
C5-C4-H4	119.8	C10-C9-C8	108.7(3)
C3-C4-H4	119.8	C11-C9-H9	108.5
C6-C5-C4	119.4(4)	C10-C9-H9	108.5
C6-C5-H5	120.3	C8-C9-H9	108.5
C4-C5-H5	120.3	C9-C10-H10A	109.5
C1-C6-C5	120.7(4)	C9-C10-H10B	109.5
C1-C6-H6	119.7	H10A-C10-H10B	109.5
C5-C6-H6	119.7	C9-C10-H10C	109.5
O3-C7-H7A	109.5	H10A-C10-H10C	109.5
O3-C7-H7B	109.5	H10B-C10-H10C	109.5
H7A-C7-H7B	109.5	C12-C11-C9	126.1(5)
O3-C7-H7C	109.5	C12-C11-H11	117.0

C9-C11-H11	117.0	C24-C23-H23	120.4
C11-C12-H12A	120.0	C23-C24-C19	120.3(4)
C11-C12-H12B	120.0	C23-C24-H24	119.8
H12A-C12-H12B	120.0	C19-C24-H24	119.8
C18-C13-C14	121.4(4)	O8-C25-H25A	109.5
C18-C13-S1	119.3(3)	O8-C25-H25B	109.5
C14-C13-S1	119.3(3)	H25A-C25-H25B	109.5
C15-C14-C13	119.3(4)	O8-C25-H25C	109.5
C15-C14-H14	120.3	H25A-C25-H25C	109.5
C13-C14-H14	120.3	H25B-C25-H25C	109.5
C14-C15-C16	118.5(4)	N3-C26-C27	111.2(4)
C14-C15-H15	120.8	N3-C26-H26A	109.4
C16-C15-H15	120.8	C27-C26-H26A	109.4
C17-C16-C15	122.5(4)	N3-C26-H26B	109.4
C17-C16-N2	118.8(4)	C27-C26-H26B	109.4
C15-C16-N2	118.8(4)	H26A-C26-H26B	108.0
C18-C17-C16	118.5(4)	C30-C27-C28	111.6(4)
C18-C17-H17	120.7	C30-C27-C26	118.1(7)
C16-C17-H17	120.7	C28-C27-C26	108.2(5)
C17-C18-C13	119.8(4)	C30-C27-H27	106.0
C17-C18-H18	120.1	C28-C27-H27	106.0
C13-C18-H18	120.1	C26-C27-H27	106.0
C24-C19-C20	120.3(4)	C29-C28-C27	123.6(6)
C24-C19-N3	118.6(4)	C29-C28-H28	118.2
C20-C19-N3	121.1(4)	C27-C28-H28	118.2
O8-C20-C21	124.7(4)	C28-C29-H29A	120.0
O8-C20-C19	116.5(4)	C28-C29-H29B	120.0
C21-C20-C19	118.8(4)	H29A-C29-H29B	120.0
C22-C21-C20	119.7(4)	C27-C30-H30A	109.5
C22-C21-H21	120.2	C27-C30-H30B	109.5
C20-C21-H21	120.2	H30A-C30-H30B	109.5
C21-C22-C23	121.8(4)	C27-C30-H30C	109.5
C21-C22-H22	119.1	H30A-C30-H30C	109.5
C23-C22-H22	119.1	H30B-C30-H30C	109.5
C22-C23-C24	119.2(4)	C36-C31-C32	121.2(4)
C22-C23-H23	120.4	C36-C31-S2	119.8(3)

C32-C31-S2	118.9(3)	O5-N2-C16	118.2(4)
C33-C32-C31	119.2(4)	C19-N3-C26	118.8(4)
C33-C32-H32	120.4	C19-N3-S2	117.1(3)
C31-C32-H32	120.4	C26-N3-S2	119.6(3)
C32-C33-C34	118.3(4)	O9-N4-O10	124.2(4)
C32-C33-H33	120.9	O9-N4-C34	117.6(4)
C34-C33-H33	120.9	O10-N4-C34	118.2(4)
C35-C34-C33	123.1(4)	C2-O3-C7	116.3(3)
C35-C34-N4	118.7(4)	C20-O8-C25	118.0(3)
C33-C34-N4	118.2(4)	O1-S1-O2	120.1(2)
C34-C35-C36	118.8(4)	O1-S1-N1	107.48(18)
C34-C35-H35	120.6	O2-S1-N1	107.66(19)
C36-C35-H35	120.6	O1-S1-C13	107.3(2)
C35-C36-C31	119.4(4)	O2-S1-C13	107.27(19)
C35-C36-H36	120.3	N1-S1-C13	106.24(18)
C31-C36-H36	120.3	O7-S2-O6	119.6(2)
C1-N1-C8	118.3(3)	O7-S2-N3	107.4(2)
C1-N1-S1	117.0(3)	O6-S2-N3	108.1(2)
C8-N1-S1	120.9(3)	O7-S2-C31	107.00(19)
O4-N2-O5	122.9(4)	O6-S2-C31	107.6(2)
O4-N2-C16	118.9(4)	N3-S2-C31	106.5(2)

Table S4. Anisotropic displacement parameters ($Å^2$x 10^3) for **S1**. The anisotropic displacement factor exponent takes the form: $-2\pi^2[h^2 a^{*2}U^{11} + ... + 2 h k a^* b^* U^{12}]$

	U^{11}	U^{22}	U^{33}	U^{23}	U^{13}	U^{12}
C1	26(2)	22(2)	21(2)	-2(2)	8(1)	5(2)
C2	22(2)	22(2)	26(2)	-4(2)	3(1)	3(2)
C3	36(2)	19(2)	24(2)	1(2)	8(1)	3(2)
C4	34(2)	22(2)	30(2)	1(2)	12(2)	-4(2)
C5	24(2)	27(2)	38(2)	-2(2)	9(2)	-2(2)
C6	24(2)	28(2)	26(2)	-3(2)	2(1)	2(2)
C7	28(2)	37(2)	23(2)	-1(2)	-1(2)	5(2)
C8	28(2)	35(2)	25(2)	-1(2)	11(2)	-2(2)
C9	32(2)	30(2)	28(2)	0(2)	7(2)	-4(2)
C10	33(2)	52(3)	37(2)	-5(2)	8(2)	9(2)
C11	47(2)	57(3)	22(2)	-1(2)	2(2)	6(2)
C12	43(2)	74(4)	39(2)	-15(2)	-8(2)	17(2)
C13	33(2)	17(2)	28(2)	1(2)	9(2)	1(2)
C14	28(2)	31(2)	33(2)	0(2)	12(2)	1(2)
C15	39(2)	32(2)	27(2)	-3(2)	16(2)	3(2)
C16	34(2)	23(2)	28(2)	-5(2)	4(2)	-3(2)
C17	32(2)	26(2)	33(2)	-2(2)	11(2)	-5(2)
C18	36(2)	25(2)	31(2)	3(2)	15(2)	-2(2)
C19	26(2)	28(2)	23(2)	-1(2)	7(1)	-2(2)
C20	31(2)	28(2)	21(2)	-4(2)	8(1)	2(2)
C21	41(2)	28(2)	29(2)	1(2)	6(2)	3(2)
C22	47(2)	27(2)	37(2)	-2(2)	17(2)	-4(2)
C23	33(2)	33(3)	38(2)	-7(2)	10(2)	-6(2)
C24	28(2)	28(2)	29(2)	-4(2)	9(2)	0(2)
C25	39(2)	38(2)	27(2)	0(2)	0(2)	6(2)
C26	39(2)	48(3)	30(2)	-8(2)	16(2)	-10(2)
C27	59(3)	84(5)	34(2)	-23(3)	10(2)	2(3)
C28	78(3)	55(3)	29(2)	-7(2)	12(2)	12(3)
C29	86(4)	60(4)	56(3)	-13(3)	18(3)	-13(3)
C30	74(4)	213(12)	43(3)	-42(5)	21(3)	-36(6)
C31	30(2)	26(2)	29(2)	1(2)	2(2)	3(2)

C32	35(2)	30(2)	26(2)	3(2)	6(2)	-5(2)
C33	29(2)	35(3)	33(2)	-2(2)	5(2)	-3(2)
C34	37(2)	29(2)	25(2)	-5(2)	3(2)	3(2)
C35	37(2)	39(3)	26(2)	-3(2)	8(2)	5(2)
C36	30(2)	37(2)	31(2)	0(2)	8(2)	3(2)
N1	24(2)	23(2)	24(1)	1(1)	7(1)	-1(1)
N2	45(2)	38(2)	31(2)	-9(2)	6(2)	-2(2)
N3	31(2)	36(2)	18(1)	0(1)	6(1)	-1(2)
N4	36(2)	46(2)	32(2)	-10(2)	-1(1)	0(2)
O1	47(2)	29(2)	31(1)	10(1)	8(1)	-2(1)
O2	27(1)	28(2)	40(2)	2(1)	7(1)	9(1)
O3	26(1)	27(2)	25(1)	2(1)	0(1)	-2(1)
O4	33(2)	81(3)	46(2)	-20(2)	5(1)	-6(2)
O5	57(2)	97(4)	30(2)	-14(2)	8(1)	-19(2)
O6	53(2)	43(2)	32(2)	15(2)	-7(1)	-12(2)
O7	30(1)	31(2)	45(2)	1(2)	-2(1)	4(1)
O8	28(1)	37(2)	25(1)	1(1)	1(1)	1(1)
O9	41(2)	119(4)	44(2)	-16(2)	-2(2)	-15(2)
O10	62(2)	71(3)	27(2)	-7(2)	-1(1)	-6(2)
S1	29(1)	23(1)	27(1)	5(1)	6(1)	3(1)
S2	33(1)	29(1)	27(1)	6(1)	-1(1)	-3(1)

Table S5. Hydrogen coordinates (x 10^4) and isotropic displacement parameters (Å^{2}x 10^3) for **S1**.

	x	y	z	U(eq)
H3	-1321	2474	1280	31
H4	-4611	1967	956	33
H5	-6602	2485	-124	35
H6	-5337	3558	-851	32
H7A	967	3602	1649	45
H7B	2973	3737	1300	45
H7C	1910	2785	1262	45
H8A	878	3782	-741	34
H8B	338	4607	-1291	34
H9	-1577	2895	-1519	36
H10A	1530	3599	-2259	61
H10B	432	2669	-2461	61
H10C	1821	2798	-1676	61
H11	-2110	4409	-2492	51
H12A	-4650	3014	-2359	64
H12B	-5088	3889	-2900	64
H14	-2538	5483	1022	36
H15	-211	5758	2094	38
H17	4150	6095	818	36
H18	1817	5829	-246	36
H21	11210	7550	3783	39
H22	14484	8030	4102	43
H23	16525	7482	5144	41
H24	15303	6391	5864	34
H25A	7976	7200	3775	53
H25B	6886	6256	3767	53
H25C	8861	6363	3394	53
H26A	9645	5255	6284	45
H26B	9088	6125	5791	45
H27	11206	6987	6585	70

H28	8850	6126	7538	65
H29A	8198	7680	6723	80
H29B	6877	7307	7342	80
H30A	13155	6616	7621	163
H30B	13703	5928	7014	163
H30C	12317	5621	7602	163
H32	8196	4091	5182	36
H33	5867	3917	4093	39
H35	10215	4431	2852	40
H36	12535	4633	3932	39

Table 6. Torsion angles [°] for **S1**.

C6-C1-C2-O3	179.3(3)	C20-C19-C24-C23	0.4(6)
N1-C1-C2-O3	-0.3(5)	N3-C19-C24-C23	177.6(4)
C6-C1-C2-C3	-1.4(6)	N3-C26-C27-C30	-51.5(8)
N1-C1-C2-C3	179.0(4)	N3-C26-C27-C28	-179.4(5)
O3-C2-C3-C4	-180.0(4)	C30-C27-C28-C29	134.4(8)
C1-C2-C3-C4	0.7(6)	C26-C27-C28-C29	-94.0(8)
C2-C3-C4-C5	0.7(6)	C36-C31-C32-C33	-0.7(7)
C3-C4-C5-C6	-1.4(6)	S2-C31-C32-C33	174.7(4)
C2-C1-C6-C5	0.6(6)	C31-C32-C33-C34	0.2(7)
N1-C1-C6-C5	-179.8(4)	C32-C33-C34-C35	0.9(8)
C4-C5-C6-C1	0.8(6)	C32-C33-C34-N4	-177.3(4)
N1-C8-C9-C11	63.7(5)	C33-C34-C35-C36	-1.6(8)
N1-C8-C9-C10	-172.9(4)	N4-C34-C35-C36	176.6(4)
C10-C9-C11-C12	112.2(5)	C34-C35-C36-C31	1.0(7)
C8-C9-C11-C12	-126.4(5)	C32-C31-C36-C35	0.1(7)
C18-C13-C14-C15	-0.8(7)	S2-C31-C36-C35	-175.3(4)
S1-C13-C14-C15	175.9(3)	C6-C1-N1-C8	-113.9(4)
C13-C14-C15-C16	-0.6(7)	C2-C1-N1-C8	65.7(5)
C14-C15-C16-C17	1.8(7)	C6-C1-N1-S1	87.7(4)
C14-C15-C16-N2	-177.5(4)	C2-C1-N1-S1	-92.7(4)
C15-C16-C17-C18	-1.6(7)	C9-C8-N1-C1	71.1(5)
N2-C16-C17-C18	177.7(4)	C9-C8-N1-S1	-131.4(3)
C16-C17-C18-C13	0.2(7)	C17-C16-N2-O4	0.9(7)
C14-C13-C18-C17	1.0(7)	C15-C16-N2-O4	-179.8(5)
S1-C13-C18-C17	-175.7(4)	C17-C16-N2-O5	-179.7(5)
C24-C19-C20-O8	-179.9(4)	C15-C16-N2-O5	-0.4(7)
N3-C19-C20-O8	3.0(6)	C24-C19-N3-C26	117.1(4)
C24-C19-C20-C21	-0.1(6)	C20-C19-N3-C26	-65.7(5)
N3-C19-C20-C21	-177.2(4)	C24-C19-N3-S2	-86.9(4)
O8-C20-C21-C22	-179.9(4)	C20-C19-N3-S2	90.3(4)
C19-C20-C21-C22	0.3(6)	C27-C26-N3-C19	-73.9(6)
C20-C21-C22-C23	-0.8(7)	C27-C26-N3-S2	130.7(4)
C21-C22-C23-C24	1.0(7)	C35-C34-N4-O9	-173.8(5)
C22-C23-C24-C19	-0.8(7)	C33-C34-N4-O9	4.4(7)

C35-C34-N4-O10	4.2(7)	C14-C13-S1-O2	17.0(4)
C33-C34-N4-O10	-177.5(5)	C18-C13-S1-N1	78.9(4)
C3-C2-O3-C7	-7.7(6)	C14-C13-S1-N1	-97.9(4)
C1-C2-O3-C7	171.6(3)	C19-N3-S2-O7	40.9(3)
C21-C20-O8-C25	10.0(6)	C26-N3-S2-O7	-163.2(3)
C19-C20-O8-C25	-170.2(4)	C19-N3-S2-O6	171.3(3)
C1-N1-S1-O1	-169.4(3)	C26-N3-S2-O6	-32.9(4)
C8-N1-S1-O1	32.9(3)	C19-N3-S2-C31	-73.4(3)
C1-N1-S1-O2	-38.6(3)	C26-N3-S2-C31	82.4(3)
C8-N1-S1-O2	163.7(3)	C36-C31-S2-O7	-19.3(5)
C1-N1-S1-C13	76.0(3)	C32-C31-S2-O7	165.2(4)
C8-N1-S1-C13	-81.7(3)	C36-C31-S2-O6	-149.0(4)
C18-C13-S1-O1	-35.8(4)	C32-C31-S2-O6	35.5(4)
C14-C13-S1-O1	147.4(4)	C36-C31-S2-N3	95.3(4)
C18-C13-S1-O2	-166.2(3)	C32-C31-S2-N3	-80.2(4)

Figure S1. View of molecule **S1** showing the atom labeling scheme. Displacement ellipsoids are scaled to the 50% probability level.

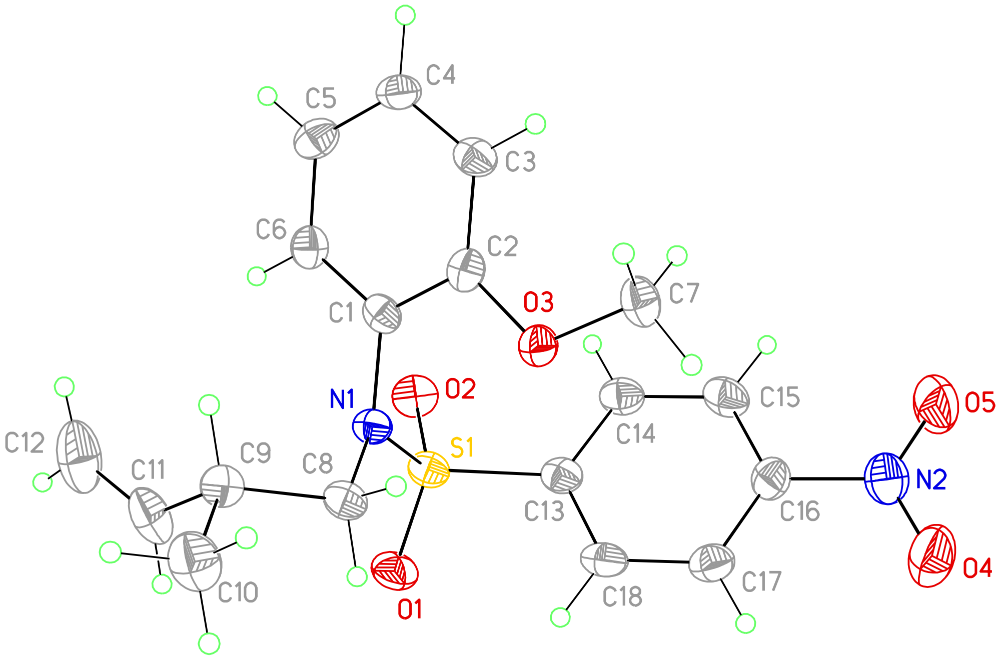

Supplement: Supplementary file 1 [file SC-007-C5SC03854E-s001.pdf]
